# Star-Shaped Conjugated Systems

**DOI:** 10.3390/ma3053218

**Published:** 2010-05-19

**Authors:** Heiner Detert, Matthias Lehmann, Herbert Meier

**Affiliations:** 1 Institute of Organic Chemistry, University of Mainz, Duesbergweg 10-14, D-55099 Mainz, Germany; E-Mail: detert@uni-mainz.de (H.D.); 2 Institute of Chemistry, Chemnitz University of Technology, Straße der Nationen 62, D-09111 Chemnitz, Germany; E-Mail: matthias.lehmann@chemie.tu-chemnitz.de (M.L.)

**Keywords:** [n]stars, conjugation, CC coupling, optoelectronics

## Abstract

The present review deals with the preparation and the properties of star-shaped conjugated compounds. Three, four or six conjugated arms are attached to cross-conjugated cores, which consist of single atoms (B, C^+^, N), benzene or azine rings or polycyclic ring systems, as for example triphenylene or tristriazolotriazine. Many of these shape-persistent [n]star compounds tend to π-stacking and self-organization, and exhibit interesting properties in materials science: Linear and non-linear optics, electrical conductivity, electroluminescence, formation of liquid crystalline phases, *etc.*

Table of Content
1. Introduction
2. Molecular Architecture and Conjugation
3. Three-arm Systems with one Central Atom
3.1. [3]Star Systems with Methylium Core
3.2. [3]Star Systems with Boron Core
3.3. [3]Star Compounds with Nitrogen Core
4. Star Compounds with a Benzene Core
4.1. Three-armed Stars (C-4)
4.1.1. Structure and Conjugation
4.1.2. Synthesis
4.1.3. Three-arm Stars with Benzene Centers and Materials Science
4.2. Four-arm Systems-Tetrasubstituted Benzene (C-5)
4.2.1. Structure and Conjugation
4.2.2. Synthesis
4.2.3. Four-arm Stars with Benzene Centers and Materials Science
4.3. Six-arm Systems-Hexasubstituted Benzenes (C-6)
4.3.1. Structure and Conjugation of Parent Systems
4.3.2. Synthesis of the Parent Systems
4.3.3. Hexaarm Stars with Benzene Centers and Materials Science
5. Compounds with Heterocyclic Cores
5.1. Pyridine-based Stars (C-7-A-6, C-7-A-7)
5.2. Stars with a Pyrimidine Core
5.3. Pyrazine as Core (C-10-A-6)
5.4. 1,3,5-Triazine as Core
5.5. Borazine as a Core
6. Condensed Ring Systems as Cores
6.1. Triphenylene Star Compounds (C-12)
6.1.1. Structures and Synthesis
6.1.2. Triphenylene Derivatives and Materials Science
6.2. Hexaazatriphenylene (C-13-A-3 and C-13-A-6)
6.3. Triazatruxenes (C-14-A-3 and C-14-A-8)
6.4. Tristriazolotriazines (C-15-A-3 and C-15-A-9)
7. Summary and Conclusion

## 1. Introduction

Because of their interesting electrical, optical and optoelectronic properties, conjugated oligomers represent target compounds for many applications in materials science [1,2,3,4,5,6,7,8,9,10,11,12,13,14,15,16,17,18]. Moreover, they are model compounds for the corresponding polymers.

**Figure 1:**
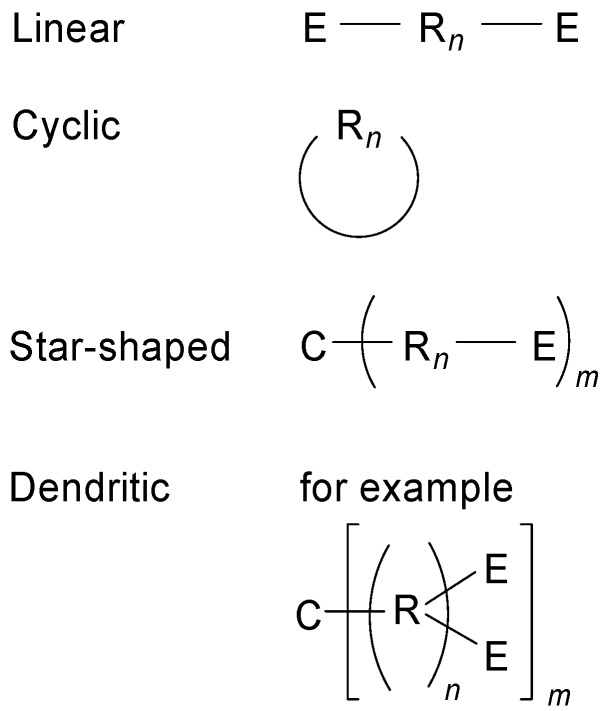
Structure types of conjugated oligomers.

The conjugated oligomers can have linear, cyclic, star-shaped or dendritic structures (Figure 1). Apart from the repeat(ing) unit R, they can contain a core unit C and end groups E.

**Figure 2:**
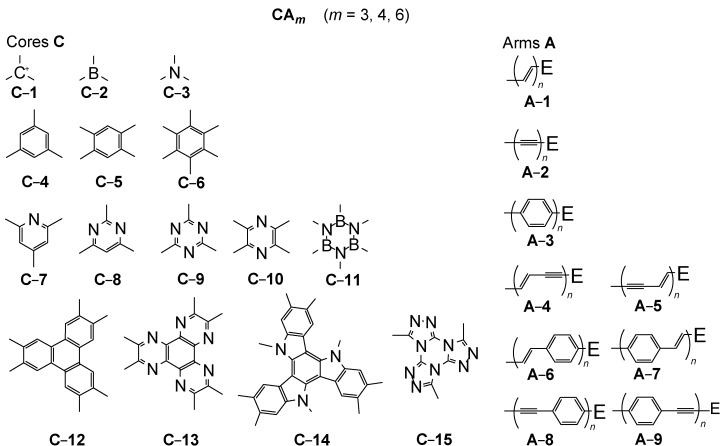
Selected building blocks of star-shaped conjugated oligomers **CA*_m_*** (cores **C**, arms **A**).

The present article is focused on characteristic star-shaped systems **CA***_m_*, so-called [m]stars, in which m identical arms **A**≡R*_n_*E (*m* = 3,4,6) are attached to the central core **C**. Each arm **A** has the same number *n* of repeat units R, which can be assigned as generation *n*. Figure 2 summarizes the most important cores **C** and arms **A.** Less regular structures, which do not have identical arms, are normally not considered here.

The cores guarantee a cross-conjugation. Even single atoms, such as sp^2^-C, B or N (**C-1** − **C-3**), can fulfill this precondition. More important are cores, which consist of benzene (**C-4** − **C-6**), azine (**C-7** − **C-10**) or borazine rings (**C-11**). The special arrangement of four conjugated arms on **C-5** or **C-10** is called a cruciform. Moreover, condensed ring systems, as for example triphenylenes (**C-12**), hexaazatriphenylenes (**C-13**), triazatruxenes (**C-14**) or tristriazolotriazines (**C-15**) have to be considered. The arms **A** are constructed by simple (**A-1** − **A-3**) or composed (**A-4** − **A-9**) repeat units, which convey a linear conjugation. The selected building blocks **C-1** − **C-15** and **A-1** − **A-9** would lead to 15 × 9 = 135 star-shaped oligomer series **CA*_m_***. Many of them are still unknown − despite of intense efforts in this field in the previous two decades.

The synthetic approach to the star-shaped conjugated oligomers **CA*_m_*** comprises convergent and divergent strategies, protecting group techniques and orthogonal techniques-like in the other classes of conjugated oligomers [1,2,3,4,5,6,7,8,9,10,11,12,13,14,15,16,17,18]. Most important are catalyzed CC coupling reactions, such as Heck, Sonogashira or Suzuki reactions. Condensation reactions for the generation of CC double bonds and oxidative couplings of terminal alkynes play also an important role in this context.

## 2. Molecular Architecture and Conjugation

**Figure 3:**
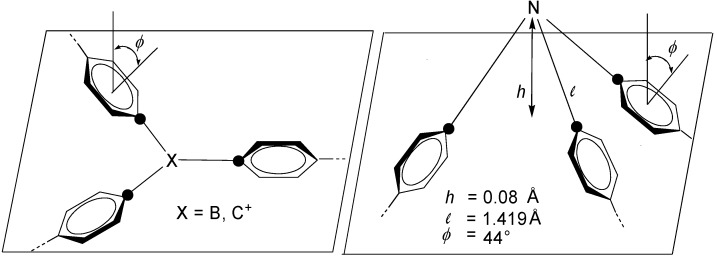
Molecular architecture of oligomers having **C-1**−**C-3** cores and **A-3**, **A-7** or **A-9** arms. The numbers in the tertiary amine structure are related to the crystal structure analysis of tri-phenylamine.

An efficient π-conjugation requires a planar or almost planar geometry of the molecules. Figure 3 illustrates the planarity of carbenium centers **C-1** and boranes **C-2** in contrast to the pyramidal structure of tertiary amine centers **C-3**. The benzene rings of the arms **A**, which are attached to the cores **C-1−C-3**, are twisted by steric reasons. The corresponding dihedral angle *ϕ* is defined as the average angle between the planes of the three benzene rings and the plane, which is determined by the three marked (•) *ipso*-C atoms.

Triphenylborane can be taken as a model compound. According to its crystal structure analysis, the coplanar B-C bonds have an average length of 1.577 Å, their trigonal arrangement is characterized by bond angles of 120 ± 0.4° and the average torsion angle *ϕ* amounts to 30° [19]. This geometry guarantees the cross-conjugation by overlap of the p_z_ (B) orbital and the π and π* orbitals of the three arms.

Triphenylmethylium cores **C-1** have a corresponding molecular architecture, which was established for various triphenylmethane dyes [20].

The situation seems to be different for the amine cores **C-3**. However, the pyramidal structure of triarylamines differs only very slightly from the totally planar conformation, which represents the transition state of the N-inversion. Triphenylamine as model compound has in the crystalline state a very small height *h* = 0.08 Å of the pyramide (Figure 3) [21]. A histogram of the *h* values of all triarylamine structures, listed in the Cambridge data file, reveals 0.00 ≤ *h* ≤ 0.18 Å with a mean value of 0.053 Å and median of 0.040 Å. The dihedral angles range from *ϕ* = 13 to 70° with a mean value of 40.8° and a median of 40.9° [22]. Thus, the cross-conjugation of oligomers with **C-3** cores and **A-3**, **A-7** or **A-9** arms (Figure 2) seems to be guaranteed, which is particularly important for push-pull systems.

All other cores **C-4**−**C-12** are planar rings or ring systems. Three, four or six attached benzene rings lead to twist angles *ϕ* , which represent a compromise between the maximum conjugation for *ϕ* = 0° and minimum steric interaction for *ϕ* = 90°. Other bond rotations in the arms **A-1**−**A-9** have very low energy barriers. The corresponding minima on the energy hypersurface are located in flat wells of planar conformations.

A critical question arises only for continued torsions along oligo(1,4-phenylene) [OP] arms **A-3**. Based on perturbation theory, the decrease of resonance can be described by cos^2^*ϕ* for each torsion angle *ϕ* [23]. The resonance of an OP chain of *n* benzene rings has therefore the attenuation factor (cos^2^*ϕ*)*^n^*^-1^. Thus, an average torsion angle *ϕ* = 23°, which is characteristic for unsubstituted OP chains, reduces the resonance in a 1,4-sexiphenyl chain (*n* = 6) to 50 %. Moreover, an angle *ϕ* = 55.7° would implicate already for *n* = 3 a 90%decrease of the resonance energy [24].

Detailed DFT studies on 1,4-phenylene-ethynylene and 1,4-phenylene-butadiynylene chains revealed that a sufficiently large *ϕ* between two successive segments breaks the through-bond and through-space conjugation and yields weakly coupled chain segments as chromophoric units [25,26,27]. The corresponding function of the electronic coupling *EC* (*ϕ*) has an inflection point whose value *ϕ* can be used as cutoff angle for the conjugation. Due to the dilution of the wave function for increasing segment length, the cutoff angles decrease with increasing numbers *n* of repeat units. Thus, the effective conjugation length in the arm of a compound **CA*_m_***≡**C(R*_n_*E)*_m_*** can be smaller than *n*. In addition to this effect of statistical torsions, the conjugation of planar as well as non-planar arms shows a saturation phenomenon as any conjugated oligomer chain [14,28]. The effective conjugation length *n*_ECL_ defines the number of repeat units, whose exceeding does not lead to a further change of properties such as absorption, fluorescence, *etc.* [28]. The convergence effect is a consequence of different bond lengths and resonance integrals in the chain. Torsions along the chain accelerate this convergence, real kinks can stop the conjugation [14].

In “normal” series of conjugated oligomers, *λ*_max_(*n*) of long-wavelength absorption or emission bands increases monotonously with increasing numbers *n* of repeat units and approaches to a limiting value *λ*_∞_ [14,29]. The earlier used hyperbolic approximation, in which the excitation energy *E* is a function of the reciprocal number of repeat units *n*^-1^, does not sufficiently represent the saturation phenomenon. Some time ago, Meier *et al*. [28] suggested empirical exponential functions for the transition energy *E*(*n*), the *λ* (*n*) values, and the effective conjugation length (Equations 1-3).

*E* (*n*) = *E*_∞_ + (*E*_1_-*E*_∞_)^-a(*n*-1)^ (1)

*λ* (*n*) = *λ*_∞_ − (*λ*_∞_-*λ*_1_) e^-b(*n*-1)^ (2)

*n*_ECL_ = ln (*λ*_∞_-*λ*_1_) · b^-1^ + 1 (3)

*λ*_1_, *E*_1_ : values of the monomer.

*λ*_∞_, *E*_∞_: values of the convergence limit; a, b: parameters optimized by the method of least squares.

Although 0→0 transitions should ideally be used for *λ*, the *λ*_max_ values proved to be satisfactory in most cases. An example is shown in Figure 6.

Recently Gierschner *et al*. [30] and Bednarz, Bäuerle *et al*. [31] proposed semi- to non-empirical equations for the long-wavelength band of conjugated oligomers. The Bednarz algorithm is based on Frenkel exciton models. Its accuracy of interpolations is high; however, the prediction of *λ*_∞_ and *E*_∞_ by extrapolation can be problematic [17,32].

The optical band gap *E*(*n*) and its limiting value *E*_∞_ is a very important feature for many applications of conjugated oligomers in materials science.

**Figure 4:**
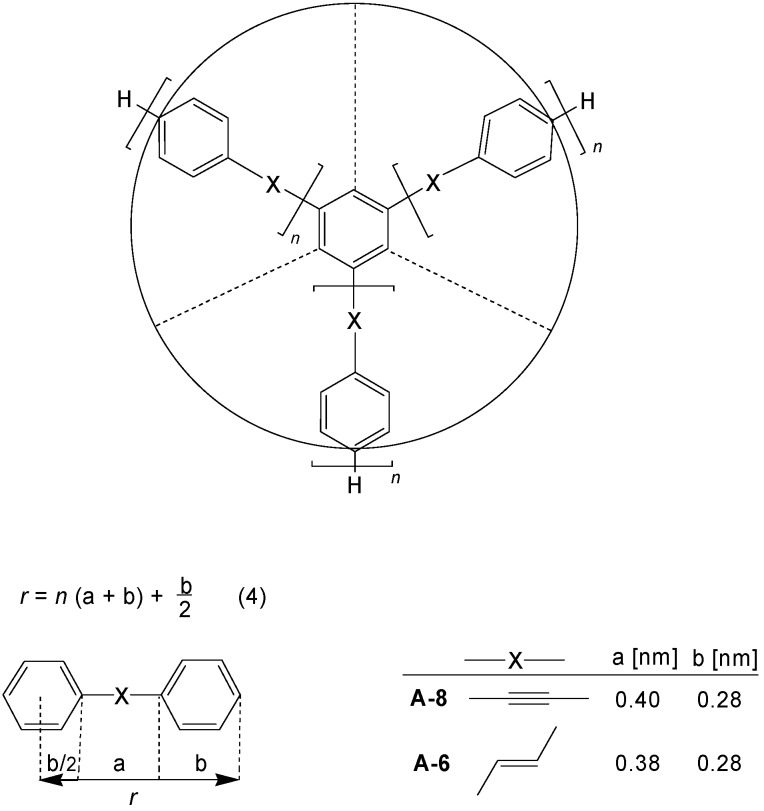
Disc-like shape of [3]star compounds with a benzene core **C-4** and **A-6** or **A-8** arms in 1,3,5-position. The diameter of the discs amounts to *d =* 2*n* · a + (2*n*+1)b for the *n*th generation.

Due to their monodispersity and rigidity, star-shaped oligomers **CA*_m_*** have an exactly defined size and shape. Figure 4 demonstrates the situation for benzene cores **C-4** with three arms **A-6** or **A-8**. The radius *r* of the discs can be calculated by equation (4) and the parameters a and b given in Figure 4 [14]. Accordingly the diameter of these discs increases from about 1.6 nm for the first generation (*n* = 1) to about 5.6 nm for the fourth generation (*n* = 4). Torsions of the benzene rings do not change the size of the nanoparticles.

Neighboring olefinic double bonds, in the arms **A-4−A-6** can have *cisoid* or *transoid* orientations, which have virtually the same energy content. Solely in **A-1**, the energy of the *transoid* conformations is significantly lower.

Thus, a variety of conformers is present in systems, which have *trans* configured olefinic double bonds in their repeat units. Table 1 summarizes the number of *transoid*/*cisoid* conformers. All these conformers contribute for example to the UV/Vis absorption. However, the NMR spectra contain only one set of signals because the equilibration of the conformers is fast in terms of the NMR time scale.

**Table 1:** Number of rotamers in [3]star compounds with different symmetry and arms, which contain repeat units with *trans* configured olefinic double bonds.

Generation *n*	Core Symmetry *D*_3h _*C*_2v _*C*_s_
1 2 3 4	2 4 8 12 32 64 88 256 512 696 512 1024

The number of conformers amounts to 2^3*n*^ in the *C*_s_ and to 2^3*n*-1^ in the *C*_2__ν_ case. Cores with *D*_3h_ symmetry (for example 1,3,5-trisubstituted benzenes or 2,4,6-trisubstituted 1,3,5-triazines) lead to a number Z(*n*) of conformers, which can be calculated by the recursive formula of equation 5.

Z(*n*) = 2^3^ [Z(*n*-1)-1]    *n* = 2, 3, 4, ... Z(2) = 12 (5)

## 3. Three-Arm Systems with One Central Atom

### 3.1. [3]Star Systems with Methylium Core

**Figure 5:**
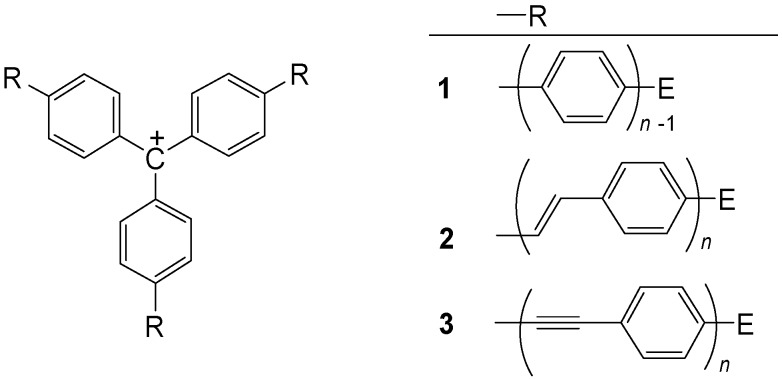
Triphenylmethylium structures with extended conjugation.

Triphenylmethane dyes, which should be better called triphenylmethine dyes [37], are an important class of organic dyes. The extension of the conjugation in their arms can lead to NIR dyes. Figure 5 shows such [3]star compounds. The methylium salts **1-3** can be prepared from the corresponding carbinols, their ethers or esters. A reasonable stability can only be expected for push-pull systems with electron donating end groups E.

Several compounds **1** (*n* = 2, R = H, alkoxy, dialkylamino) [33,34,35], **2** (*n* = 1, R = H, alkoxy, dialkylamino) [36,37,38,39,40] and **3** (*n* = 1, R = H) [36] are known. The effect of cross-conjugation in the center can be characterized by comparison of the UV/Vis spectra with those of related systems, which have only one or two extended arms [36].

**Scheme 1:**
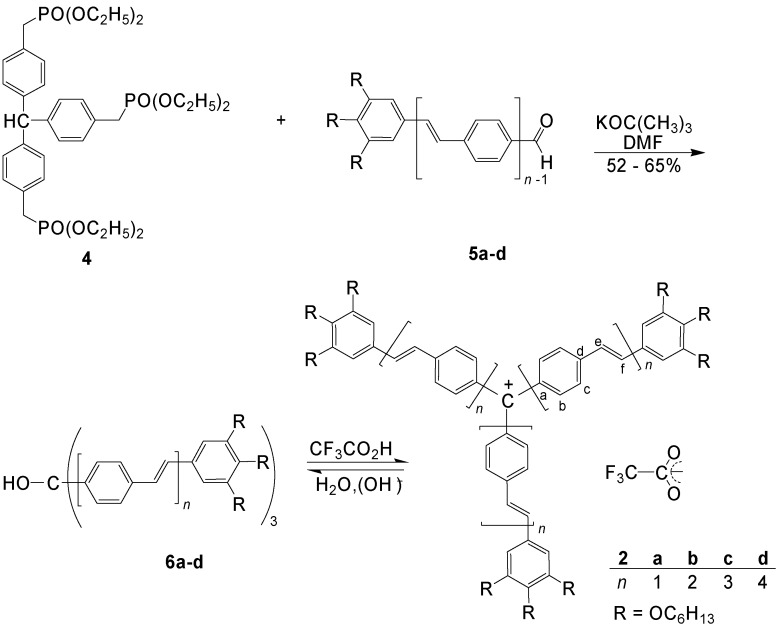
Preparation of methylium salts **2a-d** with three oligo(1,4-phenylene-vinylene) [OPV] arms.

A detailed study of the conjugation effect in **2** with extended arms (*n* = 1-4) was made by Meier *et al*. [41,42]. The carbinols **6a-d** were prepared by Wittig-Horner reactions of the triphosphonate **4** and the aldehydes **5a-d** and spontaneous autoxidation at the central carbon atom. Treatment of **6a-d** with CF_3_COOH yielded the methylium salts **2a-d** (Scheme 1).

**Figure 6:**
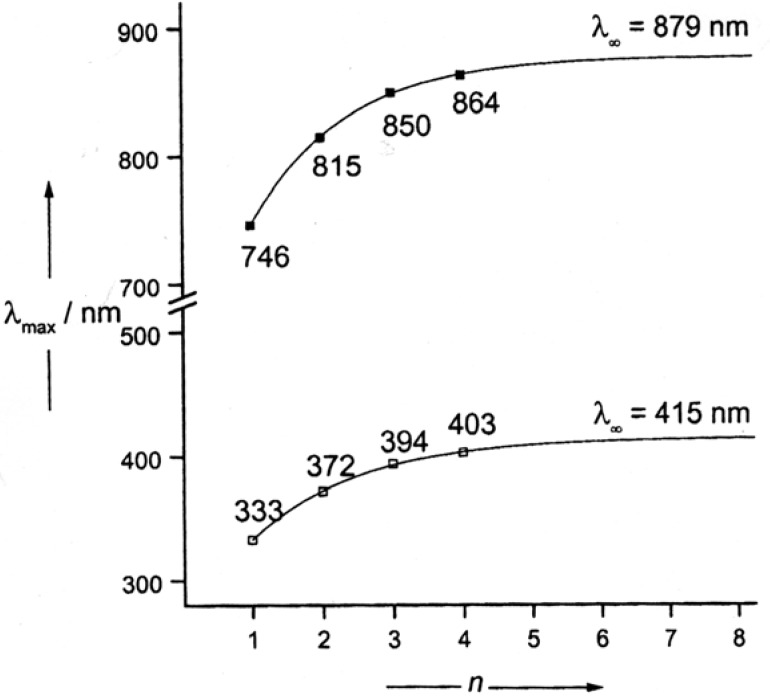
Maxima of the long wavelength absorption of the carbinols **6a-d** (□, measured in CHCl_3_) and their salts **2a-d** (■, measured in CHCl_3_/CF_3_CO_2_H, 7:3) [41].

The increasing length of the arms in **2a-d** results in an increasing bathochromic shift of the absorption from the Vis into the NIR region. According to equation 2, a convergence limit of *λ*_∞_ = 879 nm was calculated (Figure 6).

The delocalization of the positive charge in **2a-d** can be registered by down-field shifts Δ*δ* of the ^13^C NMR signals. A comparison of **2a-d** with **6a-d** reveals, that the central carbon atom has a Δ*δ* value of about 109.9 ± 1.1, whereas the carbon atoms b, d and f (Scheme 1) are shifted by 12.5 ± 0.4, 14.2 ± 0.5 and 9.1 ± 1.1 ppm, respectively [41].

**Scheme 2:**
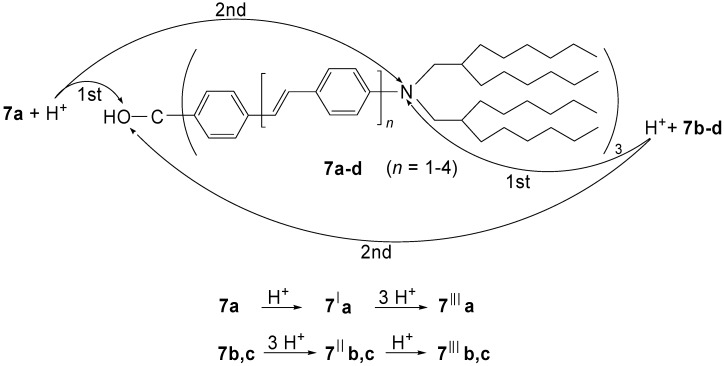
Generation-dependent protonation of the carbinols **7a-d** with trifluoroacetic acid.

**Figure 7:**
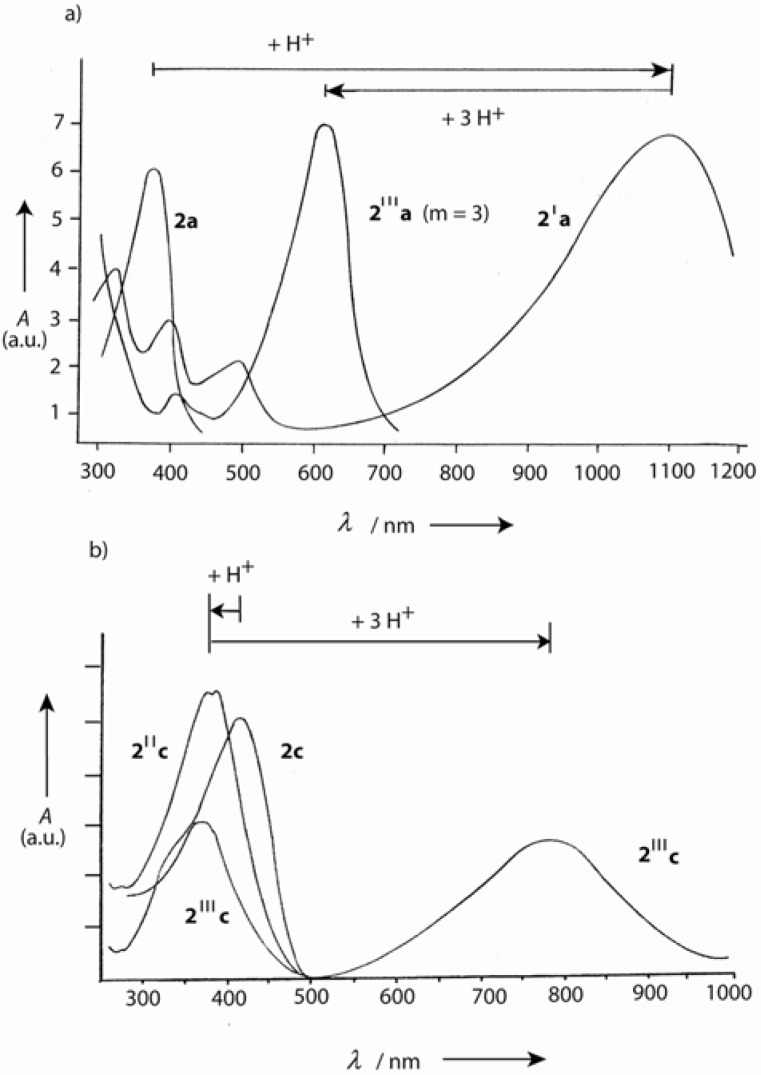
**(**a) UV/Vis/NIR absorption of **7a** in CHCl_3_, with **7′a** as the major component obtained by primary protonation and **7′′′a** obtained as the major component in the final protonation (CHCl_3_/CF_3_COOH). (b) UV/Vis/NIR absorption of **7′c** in CHCl_3_, *N*-protonated carbinol **7″c**, and methylium salt **7′′′c** as major component after the final protonation with CF_3_COOH (molar ratio **7c**/CF_3_COOH, 1:200) [42].

When dialkylamino groups, such as the solubilizing bis(2-hexyloctyl)amino group, are attached in *p*-position of the terminal benzene rings, the situation is more complex (Scheme 2) [42]. The lowest generation **7a** (*n* = 1) forms first by *O*-protonation and elimination of water the corresponding methylium salt **7′a** before the successive three-fold *N*-protonation to **7′′′a** occurs. The higher members **7b,c** (*n* = 2, 3) are first *N*-protonated to the carbinols **7″b,c** before the methylium ions **7′″b,c** are generated. The methylium ion **7′a** is a star-shaped push-pull system with an absorption, which reaches far into the NIR region (*λ*_max_ = 1100 nm). Its extended conjugation causes such an enormous bathochromic shift in comparison to tris(4-dimethylaminophenyl)methylium ions (Crystal Violet: *λ*_max_ = 590 nm). The push-pull effect disappears as soon as (threefold) *N*-protonation occurs. The tetracations **7′′′a-c** have absorption maxima in the visible region (*λ*_max_ = 622, 740 and 790 nm, respectively). They represent a bathochromic oligomer series with a convergence limit of *λ*_∞_ = 827 nm. Figure 7 demonstrates the generation-dependent protonation behavior by the different red- and blue-shifts in the absorption spectra.

Apart from the application as NIR dyes, the methylium salts promise interesting nonlinear optical (NLO) and two-photon absorption (TPA) properties.

### 3.2. [3]Star Systems with Boron Core

**Scheme 3:**
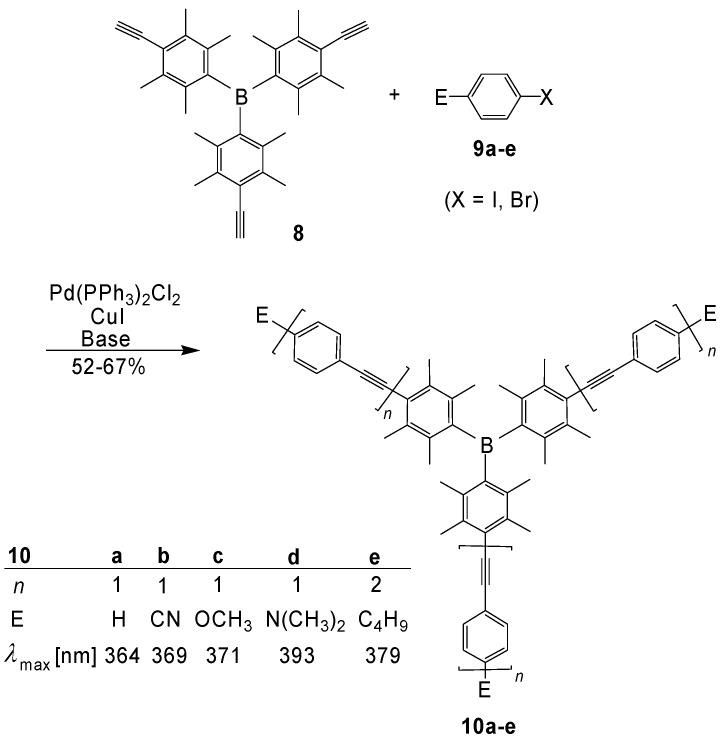
Preparation of the triarylboranes (**10a-e**) with extended conjugation. Absorption maxima in THF.

Planar trigonal boron centers seem to be ideal cores for [3]star oligomers, since the empty p_π_ orbital of the boron atom guarantees an efficient conjugation. This is particularly valid for the p_π_-π* interaction in the LUMO. The resulting low-lying LUMOs cause low reduction potentials and favor therefore *n*-doping and good electron-transporting properties. Moreover, the electropositive character of B should enable an intramolecular charge transfer from electron-rich π-conjugated arms to the center. However, the high sensitivity of boranes toward air and water is normally a big disadvantage for their application. Yamaguchi, Tamao *et al*. [43] overcame this problem by the introduction of durenylene groups, which provoke a kinetic stabilization by steric shielding of the reactive B center. The compounds **10a-e** were obtained by Sonogashira-Hagihara reactions (Scheme 3).

**Figure 8:**
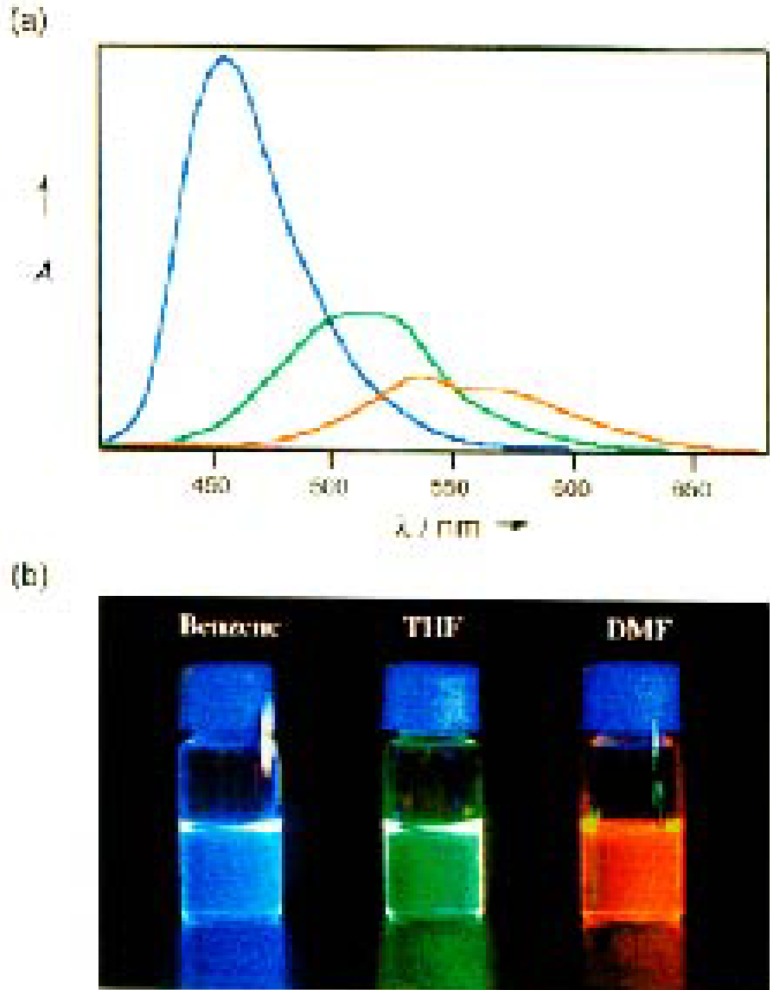
Fluorescence of **10d**: (a) The emission spectra in various solvents (benzene, blue line; THF, green line; DMF, orange line) and (b) a picture of the solutions under irradiation of light at 365 nm. The spectrum in DMF is magnified 10× in intensity [43]. (Copyright 2000, reprinted with permission of Org. Lett.).

The crystal structure analysis [43] of **10a** proved a trigonal planar boron center. The duryl groups are arranged in a propeller-like fashion. Their planes and the central boron plane form dihedral angles of 53-55°. The long wavelength absorption maxima (Scheme 3) are red-shifted by the extension of conjugation. The dimethylamino substituted compound **10d** exhibits a strong push-pull effect with a significant solvatochromism of the fluorescence band. The blue emission color (*λ*_max_ = 457 nm) in benzene changes to green (*λ*_max_ = 512 nm) in THF, and to orange (*λ*_max_ = 530 nm) in DMF (Figure 8). The fluorescence quantum yields of **10a-e** in THF range between *ϕ*_F_ = 0.16 and 0.54 [43].

**Scheme 4:**
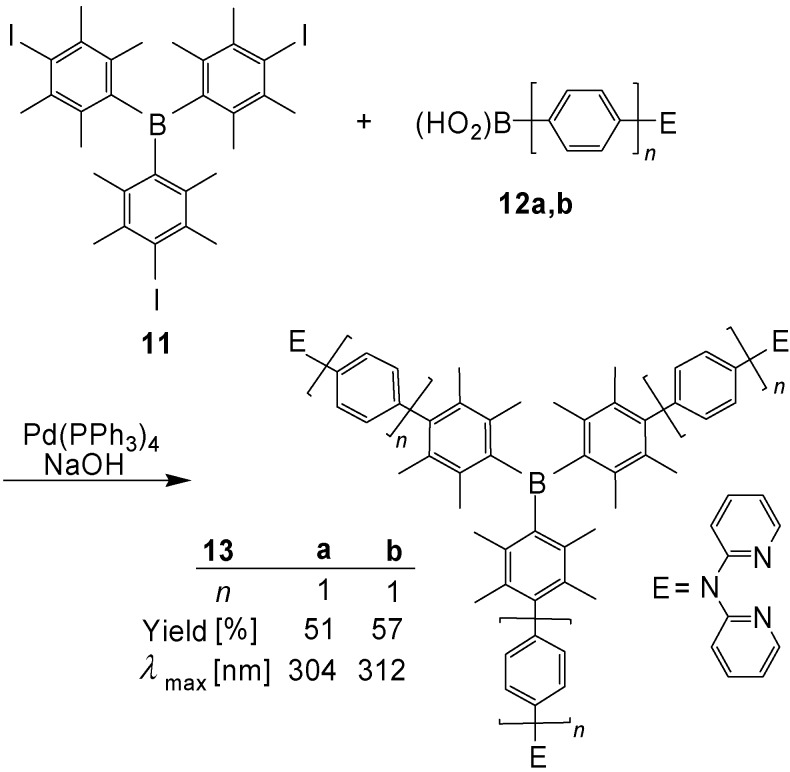
Preparation of the boranes **13a,b** by Suzuki-Miyaura couplings.

Wang *et al*. [44] prepared boranes with 1,4-phenylene repeat units and 2,2′-dipyridylamino end groups, which served for the chelation of metal ions, such as Zn^2+^ (Scheme 4). The final step consisted of a Pd catalyzed Suzuki-Miyaura coupling reaction.

According to the crystal structure analysis of **13b**, the dihedral angle between the inner and the adjacent benzene rings amounts to 74-75°, whereas the dihedral angle between the outer and the middle benzene rings amounts to 38°. The crystals contain pairs of enantiomers, which interconvert rapidly in solution.

In contrast to the absorption bands, the emission bands are solvent dependent and exhibit a remarkably positive solvatochromism. In addition to the blue fluorescence, the compounds **13a,b** show in frozen solution (CH_2_Cl_2_, 77 K) low-energy phosphorescence emissions (*λ*_max_ = 480 nm and 506 nm, respectively). The triplet lifetimes of **13a,b** amount to 9-10 μs. When **13a** is complexed with ZnCl_2_, the fluorescence emission in THF is shifted from *λ*_max_ = 427 to 458 nm. This provides new opportunities for applications, such as fluorescent sensors for metal ions and metal ion containing nonlinear optical materials [44].

Related borates with (1-naphthyl)phenylamino end groups were used as hole transport or hole injection materials in OLEDs [45].

### 3.3. [3]Star Compounds with Nitrogen Core

**Scheme 5:**
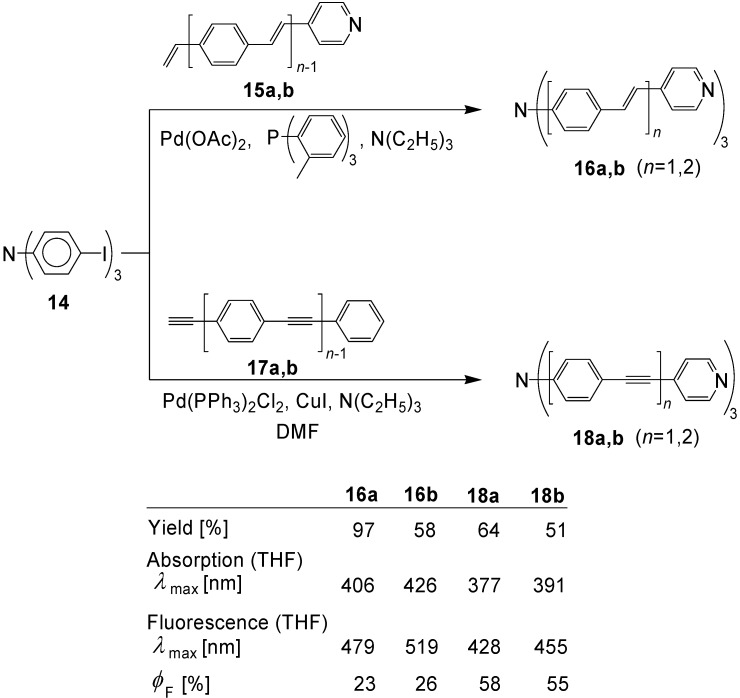
Preparation of tertiary amines having extended conjugation.

Goodson *et al*. [46] investigated push-pull systems with a nitrogen core and 4-pyridyl end groups. Scheme 5 shows the preparation of the compounds **16a,b** (with phenylene-ethenylene repeat units) by applying Heck reactions and **18a,b** (with phenylene-ethynylene repeat units) by applying Sonogashira-Hagihara reactions.

Electron excitation of **16a,b** and **18a,b** leads to intramolecular charge transfer (ICT) states. Increasing conjugation in the three arms shifts the absorption and the fluorescence band to longer wavelengths and increases the two-photon absorption cross-sections. Since *trans*-configured olefinic double bonds have a greater effect than triple bonds, the maximum cross-section (*δ* = 1937 GM) is reached for **16b**. The two-photon absorption (TPA) of **16b** was already studied earlier and **16b** was used as initiator for two-photon polymerization reactions at 830 nm [47].

**Scheme 6:**
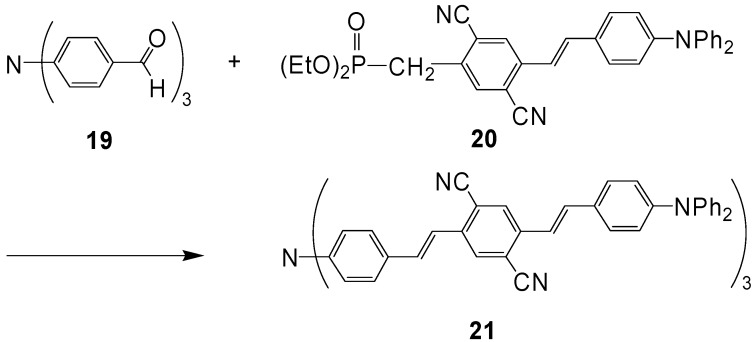
Preparation of tertiary amine **21** with extended conjugation by applying Wittig-Horner reactions.

The related compound **21** was obtained by a Wittig-Horner reaction [48] (Scheme 6) [49].

Dissolved in toluene, compound **21** exhibits a one-photon absorption with a long-wavelength maximum at *λ* = 495 nm and a one-photon emission maximum at 536 nm. The fluorescence quantum yield amounts to 67%. The system has a very high two-photon absorptivity, which amounts to 5030 × 10^-50^ cm^4^ s photon^-1^ (GM) at 840 nm [49]. Related compounds N(OPV)_3_ with cyano groups as end groups or as substituents on the olefinic double bonds were studied by Jeon, Cho *et al*. [50].

Various applications of TPA − such as 3D optical data storage, two-photon laser scanning microscopy, photodynamic therapy, *etc.* − enhanced strongly the demand on new materials with high TPA cross-sections.

**Figure 9:**
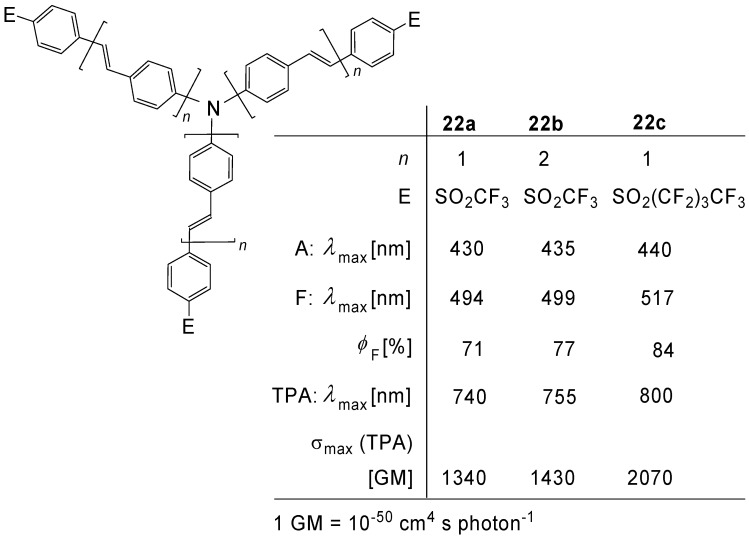
Tertiary amines N(OPV)_3_ with electron-withdrawing SO_2_CF_3_ or SO_2_C_4_F_9_ end groups.

Blanchard-Desce *et al*. [51,52] developed two-photon excited fluorescence (TPEF) probes **22a-c**, which work in the red-NIR region. Triflate or nonaflate groups served as strong electron-withdrawing end groups. The final step in the preparation of **22a-c** consisted of a Heck coupling of tris(4-vinyl-phenyl)amine with the corresponding halogenarenes [51] (Figure 9).

In agreement with time-dependent density functional theory [52], the octupolar fluorophores have an absorbing ground state, which can be understood in terms of Frenkel exciton states, which are delocalized over the three arms, whereas the emitting state is localized on a single arm. The powerful electron-withdrawing end groups provoke a strong dipolar character of the emitting arm, which is expressed by a strong solvatochromic effect [51,52].

**Scheme 7:**
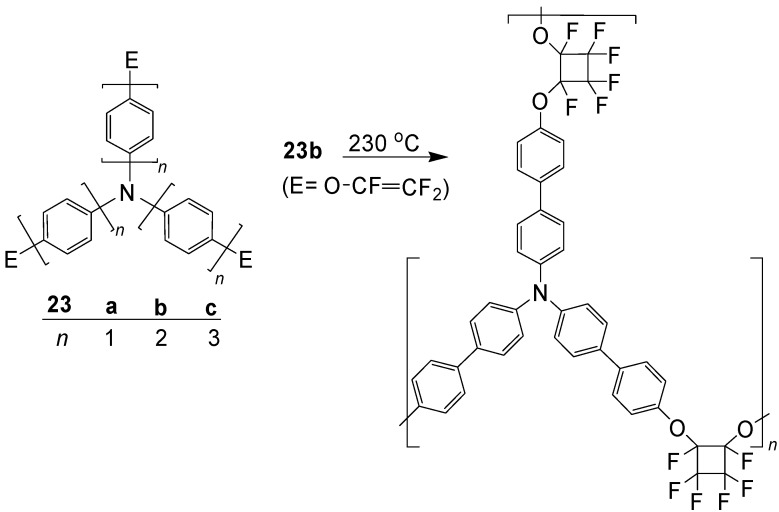
Compounds N(OP)_3_ **23** and cyclopolymerization **23b**→**24b**.

In the series of tertiary amines with three OP chains, many phenyl systems (**23a**, *n* = 1), but few biphenyl (**23b**, *n* = 2) and *p*-terphenyl systems (**23c**, *n* = 3) were reported. All these compounds can be used as hole-transporting materials (Scheme 7).

Kim *et al*. [53] prepared **23b** (*n* = 2) with trifluorethenyloxy end groups and subjected it to a thermal cyclopolymerization. The obtained cross-linked polymer **23b** is thermally and electrochemically stable, solvent resistant, and has a high transparency and a good surface smoothness. A fabricated light emitting diode (PLED) with the configuration ITO/**23b** (30 nm)/ PFO (70 nm)/Ba (15 nm)/ Al (130 nm) had good values for the luminance (*L*_max_ = 1500 cd m^-2^) and the luminance efficiency (*LE*_max_ = 0.132 cd A^-1^, but its turn-on voltage of 7 V is relatively high [53].

A further advantage of such materials in LEDs is due to their emission in the blue or blue-violet spectral range [54,55,56].

## 4. Star Compounds with a Benzene Core

### 4.1. Three-armed Stars (C-4)

#### 4.1.1. Structure and Conjugation

**Figure 10:**
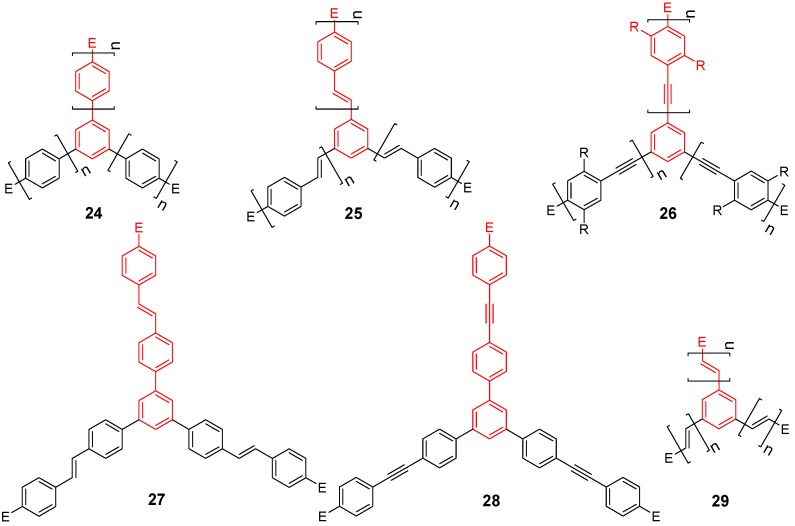
Parent three-arm stars with a 1,3,5-substituted benzene core (C-4).

Figure 10 displays the different reported types of parent conjugated three-armed star scaffolds; C-4-A-3 (**24**), C-4-A-6 (**25**), C-4-A-8 (**26**), C-4-A7 (**27**), C-4-A-9 (**28**) and C-4-A-1 (**29**). The molecular structure of 1,3,5-triarylbenzene **24** is determined by the steric interaction between *ortho* hydrogens of the peripheral ring and the hydrogens of the central benzene. Therefore, such molecules are found to be propeller-shaped, with dihedral angles between 7-49°, depending on the substitution and the packing in the crystal structure [57,58,59]. In contrast, 1,3,5-trisstyrylbenzenes **25** lack such steric interactions and can consequently arrange in a more planar molecular structure in the crystal of the 1,3,5-tris-(3,4,5-trimethoxyphenylethenyl)benzene **25b** [60]. Interestingly, 1,3,5-tris(phenyl-ethynyl)benzenes **26** realize various torsional angles between central and peripheral benzene planes ranging from 0 to 80° in their crystal structures, despite the lack of intramolecular steric interactions [61]. Structures **25** and **26** can establish planar conformations and therefore it can be supposed that the conjugation to the central ring is larger compared with **24**.

Indeed the large difference between absorption and emission spectra of triphenylbenzene **24a** and the stilbene **25a** and tolane **26a** derivatives point already to the reduced conjugation with the central benzene unit in **24a** (Table 2). A more evident sign of the almost missing conjugation may be revealed by the tolane derivative **28a**, with a very small increase in the absorption maximum by only 8 nm compared with the 1,3,5-tris(phenylethynyl)benzene **26a**. Studies of the linear molecules at 77 K point to a larger bathochromic shift of 23 nm when comparable vibrational bands are considered [66], however, this value is still smaller than that expected for the addition of a phenyl group to oligophenylenes (cp. compounds **24b,c**). A second important feature of 1,3,5-trisubstituted benzenes with conjugated arms is their *meta* substitution pattern. *Meta* substitution is known to prevent the conjugation between the individual arms of the star-molecules in their ground state. This is evidenced by the only slightly different absorption maxima compared to the linear derivatives and the threefold higher extinction coefficient (Table 2). Thus, the star-shaped chromophores can be considered as supermolecules consisting of three almost independent arm chromophores including the central ring. Yamaguchi *et al*. argued recently that the bathochromic shift from the absorption maxima of the linear to the branched molecule **26d** would clearly show an increase in conjugation across the central ring. Similar effects can be observed for all chromophores in Table 2. However, such a bathochromic shift can be as well explained by the inductive effect of the additional two conjugated groups at the central benzene ring. Even a single missing methoxy group at the periphery of the arms of molecules **26e-g** give rise to a hypsochromic shift of more than 20 nm compared with **26b-d**.

**Table 2:** Photophysical properties of star-shaped conjugated molecules with a 1,3,5-trissubstituted benzene core.

Compound^a^		Substituents E, R Solvent, *T* (K)	Absorption *λ*_max_ [nm] (*ε* [Lcm^-1^mol^-1^])	Emission *λ_max_* [nm] (Quantum yield *Ф*)	Reference
**24a**	star	H (dioxane)	250 (59,600)	354 (0.10)	[62]
	linear	H (dioxane)	246 (18,600)	316 (0.18)	[62]
**25a**	star	tristyryl (hexane, 293 K)	317 (47,863)	410	[63] [64]
	linear	H (methylpentanes, 295 K) EPA,^c^ 77 K	294 (26,000) 303 (51,500)	347 343	[65] [66]
**26a**	star	H CHCl_3_, 295 K	305 (85,100)	353 (0.15)	[67]
	linear	H CHCl_3_ EPA,^c^ 77 K	300 (24,000) 284 (42,900) 302 (44,500)	— 326	[66]
**27a**	star	H DMF	337 (142,000)	—	[70]
	linear	H DMF	328 (45,200)	—	[70]
**28a**	star	H acetonitril	308 (158,000)	365 (0.92)	[68]
	linear	H EPA,^c^ 77 K	307 (55,000) 325 (51,400)	352	[66]
**24b** n = 2	star	H dioxane	288 (103,000)	364 (0.27)	[62]
	linear	H dioxane	276 (30,200)	342 (0.55)	[62]
**24c** n = 3	star	H dioxane	307 (136,000)	375 (0.71)	[62]
	linear	H dioxane	294 (48,600)	369 (0.71)	[62]
**25c** n = 0	star	Trisdodecyloxy-phenylethenyl CHCl_3_, rt	331 (81,000)	426^b^	[69]
**25d** n = 1	star	Trisdodecyloxy-phenylethenyl CHCl_3_, rt	376 (170,000)	447^b^	[69]
	linear	Trisdodecyloxy-phenylethenyl,R = Methyl	366 (64,000)		[70]
**25e** n = 2	star	Trisdodecyloxy-phenylethenyl CHCl_3_, rt	397 (313,000)	-	[69]
	linear	Trisdodecyloxy-phenylethenyl, R = CH_2_OH CH_2_Cl_2_, 298 K	390 (67,000)	468 nm	[71]
**26b** n = 1	star	H, OCH_3_ CHCl_3_, 295 K	334 (64,600)	384 (0.46)	[67]
**26c** n = 2	star	H, OCH3 CHCl_3_, 295 K	380 (128,800)	409 (0.85)	[67]
**26d** n = 3	star	H, OCH3 CHCl_3_, 295 K	426 (195,000)	464 (0.98)	[67]
	linear	H, OCH3 CHCl_3_, 295 K	390 (56,200)	430 (0.81)	[67]
**26e** n = 0	star	2-methoxyphenylethynyl, OCH3 CHCl_3_, 295 K	314 (58,900)	359 (0.24)	[67]
**26f** n = 1	star	2-methoxyphenylethynyl, OCH3 CHCl_3_, 295 K	377 (104,700)	406 (0.83)	[67]
**26g** n = 2	star	2-methoxyphenylethynyl, OCH3 CHCl_3_, 295 K	405 (128,800)	433 (0.97)	[67]
**29a** n = 1	star	3,4-dibutoxy-phenyl	340		[72]
	linear	3,4-dibutoxy-phenyl	325		[72]
**29b** n = 2	star	3,4-dibutoxy-phenyl	360		[72]
	linear	3,4-dibutoxy-phenyl	350		[72]
**29c** n = 3	star	3,4-dibutoxy-phenyl	380		[72]
	linear	3,4-dibutoxy-phenyl	375		[72]

^a^ star refers to the star-shaped molecules as shown in Figure 10; linear refers to the red substructures highlighted in Figure 10, without the additional arms. ^b^ in CH_2_Cl_2_^c^ EPA solvent mixture (diethylether:isopentane:ethanol = 5:5:2).

Nevertheless, there is an influence of the 1,3,5-trisubstitution to photophysics and photochemistry. For the tristyrylbenzene derivative **25a**, a forbidden transition from the ground state S_0_ to the excited state S_1_ has been discovered, which results in a very long average fluorescence lifetime *τ* in the nanosecond range of up to 43.6 ns [64]. The latter is two orders of magnitude larger than that of the linear chromophore stilbene and depends on the solvent, temperature, substitution pattern and the conformers. The latter can be monitored for **25a** in hexane at 50 K, for which the fluorescence decay can only be fitted with two different values *τ* (43.6 ns, 16,4 ns). This long lifetime allows for the formation of dimers (cyclophanes) even at very low concentration [63,73,74].

The influence of the central core appears to decrease the fluorescence quantum yield of almost all small stars compared to their linear counter parts. This influence becomes negligible with increasing size of the arms (Table 2, **26a**-**g**).

The lacking conjugation across the central benzene core is also demonstrated for OPV and oligoethenylene stars, end-capped with redox active ferrocene [75] or with tetrathiafulvalene derivatives [76]. These groups are oxidized at one single potential, thus the oxidized, conjugated species disclose no visible influence on the oxidation of the other groups. Theoretically, Fukutome *et al*. proposed the 1,3,5-trisubstituted benzene derivative as ferromagnetic coupling unit for polaronic high spin compounds [77]. Dougherty *et al*. could indeed confirm a high-spin system for a doped polymer consisting of oligoenes 1,3-*meta* substituted to benzene centers [78]. However, studies of stilbenoid star derivatives such as **25d** (cp. Table 3, n = 2) revealed that although the first oxidation or reduction produces a paramagnetic radical anion or cation (polaron) the second reduction or oxidation step results always in a diamagnetic dianion [69,79]. Obviously, a diamagnetic bipolaron is always more stable within a stilbene scaffold **25** than two polarons in the triplet state. Ferromagnetic coupling might be more successful in compounds like **27** in which the conjugation to the centre is prevented. This has been demonstrated for a two-arm derivative exhibiting a triplet state for the double charged compound [80].

#### 4.1.2. Synthesis

**Scheme 8:**
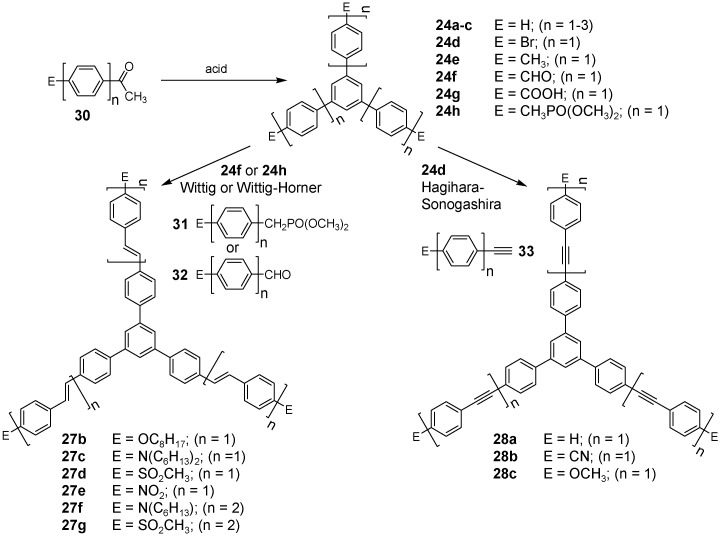
Preparation of three-arm oligophenylene stars **24** with a benzene core and the related oligo(phenylenevinylene) and oligo(phenylene ethynylene) stars **27**, **28** based on a triphenylbenzene core.

Stars with oligophenylene arms A-3 can be prepared by an acid catalyzed cyclization of acetophenones **30** (Scheme 8) [58,81]. Functional groups such as Br, CHO, CH_3_ or phosphonate CH_2_PO(OC_2_H_5_)_2_ allow a further conversion. For example, Suzuki-Miyaura reaction of 1,3,5-tris(4-iodophenyl)benzene with boronic acid substituted oligophenylene building blocks result in the elongation of the scaffold [82]. Wittig-Horner reaction of aldehyde **24f** with phosphonate **31** or phosphonate **24h** with aldehydes **32** afforded stars with OPV arms and a 1,3,5-triphenylbenzene core **27** [83]. Hagihara-Sonogashira reaction with terminal alkynes **33** produced compounds **28** [58,84,85].

**Scheme 9:**
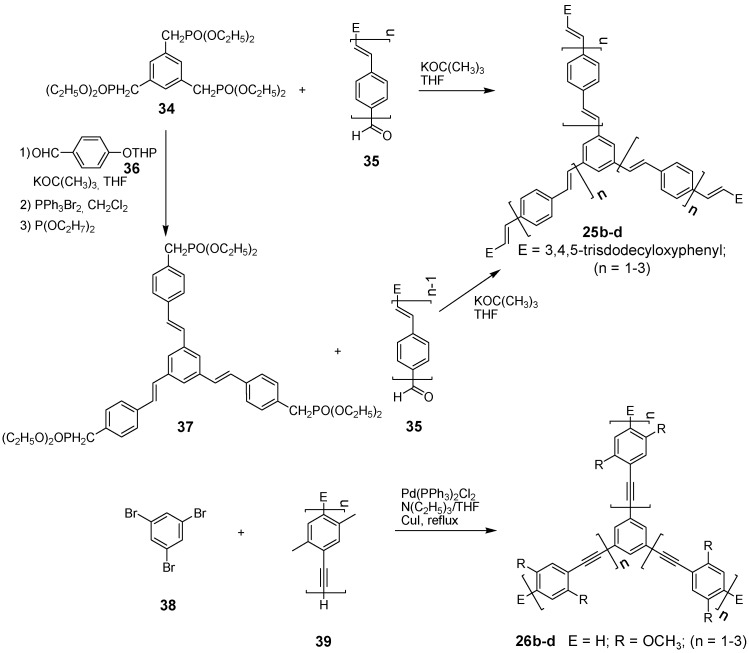
Synthesis of stars **25** with OPV and **26** with oligo(phenylene ethynylene) (OPE) arms.

Stilbene stars with arms A-6 can be prepared by Heck-, Wittig-, Wittig-Horner-, Siegrist-, McMurry- reaction [86] or by the Stille coupling [87]. Commonly the convergent synthesis, coupling the arms with terminal aldehydes to a phosphonate core **34** (Wittig-Horner) afford the materials in excellent yields (Scheme 9) [69,88,89]. Another synthetic route applied a double stage strategy. First, core **34** was elongated in a divergent step using the THP ether **36** and subsequently converted to the phosphonate **37**. In a final convergent step, the three-fold Wittig-Horner reaction affords the target compounds **25** of higher generation. The Hagihara-Sonogashira palladium catalyzed cross-coupling is the most commonly used reaction to assemble three arms with terminal acetylenes **39** in a convergent step to 1,3,5-triiodo- or tribromobenzene **38** to obtain stars **26** with oligo(phenylene ethynylene) arms (A-8) [58,67,90].

**Scheme 10:**
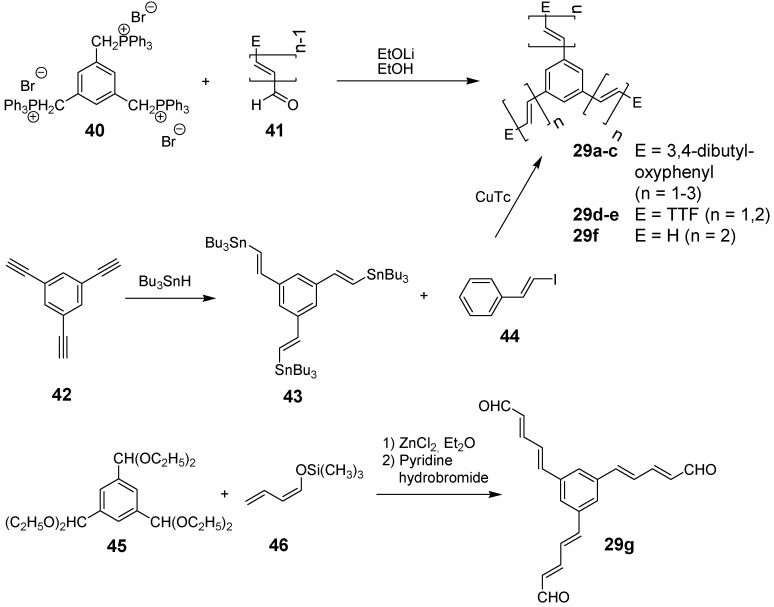
Synthetic routes to stars **29** with oligoethenylene arms. TTF 4‘,5‘-bisdodecylsulfanyltetrathiafulvalene-4-yl; Tc thiophencarboxylate.

**Scheme 11:**
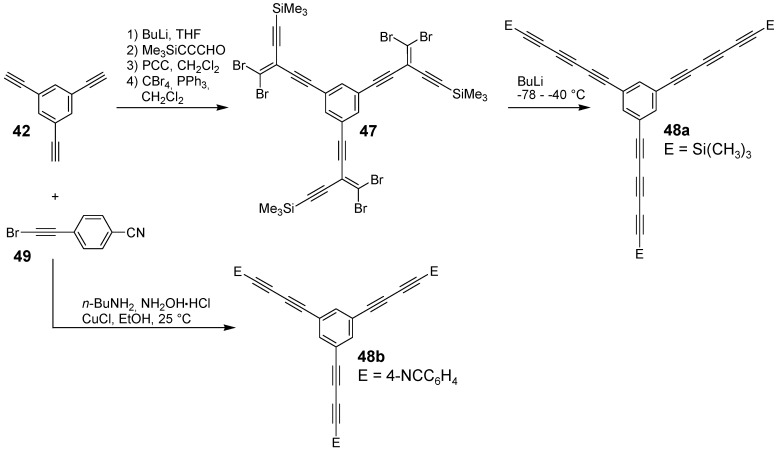
Preparation of stars with oligoethynylene arms.

The synthesis of stars with oligoethenylene arms (A-1) is similar to the one of stilbene derivatives since a threefold Wittig or Wittig-Horner coupling reaction of the oligo(ethenylene)aldehyde-arms **41** with the CH-acidic component **40** delivers the target compounds (Scheme 10) [91,92,93]. Alternatively, the Stille-coupling of **43** with **44** was applied to obtain compounds **29**. A divergent three-step synthesis using butadienoltrimethylsilylether **46** as a key reagent afforded the target compounds in good yields with terminal aldehyde functions **29g** (E = CHO). Acetalization of the aldehydes and repetition of the reaction sequence yielded the elongated compound with four ethenylene groups **29h**. The synthesis of the oligoethynylene stars with arms of type A-2 is somewhat more sophisticated (Scheme 11). Starting from 1,3,5-trisethynylbenzene **42** a star with an uneven number of ethynyl groups can be obtained by a five step synthesis via compound **47** using the Fritsch-Buttenberg-Wiechell rearrangement as final key step to obtain target molecule **48a** [94,95]. A star-shaped diethynylenebenzene derivative **48b** can be prepared in a one step synthesis by the Cadiot-Chodkiewcz reaction [96] converting either the 1,3,5-trisethynylbenzene with a bromoethyne derivative under basic conditions with a copper(I) salt (Scheme 11) [85] or a copper acetylide with a tris(bromoethynyl)benzene [97].

**Figure 11:**
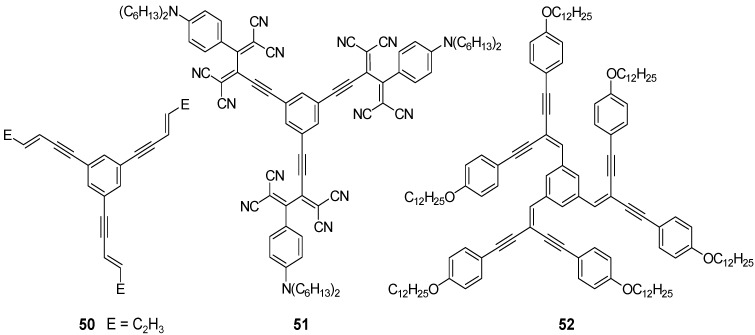
Enyne and ynene stars with a benzene core.

Parent stars with enynes (A-4) or ynenes (A-5) arms are not known. In one case the acidic treatment of an iridacyclopentadiene complex with tris(ethynyl)benzene afforded the vinyl substituted parent enyne **50** (Figure 11) [98]. Cross-conjugated enynes **47** are precursors in the synthesis of oligoynes (Scheme 11) [94,95]. Different cross-conjugated enynes with donor and acceptor groups have been also obtained by a tandem reaction sequence including cycloaddition of tetracyanoethylene to an electron rich triple bond and a subsequent retroelectrocyclization to yield **51**. In the series of ynenes only a branched star **52** is known, obtained by a six-fold Sonogashira-coupling of the acetylene component to tris(2,2-dibromovinyl)benzene [99].

#### 4.1.3. Three-arm Stars with Benzene Centers and Materials Science

*1,3,5-Triphenylbenzene Derivatives (C-4-A-3):* In many star molecules the oligophenylene scaffold were only used as a rigid spacer, because of the limited conjugation along the arms and across the centre [100,101]. For example carboxylate substituted derivatives **24i** (E = COO^-^, n = 2) were applied to produce metal-organic frameworks (MOF‘s) [102], star scaffolds with peripheral metal complexes were investigated with respect to their intramolecular energy transfer [103,104] and stars with peripheral amino acid substituents as biological active materials were synthesized [105]. The parent structures **24a-c** were studied with respect to their potential as laser dyes [62]. The performance of the stars were inferior compared to linear derivatives, however, their potential usage as scintillators have been emphasized. In a further study, oligophenylene (n = 2) stars with acceptor substituents (E = F, CN) have been successfully applied as hole-blocking materials in LED devices based on fluorene/carbazole copolymers with a deep blue emission [106].

*1,3,5-Tris(ethenylphenyl)benzene Derivatives (C4-A-7):*Burn and Samuel *et al*. investigated the potential of compound **27h** (E = 2-(3,5-(di-*tert*-butyl)phenyl)ethenyl) for LED applications [107]. This molecule possesses a high tendency to aggregate and consequently produced a broad emission spectrum. The fabricated LED device, however, was not stable. Brunel *et al*. studied a series of molecules **27b-g** (see Scheme 8) with donor or acceptor substituents E [83,108]. They observed a strong solvatochromic effect upon increasing the solvent polarity which revealed a multidimensional intramolecular charge transfer (MICT) between the core and the periphery, increasing with increasing size and the type of substituents. The very large first order hyperpolarisability *β*(0) of up to 510 × 10^-30^ esu (compound **27g**) was also attributed to MICT. The latter together with the high transparency in the visible range make these materials highly interesting for NLO applications.

*1,3,5-Tris(ethynylphenyl)benzene Derivatives (C-4-A-9):* 1,3,5-Tris(4-ethynylphenyl)benzene derivatives are mainly applied as synthetic precursors, templates or building blocks owing to their rigid or branched framework. Thus they were used as precursors for the synthesis of carbonanoparticles [109], precursors for the preparation of phenylquinoxazoline electron transport materials [90], successfully applied as templates for the synthesis of macrocycles [84], as building block for the self-assembly to MOFs [85] and as components for thermosets [110]. However, the very high fluorescence quantum yield of 0.92 for the parent structure **28a** makes such molecules highly attractive for LED applications [68].

*1,3,5-Trisoligo(phenylene ethenylene)benzene Derivatives (C-4-A-6):* A series of 1,3,5-tris(oligo-(phenylenevinylene)benzenes with peripheral 3,4,5-trisalkoxyphenyl and internal 2,5-bisalkyloxyphenyl groups has been prepared to study mesomorphic properties and photochemistry in liquid crystal phases [69,88,89,111,112,113]. Table 3 presents the thermotropic properties of the mesogens. Only molecules with long hexyloxy or dodecyloxy side chains reveal lamellar and columnar liquid crystal phases, driven by nanosegregation of aliphatic chains and conjugated scaffold. In the hexagonal columnar phases the column diameters are in accordance with the molecular diameters of the star-shaped mesogens. This is interesting since the empty space between the arms must be filled in the condensed phase [18]. As a consequence, two or more mesogens must stack in a columnar unit with a height of about 4 Å without change of the star conformation. A preliminary model shows that nevertheless the relatively rigid oligo(phenylenevinylene) scaffold can accommodate in a structure with an appropriate density, by slight folding and with only few distances which are smaller than the sum of the van der Waals radii [114]. An unusual mesogen design was realized in nematogens **25i**-**m** [112,113]. The aliphatic chains are incorporated in the internal structure of the stilben compounds. Rather shape anisotropy than nanosegregation dominates the mesomorphic behavior, resulting only nematic mesophases.

The photochemistry of peripheral substituted mesogens **25b-h** was studied in thin spin coated films and showed that in the glassy or liquid crystal phase almost all double bonds were consumed. A comprehensive study in liquid crystal cells revealed that in contrast to the photochemistry of 1,3,5-trisstyrylbenzenes in solution, the polymerization reaction of long lived radicals is the dominating reaction path resulting in polymethine structures [115]. Irradiation in the crystal did not result in a photoreaction; only the mobility of the molecules in the LC phase allows the close approach of the olefinic reaction centers [115]. Such photoreactions alter the mesomorphic properties and thus can be applied as imaging technique [74]. Highly interesting supramolecular properties of an amphiphilic trisstyrylbenzene derivative **25n** have been shown in water solution. The critical micelle concentration amounts to 3.17 mgL^-1^. The lower critical solution temperature (LCST) is 32.2 °C at a concentration of 15.5 mM. Above the latter temperature the solution becomes turbid due to micelle formation, whereas at lower temperature the solution is optically isotropic. The stimuli responsive material reveals also consistent changes of the absorption and emission spectra upon micelle formation [116].

Alkylsulfanyl end-capped OPV stars have been prepared as candidates for elements in future nanocircuits [117]. Materials with peripheral ferrocene groups show no significant interaction of the metal centers upon oxidation, as it would be expected for the *meta* substitution at the core [75]. Donor-acceptor substituted star-shaped OPVs **25 o-r**, which are octupolar molecules, are highly attractive as NLO materials in the crystalline solid (Figure 13). The first hyperpolarizbilities *β* of the star molecules are superior over the values of their linear counterparts. The stilbenoid stars show larger values compared to the related tolane systems discussed in the next section, however, the maximum value of *β*(0) = 89 × 10^-30^ esu for **25q** is smaller than that of OPV compound **27g**, were the conjugation to the benzene core is limited [118].

**Table 3:** Thermotropic properties of stilbenoid stars.

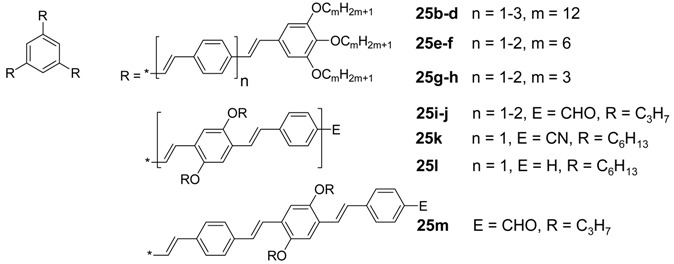		
Compound	Thermotropic behavior (T [°C] / ΔH [kJ/mol]^a^	Ref.
25b (n = 1)	Cr 38/39 Col_hd_ 75/10 I	[88,111]
25c (n = 2)	g 21 (T_g_) Col_hd_ 108/4 I	[89]
25d (n = 3)	g 55 (T_g_) Col_hd_ 199/3 I	[69]
25e (n = 1)	g -15 (Tg) Col_hd_ 74/4 I	[111]
25f (n = 2)	g 21 (Tg) L_D_ 129/3 I	[89]
25g (n = 1)	Cr 189/45 I	[111]
25h (n = 2)	Cr 216/40 I	[89]
25i (n = 1)	g 140 (T_g_) N_D_ 260 I^b^	[113]
25j (n = 2)	g 246 (T_g_) N_D_ 296 I^b^	[113]
25k (n = 1)	Cr 209/40 N_D_ 232/1 I	[112]
25l (n = 1)	g 2 (T_g_) N_D_ 114/0.2 N_D’_ 126/0.4 I	[112]
25m	g 140 (T_g_) N 226 I^b^	[113]

^a^ Values given for the second heating cycle at a heating rate of 10 °C/min. ^b^ data obtained from polarized optical microscopy; T_g_ are approximate values, determined at temperatures when the phase was not anymore shearable.

**Figure 12:**
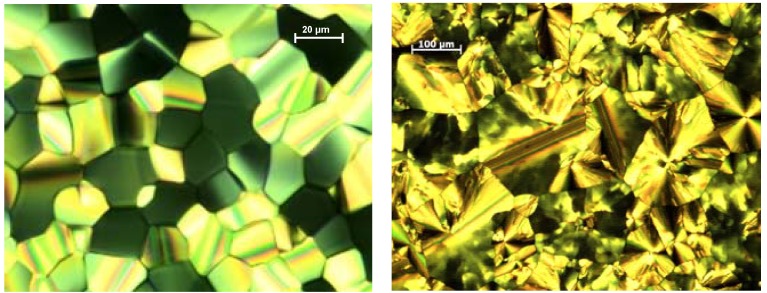
Textures of liquid crystal phases between crossed polarizers. Left: Mosaic texture of compound **25e** at 99 °C typically observed for columnar phases. Right: Texture of discotic lamellar phase of **25f** at 88 °C.

**Figure 13:**
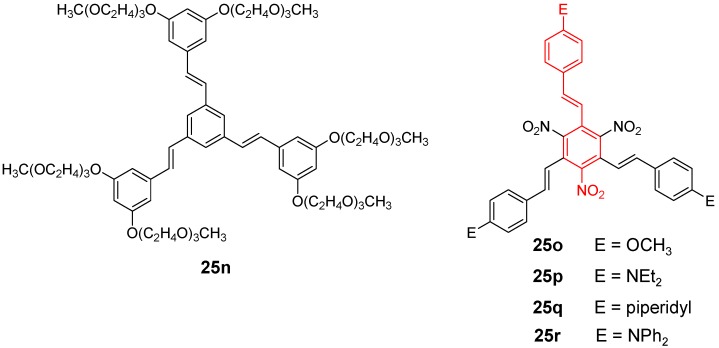
Compounds **25n** and **25o**-**r**. The structure of the linear counterpart, whose properties are investigated for comparison, is highlighted as red substructure in the star molecules.

*1,3,5-Tris(oligo(phenylene ethynylene))benzene Derivatives (C-4-A-8)*: The rigid scaffold of the 1,3,5-tris(oligo(phenylene ethynylene))benzene derivatives plays an important role in materials science. These structures were consequently used as spacers in model compounds for the distance measurements between spins in a defined geometry [119,120,121]. They find applications as building blocks for MOFs [85,122,123] and pure organic supramolecular networks [61,124]. They were also used to prepare new large cyclophynes which are cage compounds apt as new supramolecular hosts [125]. Porphyrine end-capped scaffolds were investigated as rigid guest molecules for porphyrine hexamer macrocycles with high binding constants [126]. Acetylsulfanyl end-capped stars were employed to assemble three gold or silver nanoparticles around the conjugated core with defined distances [127]. Defined structures are also required in nanotechnology. Tour *et al*. prepared carborane end-capped 1,3,5-tris(oligo(phenylene ethynylene))benzenes [129]. The carboranes are the wheels at the rigid scaffold to form nanocars which are proposed to show defined motions at smooth surfaces. Tripodal small stars with peripheral amino acids have been synthesized and their complexation with metals has been investigated [130,131]. Such supramolecular interacting molecules with defined structure may be of interest in pharmacology or as chiral catalyst. Phosphine functionalized stars were converted to palladium complexes, however, the multi metal catalyst exhibited only a low performance [132].

**Figure 14:**
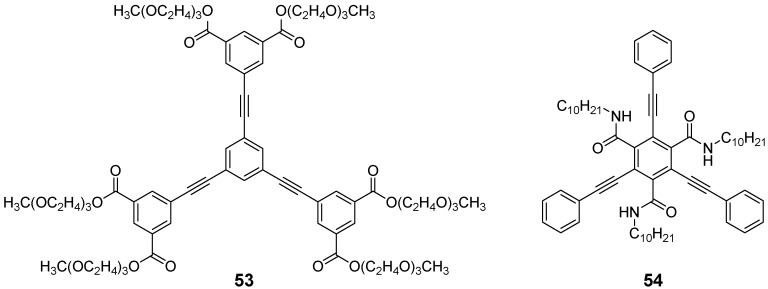
Star-shaped mesogens with a rigid 1,3,5-tris(phenylethenyl) benzene center.

A highly defined shape can be one precondition for the design of liquid crystal materials. In the series of the OPE stars there are only few examples. Moore *et al*. prepared an oligoethylenoxy decorated derivative **53**, which can be regarded as discotic mesogens forming a hexagonal columnar mesophase (Figure 14) [133]. A different crowded mesogen **54** containing the 1,3,5-tris(phenylethynyl) benzene motif and an additional amid functionalization at the core leads to a switchable columnar liquid crystal phase and to columnar aggregation in solution by three hydrogen bonds along the stack [134,135].

**Figure 15:**
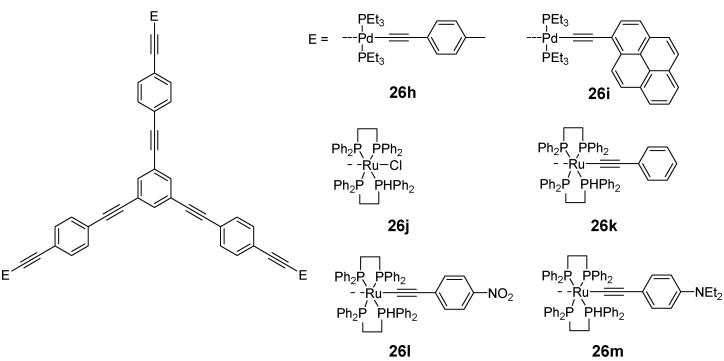
Complex star-shaped supramolecules with a OPE center of Yam *et al*. (**26h**, **26i**) and Humphrey *et al*. (**26j-m**) showing tunable phosphorescence and non linear response.

Especially interesting are the studies of functionalized OPE stars showing electron energy transfers and might be applied as energy traps to mimic photosynthetic light harvesting complexes. Vauthey and Gossauer *et al*. investigated different porphyrine arrays with a OPE star core [136,137]. The similarity of the absorption and emission spectra with the single chromophore suggested that there is only a very weak interaction between the porphyrins. However, the introduction of porphyrins with different metals or without metals revealed a fast energy transfer. With short spacer length the energy transfer occurred via a mixed mechanism-through bond (Dexter) and through space (Förster). With longer spacers the latter became dominant. However recently the same authors highlighted in a further study that with longer spacers there is an efficient energy transfer from the OPE scaffold to the chromophore [138]. This active role of the conjugated scaffold might be promising for the energy transport over longer distances*. N,N*-dimethylaminonaphthalen groups were also attached to a star OPE scaffold [139]. The chromophore absorption and fluorescence dominated the photophysical properties of these molecules. Moreover, the fluorescence quantum yield appeared to decrease with longer OPE arms, which is in contrast to the observations for the parent structures (cp. Table 3). Complex palladium containing star OPEs were presented by Yam *et al*. Figure 15 shows two examples **26h** and **26i**. The palladium assembled the OPE core and in *trans* position a peripheral chromophore via an acetylene linker [140]. The presence of of the heavy metal resulted in an absorption dominated by a metal-to-ligand charge transfer and a predominately intraligand emission from a triplet state. Depending on the LUMO energy levels, the emission could be tuned to originate from the OPE core (e.g., **26h**) or the peripheral chromophore (e.g., **26i**). Fluorescent properties of methoxy substituted OPE stars **26b-d** and **26e-g** of various lengths were studied by Yamaguchi *et al*. (see figure 10 and table 3) [67]. Derivatives with three phenylene ethynylene repeating units reveal excellent fluorescence quantum yields of 0.98 and 0.97 and make such materials extremely attractive for the application in LED devices.

In a different work, redox active fullerenes were attached to the ends of the three arm OPE stars [141,142]. The close resemblance of CV curves for the mutilfullerene-OPEs to C_60_ suggested only a weak electronic communication of the fullerene groups.

Active research on three-armed star OPE is performed in the area of non linear optics. Kondo *et al*. recognized already in 1995 the non-linear response of the parent molecule **26a** in chloroform at 532 nm [143]. Humphrey *et al*. designed a series of ruthenium complexes with a 1,3,5-trisubstituted benzene core (**26j**-**m**, Figure 15) [144]. For these molecules they found only one oxidation wave pointing to non-interacting metal centers. The octupolar stars revealed superior non-linear properties over the related linear dipolar compounds. The first order and the second order hyperpolarisability approached values of *β*(0) = 254 × 10^-30^ esu and |γ| = 9500 × 10^-36^ esu for stars with peripheral phenylethynyl ligands substituted with electron donor or acceptor groups. TPA cross sections of up to 1300 GM were measured for the three arm OPE stars. Interestingly the non-linear response can be tuned electrochemically be oxidation of the metal centers which opens the way for nonlinear electrochromism [144,145]. A similar concept was pursued by Yam *et al*., who incorporated palladium as a metal, however, the TPA cross section were considerably lower, with a maximum value of 32 GM (**26h**) [146]. As mentioned earlier, acetylsulfanyl end-capped OPE stars self-assembled gold or silver nanoparticles in star-shaped arrays. Owing to the plasmon absorption of these nanoparticles these defined materials are highly attractive for the study of non-linear effects. Hupp, Feldheim *et al.* reported extremely high first order hyperpolarisabilities of *β‘* = (3800 ± 410) × 10^-30^ esu, even though the *β* value has been normalized (*β′* = (*β*_particle_^2^/atom)^1/2^) [147].

Tour *et al*. suggested that OPE stars might be also useful as components for molecular electronics, thus they could act as molecular interconnects or molecular field-effect-transistors [148].

**Figure 16:**
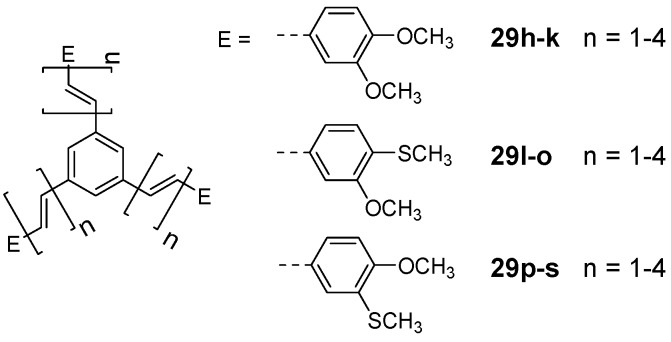
1,3,5-Tris(oligoethenyl)benzene derivatives showing nonlinear optical response.

*1,3,5-Tris(oligoethenyl)benzene Derivatives (C-4-A-1):* Investigation of the electronic interaction of redox centers showed that ferrocene [75,91] and thiafulvalene [76] derivatives do not communicate upon oxidation revealing only a single oxidation and reduction wave. Non-linear optical studies of oligoethenylene derivatives with methoxy and methylsulfanyl substituents at different positions resulted in increasing first order hyperpolarizabilities with increasing size of the conjugated scaffold and reach a maximum of 100.5 × 10^-30^ esu for compound **29o** (n = 4, E = 3-methoxy-4-methylsulfanylphenyl) (Figure 16) [92]. Third order non-linear properties have been studied for similar derivatives with two *n*-butoxy groups (**29a-c**), with second order hyperpolarisabilities γ up to 7.96 × 10^-38^ esu [72,149]. Theoretical studies of the parent 1,3,5-tris(oligoethenyl)benzene suggest that such compounds could be potential wave mixer in coherent ac electronic circuitry [150].

*1,3,5-Tris(oligoethynyl)benzene Derivatives (C-4-A-2):* 1,3,5-Tris(oligoethynyl)benzene derivatives are rarely investigated. They were applied as precursors for metallofullerene synthesis [97] or CT-Chromophores [151,152]. In one example a cyanophenyl capped derivative were employed to produce porous MOFs with silver salts [85].

*1,3,5-Tris(ethenylethynyl)benzene Derivatives or Ethynylethenes (C-4-A-5 or C-4-A-4):* Parent mixed ethenylethynyl or ethynylethenyl derivatives are essentially unknown in materials science. However such structures are apparent as substructures in highly fluorescent materials [99] and CT-chromophores [151,152].

### 4.2. Four-arm Systems-Tetrasubstituted Benzene (C-5)

#### 4.2.1. Structure and Conjugation

**Figure 17:**
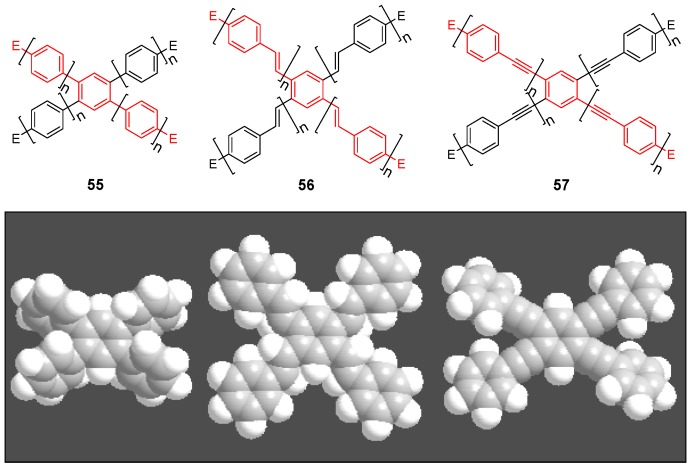
Parent 1,2,4,5-tetrasubstituted benzenes (C-5) with conjugated arms A-3 (**55**), A-6 (**56**) and A-8 (**57**) and their space filling models.

**Figure 18:**
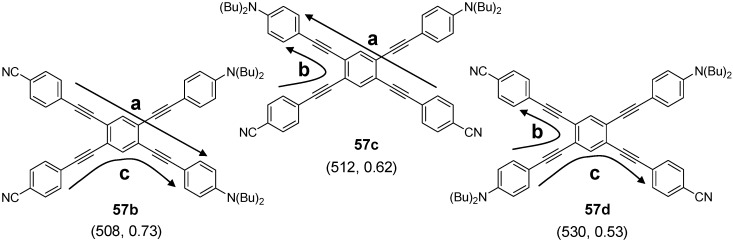
Conjugation paths in donor-acceptor substituted cross-shaped stars **57** (emission maxima [nm], quantum yield *Φ*).

The capability of star-shaped molecules to form planar scaffolds with maximum conjugation is limited by the steric interactions between the arms and the *ortho* located hydrogen atoms of the benzene core or between the arms themselves (see CPK models of Figure 17). The tetra arm derivatives are intermediate between the three arm derivatives with almost no steric interaction between the conjugated arms and the hexasubstituted stars with strong steric interactions. Strongest steric interactions in the series of tetra arm derivatives would be expected for the parent molecule **55a** (n = 1, E = H) [153]. In this case steric repulsion between the *ortho* hydrogens and the interactions between the arms result in relatively large values for the dihedral angles between the core benzene and the peripheral aromatic units in all three polymorphs — one small angle between 39.8°-51.1° and one large angle from 62.2°-66.2°, which are, however, smaller than the values for hexa-substituted benzenes (see chapter 4.3). The steric interaction in the tolane derivative **57a** is reduced to interactions between the arm benzene rings of arm neighbors [143]. The torsional angles disclose to be 11.2° and 36.2°. Different rotamers or conformers impact the conjugation within the two dimensional, cross-shaped molecule and thus also on the photophysical properties. Table 4 collects the optical data for molecules **55b**, **56a** and **57a** and compares it with the data of the linear subunits highlighted in red (see Figure 17). The absorption maxima of the stars **55b, 56a** and **57a** with the strongest oscillator strength are shifted to smaller wavelengths compared with the linear counterparts which points to a reduced conjugation owing to the large dihedral angles. Relatively small differences for **56a** and **57a** would be in agreement with a less twisted scaffold shown in Figure 17. Despite the maxima at shorter wavelengths, compounds **55b**, **56a** and **57a** reveal additional maxima and shoulders at longer wavelengths, which might be attributed to conformers with lower twist and extended conjugation. The fluorescence maxima of all star-shaped molecules are bathochromically shifted compared to their linear derivatives. This points to a low lying S_1_ electronic state compared to the S_1_ state of the linear molecules, which might be rationalized by the participation of a larger fraction of the conjugated molecule to the excited state. Similar results are revealed for many substituted oligophenylene derivatives **55** [154]. In case of compound **56a**, the S_0_ → S_1_ transition is not allowed and consequently, the fluorescence life time is rather long, *i.e.,* in the nanosecond regime [64]. This property may be attributed to the *meta* position of the substituents since even larger life times have been measured in the series of 1,3-distyryl substituted benzene derivatives. Three different charge-transfer pathways have been proposed for a series of donor-acceptor substituted cross-shaped tolane derivatives (Figure 18) in a comprehensive study of photoluminescence and emission after excitation by pulse radiolysis [155,156,157]: The linear (a), the cross-conjugated (b) and the bent (c) ICT pathways. The emission spectra of stars **56b**-**d** with intramolecular charge transfer (ICT) character depend strongly on the substitution pattern and thus on the available charge transfer pathways. Molecules **56b**-**d** possess both two ICT pathways. Molecule **57d** with only bend and cross-conjugated pathways exhibit the maxima at the longest wavelength. Thus it might be speculated that in cross-shaped molecules the linear conjugation across the centre of the molecule is less efficient. The latter is in agreement with the photophysical observations (cp. Table 4).

**Table 4:** Photophysical data of cross-shaped conjugated star compounds.

Compound^a^		Substituents E, R Solvent, T (K)	Absorption λ_max_ [nm] (ε [Lcm^-1^mol^-1^])	Emission λ_max_ [nm] (Quantum yield Ф)	Ref.
				
**55b** (n = 2)	star	H (CHCl_3_)	276 (-)	410	[158]
	linear	H (CHCl_3_)	309 (log4,8)	386 (0.90)	[159]
**56a** (n = 1)	star	H (Toluene, 293 K) (Toluene 77K)	337/370 (S) 350/365(S)/375(S)	450/472 442/463	[64]
	linear	H (Hexane, rt)	350	417	[160]
**57a**	star	H CHCl_3_	315(134,900)/350	391 (0.57)	[143] [159]
	linear	H CHCl_3_	328 (38,900)	348 (0.83)	[159]

^a^ star refers to the star-shaped molecules as shown in Figure 17; linear refers to the red substructures highlighted in Figure 17, without the additional arms.

Structure and conjugation of the cruciform molecules of type **56** and **57** has been theoretically thoroughly studied by energy minimization on the B3LYP/6-311G(d,p) level and natural bond orbital (NBO) analysis [161]. All structures showed a variation in bond lengths on the benzene core, *i.e.,* the bonds between the *ortho*-substituents are calculated to be longer than the four other bonds between the *meta*-substituted positions. These results are confirmed by the molecular structure of **55a** obtained from single crystal analysis of various polymorphs [153]. The NBO analysis was performed on smaller subunits as model systems. In unsubstituted compounds, it was found that the delocalization energy is highest for the linear conjugation pathway (a). However, in donor-acceptor substituted systems this changes in favor for the cross-conjugated pathway (b), which is in agreement with results from fluorescence spectroscopy.

#### 4.2.2. Synthesis

Parent stars with enynes (A-4) or ynenes (A-5) arms are not known. In one case the acidic treatment of an iridacyclopentadiene complex with tris(ethynyl)benzene afforded the vinyl substituted parent enyne **50** (Figure 11) [98]. Cross-conjugated enynes **47** are precursors in the synthesis of oligoynes (Scheme 11) [94,95]. Different cross-conjugated enynes with donor and acceptor groups have been also obtained by a tandem reaction sequence including cycloaddition of tetracyanoethylene to an electron rich triple bond and a subsequent retroelectrocyclization to yield **51**. In the series of ynenes only a branched star **52** is known, obtained by a six-fold Sonogashira-coupling of the acetylene component to tris(2,2-dibromovinyl)benzene [99].

**Figure 19:**
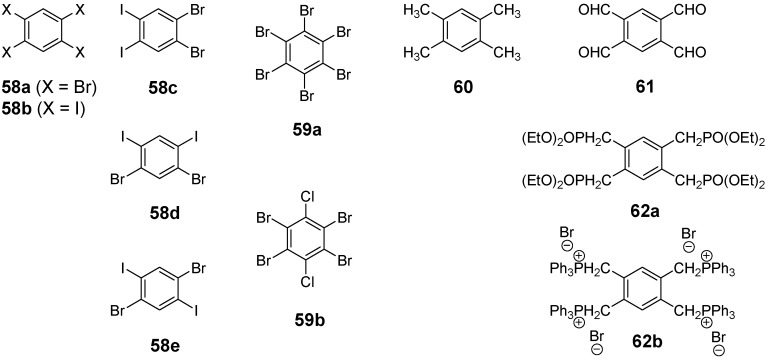
Core building blocks for the synthesis of various tetra-substituted conjugated stars.

The synthesis of the different conjugated tetra-substituted benzene derivatives is performed analogous to the preparation of triarm stars described in section 4.1.2. The most important core reagents **58**-**62** for convergent or divergent synthesis of the target compounds are presented in Figure 19. Convergent synthesis of tetrakis(oligophenylene)benzene derivatives are executed by Suzuki cross coupling of the arms to a tetrabromo center **58a** [154]. Tetrakis(oligo(phenylene ethenylene))benzene and tetrakis(oligo(ethenylene))benzene stars are obtained by Wittig [162,163] or Wittig-Horner reactions [164,165] with cores **61**, **62a**, **62b** or by Siegrist reaction [70] using durene **6**. Tetrakis(oligo(phenylene ethynylene))benzene and tetrakis(oligoethynylene)benzene molecules were prepared by the Hagihara-Sonogashira reaction from core reagents **58a**,**b** [152,166].

**Scheme 12:**
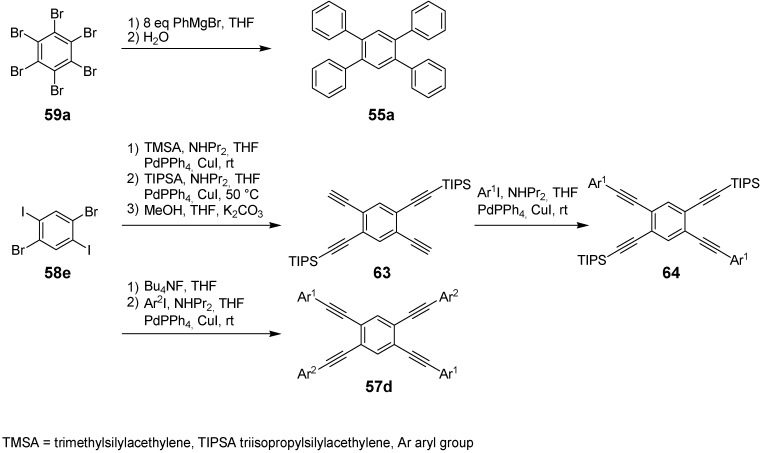
Preparation of tetrakis(oligophenylene)benzenes **55** by an alternative route via an aryne mechanism and the divergent synthetic strategy for the synthesis of stars **57** with different peripheral aryl groups (Ar).

Tetrakisphenylbenzene derivatives can also be obtained by a simple conversion of halogen substituted benzenes with Grignard reagents (Scheme 12) [167]. The resulting tetrakisaryl substituted benzenes are formed by an aryne mechanism in moderate yields.

The nonsymmetric tetrakis(arylethynyl)benzens **57b**-**d** were synthesized by a divergent strategy starting from cores **58a**,**b**,**c** with different activated carbon-halogen bonds [157]. The different introduced silylprotecting groups can be selectively cleaved and converted to terminal alkynes which were coupled subsequently to donor or acceptor substituted aryl iodides.

#### 4.2.3. Four-arm Stars with Benzene Centers and Materials Science

Cross-shaped four arm stars have been studied to a much lesser extent in materials science then three arm (section 4.1) or six arm systems (section 4.3). Materials research focuses on compounds **55**-**57** (Figure 17). 1,2,4,5-Tetrakis(oligoethenyl)benzene derivatives (C-5-A-1) are prepared only as intermediate products to obtain macrocycles [162] or cage compounds [163]. 1,2,4,5-tetrakis(oligoethynyl)benzene derivatives (C-5-A-2) were of interest for the synthesis of graphdiyne substructures [166,168,169], donor-acceptor-functionalized bis(dehydrobenzo[18]annuleno)benzenes [170] and star-shaped compounds [152].

*1,2,4,5-Tetrakis(oligophenylene)benzene Derivatives (C-5-A-3)*: The rigid and shape-persistent structure of tetraphenylbenzene was exploited as building blocks for MOFs (compound **55c (**n = 1, E = SCH_3_), **55d** (n = 1, E = COOH)) [171,172,173,174,175,176] and in crystal engineering by hydrogenbonds (**55e**, n = 1, E = diaminotriazinyl) [177]. For example **55d** complexed to Zn(NO_2_)_2_ was tailored to uptake a remarkable amount of cryogenic hydrogen [174] and could also be postsynthetically modified to adsorb CO_2_ [175]. A framework containing also metalloporphyrin centers was shown to accelerate esterification reactions, thus can be classified as supramolecular catalyst [176].

Much less frequently tetrakis(oligophenylene)benzene materials have been investigated with respect to their opto-electronic properties. Biphenyl derivative **55b** was reported as potential blue light emitting compound [158]. Various substituted derivatives were described as materials for LED applications with improved solubility and processibility compared to linear analogous [154]. Fluorine substituted derivatives have been shown to be excellent hole blocking materials [178]. The latter was rationalized by the large energy gap and the low HOMO energy level.

*1,2,4,5-Tetrakis(oligo(phenylene ethenylene)benzenes (C-5-A-6):* The 1,2,4,5-tetrakis(oligo(phenylene ethenylene))benzenes possess only a limited shape persistence owing to the rather large number of seven possible conformers (c.p. Table 1) [64], thus they were not exploited for MOFs or crystal engineering. However, conformational variation is advantageous for the self-organization into soft materials. A derivative **56** substituted with long oligoethylenoxy chains exhibit analogous to the amphiphilic trisstyrylbenzene **25i** a critical micelle concentration, a lowest critical solution temperature (LCST) and is a stimuli responsive material which reveals consistent changes of the absorption and emission spectra upon micelle formation [116].

**Figure 20:**
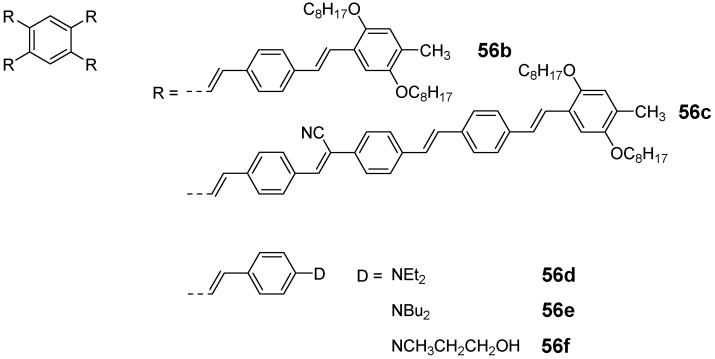
Stilbenoid materials for OLED and TPA applications.

Some studies were dedicated towards the usage of tetraarm stars as LED materials [179,180,181]. Galvin *et al*. reported larger conjugated cross-shaped stars **56b** and **56c** (Figure 20). They are excellent processible and exhibit a large stokes-shift which points to a high electron delocalization in the excited state [181]. Preliminary investigations of mono- and multilayer devices show encouraging data although the efficiencies are rather low. Some additional investigation of the charge transport properties revealed that in these compounds excess positive charges are localized at the alkoxy substituted aromatic rings whereas the negative charges are distributed over the entire star scaffold. According to calculated charge carrier mobility data, these materials are promising.

Theoretical studies suggested that especially cross-shaped stilbenoid stars **56** should reveal high TPA cross sections [182]. The same group reported one year later donor substituted derivatives **56d** and **56e** (Figure 20), with TPA cross sections of up to 1030 GM which are among the highest reported for organic molecular materials [183]. Interestingly, a slight change in the peripheral chains of the molecule reduces this value to only 97 GM for compound **56f** [184]. In a further investigation, Perry, Marder and Rumi *et al*. confirmed the high TPA cross section but pointed out that compared to the linear analogue the TPA is increasing less then with the factor of two [185]. The combination of two crossed linear units in **56**, thus do not efficiently enhance the TPA cross section.

**Figure 21:**
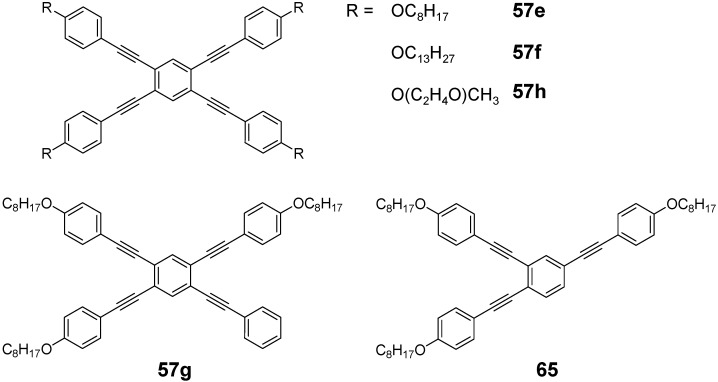
Cruciform nematic mesogens.

*1,2,4,5-Tetrakis(oligo(phenylene ethynylene))benzenes (C-5-A-8):* In contrast, to the stilbenoid compounds **56**, 1,2,4,5-tetrakis(oligo(phenylene ethynylen))benzenes **57** can be classified as shape-persistent compounds and consequently a methylsulfanyl capped derivatives was applied to form 2D and 3D networks with BiBr_3_ [171]. Methylsulfanyl and methylsulfanylethynyl capped derivatives have been also employed to mediate the self-assembly of gold nanoparticles [186]. The scaffold was substituted by amino acid derivatives in order to generate new ligands and pharmaceutical active compounds [130,131]. Although the tetra arm substituted core is reminiscent of a disc only three compounds with liquid crystalline behavior has been published (Figure 21) [187,188]. The symmetric mesogen with octyloxy chains **57e** revealed only a crystalline (Cr 118 °C I), the elongation of the chains (tridecyloxy) lead to the observation of a monotropic nematic phase for **57f** (Cr (74 °C N) 96 °C I) at fast cooling between crossed polarizers. Interestingly, a monotropic phase were detected also for a non-symmetric derivative **57g** (Cr (69 °C N) 84 °C I) and an enantiotropic mesophase were formed only when one complete arm was missing such as in compound **65** (Cr 69 °C N 88 °C I) [187]. Although, the design of cross-shaped mesogens was unsuccessful for the self-assembly in enantiotropic thermotropic mesophases, an amphiphilic derivative decorated with oligoethyleneoxy chains **57h** revealed high association constants of 4.4 × 10^4^-5.2 × 10^5^ M^-1^ in benzene and acetonitrile [189]. These amphiphiles self-organize in unimolecular wires from benzene solution, whereas they form hollow vesicles or 3D toroidal objects in polar solvents.

**Figure 22:**
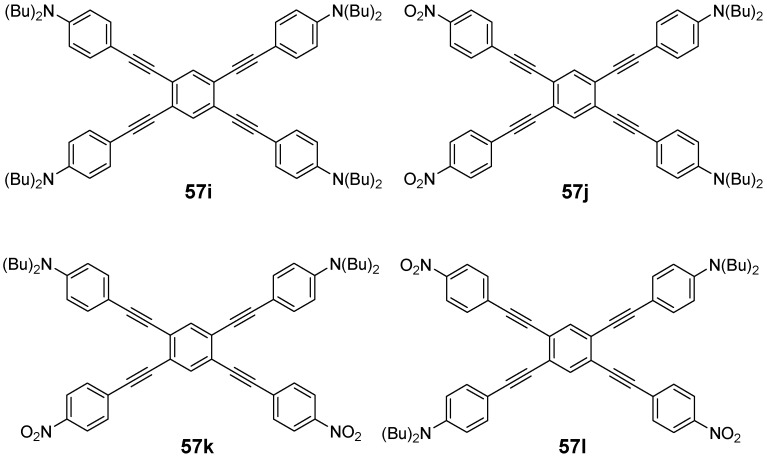
Donor and donor-acceptor substituted cruciform star molecules for optoelectronic and NLO applications.

**Figure 23:**
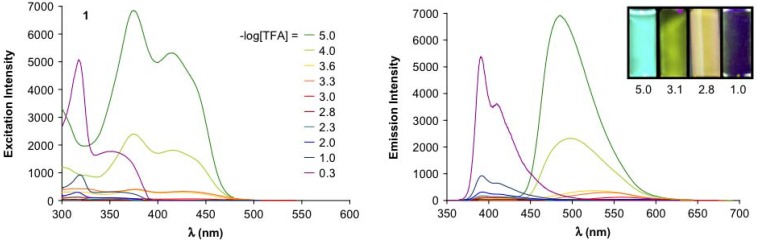
Excitation (left) and emission (right) spectra of TFA titration of **57i** in CH_2_Cl_2_ (ca. 20 mM). Inset: photographs of vials of analyte solutions at indicated -log[TFA] under illumination by high-intensity 365 nm lamp. Reprinted from reference [190] *Tetrahedron*, *64*, Spitler, E. L.; Haley, M. M., Dynamic proton-induced two-stage emission switching in donor-functionalized bis(dehydrobenzo[n]annuleno)benzenes and 1,2,4,5-tetrakis-(phenylethynyl)benzene., 11469-11474, (Copyright 2008, reprinted with permission from Elsevier).

Optoelectronic properties were thoroughly studied for compounds **57a**-**d** and **57i**-**l** (Figure 22). Their relatively high fluorescence quantum yields and the tunability of the emission maximum make these compounds to promising candidates for the application in organic light-emitting diodes [155,156,157]. Recently, the acid titration of **57i** revealed a two-stage emission switching between π-π* and intramolecular charge transfer emissions [190]. The latter is evidenced by a strong bathochromic shift at the beginning dynamic protonation of the first three amino groups. Further increase of the TFA concentration results in the tetra-protonated species revealed by the hypsochromic shift of the emission maximum (Figure 23).

Cruciform conjugated tolane systems are promising for NLO applications as realized already by Kondo *et al*. for the parent molecule **57a** [143]. Recently, the TPA cross section of a donor (**57i**) and a donor-acceptor substituted compound (**57l**) have been measured with maximum values of (520 ± 30) GM at 710 nm for **57i** and (240 ± 20)GM at 750 nm for **57l** in the optical transparent region [191]. Feng *et al*. considered such systems theoretically and proposed highest two-photon responses for *ortho* and *meta* substituted derivatives such as **57j** and **57k**, due to the large dipole moment differences between the ground states and the intermediate states [192]. By incorporation of ruthenium complexes in the arm scaffold, Humphrey *et al*. could obtain cruciform materials with switchable non-linear response by protic and electrochemical stimuli [193].

### 4.3. Six-arm Systems-Hexasubstituted Benzenes (C-6)

#### 4.3.1. Structure and Conjugation of Parent Systems

**Figure 24:**
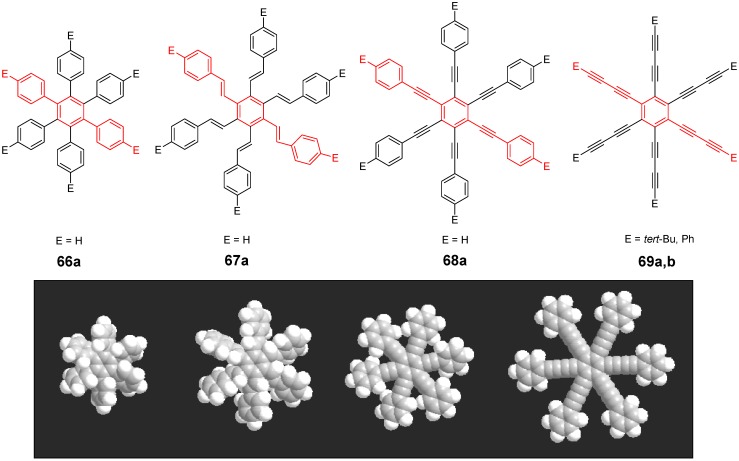
Parent, conjugated, hexasubstituted benzene compounds (C-6).

Hexasubstituted benzenes are predominately steric crowed benzenes. Figure 24 collects four parent structures: hexaphenylbenzene **66a** (C-6-A-3), hexakis(phenylethenyl)benzene **67a** (C-6-A-6) hexakis(phenylethynylene)benzene **68a** (C-6-A-8) and hexakis(phenylbutadiyne)benzene **69b** (C-6-A-2).

The space filling models in Figure 24 demonstrate that for steric reasons the phenyl and the phenylethenyl radicals must turn out of plane relative to the central benzene ring. Only the phenylbutadiynyl derivative may arrange the peripheral benzene rings in a coplanar topology. Single crystals were obtained for derivatives of **66**, **68** and **69**. In the crystal, the molecular structure of hexaphenylbenzene **66a** take a propeller shape with a dihedral angle of 62-71°; in the vapor phase this angle increases to about 90° ± 10° [194,195]. Smaller dihedral angles of 1°-26° were reported for the molecular structure of a hexaphenylethynylbenzene derivative **68b** (E = NO_2_, N(C_6_H_13_)_2_) with pseudo D_3h_ symmetry [195]. No steric interaction becomes evident in the structure of hexa-(tert-butylbutadiynyl)benzene **69a** [196].

**Table 5:** Photophysical properties of hexaarm derivatives.

Compound^a^		Substituents E	Absorption *λ*_max_ [nm]	Emission *λ*_max_ [nm]	References
**66a**	star	H	249	337	[197]
	linear	H	280	342	[159]
**67b**	star	4-dodecyloxy	342	-	[198]
	linear		369	-	
**68a**	star	H	349	449	[143,159]
	linear	H	328	348	[159]
**69c**	star	4-*tert*-butylphenyl	385/415	-	[199]
	linear	phenyl	335/360	-	[200]

^a^ star refers to the star-shaped molecules as shown in Figure 24; linear refers to the red substructures highlighted in Figure 24, without the additional arms.

The conjugation via the centre of the molecule is effected by the magnitude of the dihedral angle between the central and peripheral benzene units. Absorption or emission maxima, reflecting the degree of conjugation, are summarized in Table 5 and compared with the maxima found for linear building blocks. The linear building blocks are defined as the *para* connected segments with maximum conjugation, which Figure 24 highlights in red. The absorption and emission maxima of hexaphenylbenzene **66a** and hexastyrylbenzene **67b** derivatives are shifted hypsochromically compared to the linear oligomers which points clearly to less efficient conjugation. In contrast, star-shaped compounds **68a** and **69c** show bathochromically shifted absorption and emission maxima relative to the maxima of the linear building blocks. Marguet *et al*. predicted qualitatively the absorption spectrum of **68a** by exciton theory and CS-INDO-CIPSI calculations [201]. They proposed that the absorption with the largest transition dipole should be delocalized over the whole chromophore and should therefore be affected by peripheral substituents and the dihedral angle. In contrast, the lowest excited state is localized at the hexaethynylbenzene centre of the molecule and should be insensitive to the peripheral substitution pattern. The wavelengths of the calculated absorption maxima were found to be approximately 100 nm lower than the experimental data, which was only rationalized by solvent effects. Recently it has been demonstrated for compounds with donor or acceptor substituents at the peripheral benzene ring, that absorption and emission maxima are both bathochromically shifted [202]. The largest effect with Δλ > 100 nm was observed for donor-acceptor substituted derivatives with alternately attached three donor and acceptors. The authors rationalize this behavior by the conjugation across the benzene centre. However, the linear donor-acceptor substituted chromophore (E = N(*n*-C_12_H_25_)_2_, CHC(CN)_2_) for which no steric congestion impedes a planar conformation possesses a strongly blue shifted absorption by 38 nm compared with star **68b** (E = N(*n*-C_12_H_25_)_2_, CHC(CN)_2_) [202,203]. The reason for the red-shifted absorption of the hexaphenylethynylbenzene derivatives is not yet fully understood. However, it might be related to interactions of π-orbitals of the arms able to approach each other closely at the centre of the molecule, which was recently also suggested to mediate electronic interactions between oxidized chromophores at a hexaphenylbenzene core [204]. This is supported by the fact that the absorption maxima shifts stepwise to longer wavelengths with increasing number of arms [143].

#### 4.3.2. Synthesis of the Parent Systems

**Scheme 13:**
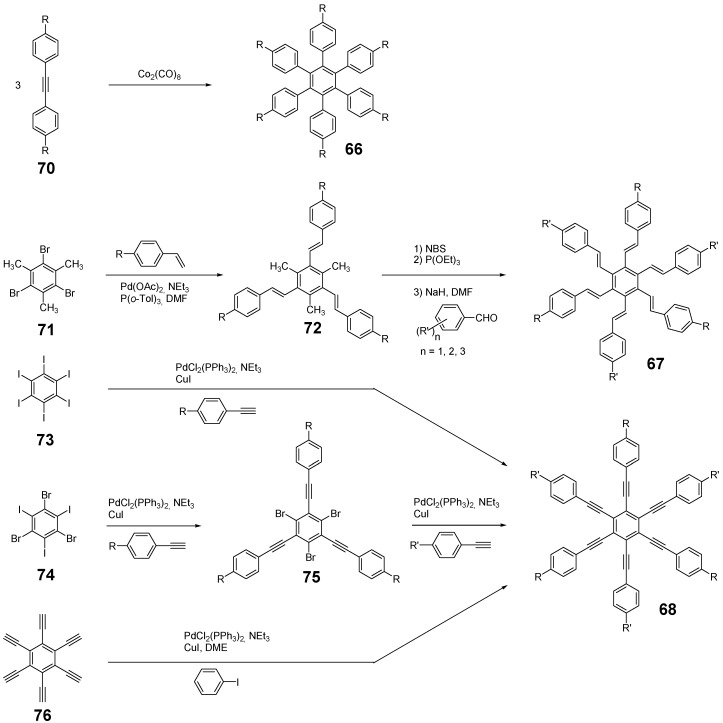
Synthesis of hexaarm derivatives

The synthesis of the parent star-shaped conjugated molecules is summarized in Scheme 13. The *D*_6h_ symmetric hexaphenylbenzene derivatives **66** can be efficiently obtained by the trimerization of diphenylacetylenes **70** with a cobalt or palladium catalyst [205,206]. Early attempts to synthesize and isolate hexaphenylethenylbenzene derivatives by a sixfold Heck reaction failed [207,208]. Efforts to prepare the target compounds by sixfold Suzuki or Stille type cross coupling reactions afforded no or very low yields [208]. Consequently, a two-stage reaction sequence have been developed [209]. In a first step three styryl derivatives were coupled to centre **71** by the Heck procedure. Functional group interconversion of the methyl group in **72** via bromination to a phosphonic acid diethylester allowed in the final step to form a double bond by the Wittig-Horner reaction to yield the target compounds **67** in moderate over-all yields. Compounds **68** (R = R‘) were obtained originally by sixfold Sonogashira-Hagihara cross coupling reactions of core **73** with phenylethynes [207,210,211]. In principle pseudo *D*_3h_ symmetric compounds (R ≠ R‘) can be isolated after a two-stage strategy starting with 1,3,5-Bromo-2,4,6-iodobenzene **74** [202]. However, recently Kuck *et al*. stressed the fact that repetition of earlier sixfold reactions only yielded the pentasubstituted benzene as the principle product [212]. This group could obtain the hexaphenylethynylbenzene only starting from the hexaethynylbenzene core **76**. A sixfold coupling of butadiyne derivatives using modified Sonogashira-Hagihara condition (Pd[P(o-Tol)_3_]_2_, CuI, Et_3_N, NMP) afforded compounds **69** in moderate yields [199].

#### 4.3.3. Hexaarm Stars with Benzene Centers and Materials Science

*Hexaphenylbenzene Derivatives (C-6-A-3):* Many applications of hexaphenylbenzenes and hexa(oligo-*para*-phenylene)benzenes are based on the rigid molecular scaffold in which the conjugation does not play the major role. They are used as synthetic precursors for hexabenzocoronenes and nanographenes [205,213,214,215,216], as oligophenylene dendrimer cores [217], as templates for the preparation of macrocycles [218,219], as star-shaped amphiphiles for the active layer of nanofilters [220,221] and as model scaffolds for natural antenna complexes with a well-defined porphyrine chromophore array [206,222,223]. A rigid scaffold is also a precondition for mesophase formation. Hexaphenyl- and hexabiphenylbenzene were decorated with dodecyl chains or dodecylthienyl groups in order to generate mesomorphic properties. The resulting materials showed complex thermotropic behavior, for which the high temperature phases have been suggested to be columnar mesophases [224].

Hexaphenylbenzene and hexakis(4-*n*-dodecylbiphenylyl)benzene can be reduced to their hexaanions. In these hexaanions the central benzene ring is twisted and undergoes a dynamic processes which could be monitored with NMR investigations for the thermally surprisingly stable biphenylyl system [187].

**Figure 25:**
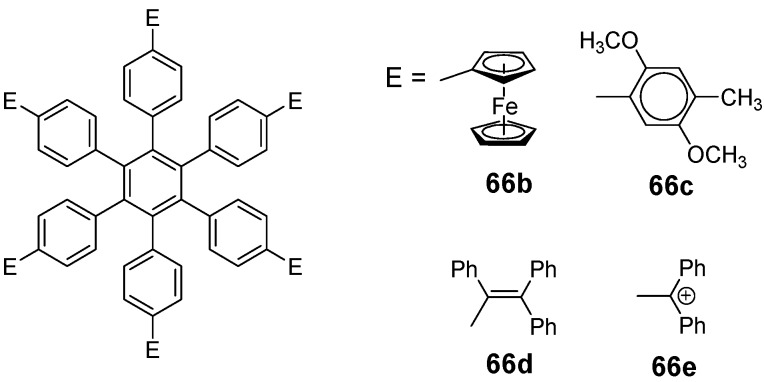
Hexaphenylbenzene derivatives with electrochemically active peripheral groups.

Much work has been undertaken to functionalize hexaphenylbenzene with electrochemical active groups shown in Figure 25.

Compounds **66b** and **66c** can be six-fold oxidized at a single potential which was attributed to the missing conjugation of the redox-active peripheral units E [225,226]. Indeed the molecular structure of **66b** in the single crystal revealed not only the typical dihedral angle of about 66° between peripheral phenyl groups and the central benzene, but also a dihedral angle of 4-31° between ferrocenyl groups and the peripheral benzene units. Star-shaped compound **66c** was called an electron-sponge which has been successfully applied to oxidize aromatic and olefinic compounds to produce their radical cations.

A different situation comes across in compound **66d** [204]. The redox-active tetraphenylethen includes a phenyl group from the propeller core. The absorption spectrum is slightly red-shifted to 324 nm and the extinction coefficient is higher than the six-fold extinction of tetraphenylethen. These observations were attributed to a weak electronic interaction of the arm units and could be confirmed by cyclic voltametry revealing three overlapping oxidation waves. This is based on the rapid charge transfer between the active centers of the molecule in a radical cation which consequently affects the removal of further electrons. In compound **66e** six trityl cations are arranged about a benzene ring [227]. It can be produced by treatment of the alcohol precursor with methyl sulfonic acid. Again a red-shift (20 nm) of the trityl cation absorption compared with the parent trityl cation suggests a weak interaction between the arms of the star-shaped molecule. The compound can be used as a hydride transfer reagent in Organic Chemistry. Müllen *et al*. investigated the photophysical properties of higher oligophenylen star homologues, namely stars with terphenyl and quarterphenyl arms substituted with alkyl chains at the *para*-position [228]. They pointed out that the bathochromic shift of the absorption maxima (303 nm, 316 nm) compared to individual terphenyl (285 nm) or quarterphenyl (299 nm) arms does not account for completely independent chromophores. The materials are highly fluorescent, with high quantum yields, possess a low crystallization tendency, do not form aggregates or excimers and thus are suitable for light-emitting diodes. Donor-acceptor substitution of hexaphenylbenzene makes these stars to NLO materials with high second order polarizabilities [195].

**Figure 26:**
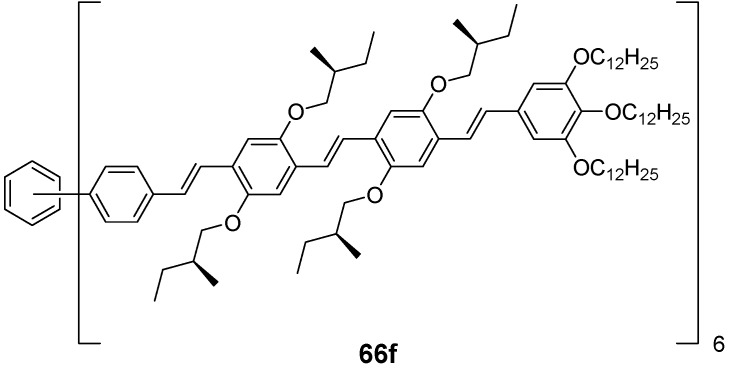
Disc-shaped mesogen self-assembling in helical columns.

*Hexakis(phenylethenyl)- and Hexakis(oligo(phenylene ethenylene)phenyl)benzene Derivatives (C-6-A-6 and C-6-A-7):* Hexakis(phenylethenyl)benzenes **67** have been synthesized due to expected interesting intramolecular or intermolecular photochemical reactions. However, only an unspecific photopolymerization has been observed [198]. Surprisingly, the attachment of long, peripheral alkoxychains does not result in liquid crystalline materials. Meijer and Schenning *et al*. synthesized a star with six arms based on a oligo(phenylene ethenylene) scaffold **66f** (Figure 26) [229]. They circumvented the demanding synthesis of a hexa(phenylethenylene)benzene centre **67** by the preparation of the star using the simple high yielding trimerization procedure of ethynylene derivatives resulting in a molecule **66f** with hexaphenylbenzene core. Compound **66f** reveals remarkable self-assembly properties. Even at concentrations of 10^-7^ mol/l in heptane at 90 °C they stack into helical columnar aggregates. In bulk the mesogen self-assemble in a soft, columnar crystal. The high order in the aggregates of this conjugated oligomer makes these compounds highly appealing for supramolecular electronics.

*Hexakisoligo(phenylene ethynylene)benzene Derivatives (C-6-A-8):* As mentioned for the hexaphenylbenzene cores, hexa(phenylethynyl)benzene scaffolds are often prepared to utilize their defined and persistent shape. For example, they were employed to design new ligands for multimetal complexes [131,230,231,232,233,234]. Especially the cobalt complexes, were subsequently used to prepare carbon nanomaterials by pyrolysis [235]. The most important property with respect to the shape anisotropy is the self-organization in liquid crystals. The hexa(phenylethynyl)benzene core with peripheral flexible chains is one of the most prominent disk-shaped mesogen, forming exclusively discotic nematic mesophases. Table 6 summarizes the thermotropic behavior of some selected examples. In the series of alkyl (*n*-C_n_H_2n+1_) substituted mesogens the mesophase range increase until n = 7 and decrease with longer chains until it disappears for n = 12 [211,236]. With peripheral alkoxy substituents (OC_n_H_2n+1_) the temperature interval of the nematic mesophases is considerably extended [210,237]. Changing the chain position from *para* to *meta* or *ortho* in the peripheral ring results in the loss of the LC behavior. Although, methyl groups at the *meta*-position lead to a decrease of the melting transitions, almost room temperature stable discotic nematic liquid crystals have been obtained only by desymmetrization of the star mesogen [237]. Such nematic phases are attractive for the application in wide viewing angle nematic displays [238].

**Figure 27:**
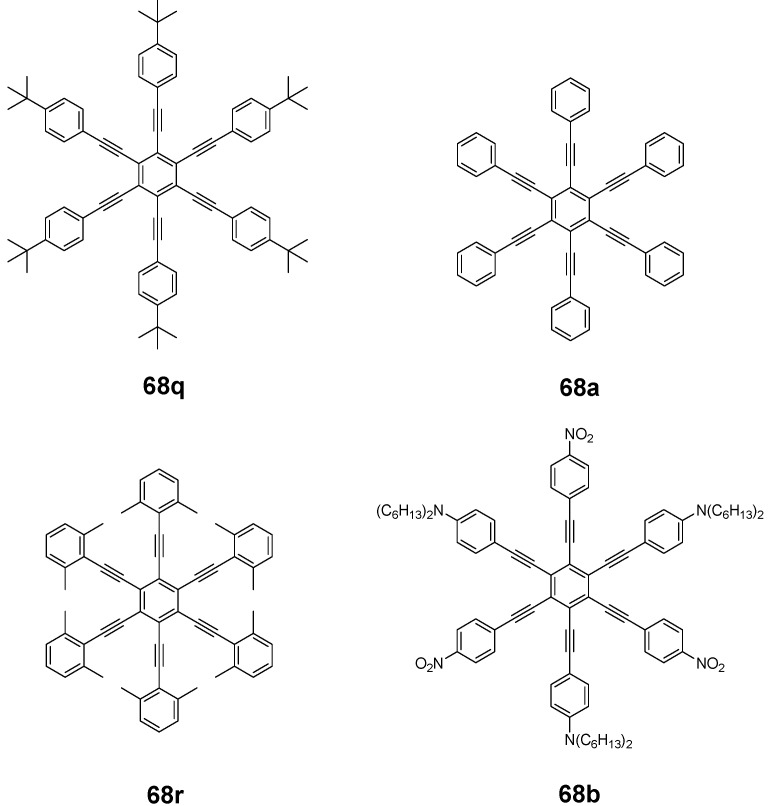
Hexa(phenylethynyl)benzene stars for NLO or two-photon absorption applications.

For photophysical applications, not only the shape but also the conjugation paths are important. In a fundamental study, Kondo *et al*. reported a rather large third order non-linearity for the non-polar parent compound **66a** [143]. A substantial second order polarizability of *β*_zzz_ = (1670 ± 140)×10^-50^ Cm^3^V^-2^ have been recorded for the donor-acceptor substituted hexa(phenylethynyl)benzene **66b** [195]. The excellent 2D NLO property have been attributed to the conjugation across the central benzene ring, since related more twisted derivatives exhibit significantly reduced values. Similar relationships have been found when the two-photon absorption properties were studied for compounds **68a**,**q**,**r** (Figure 27) [239]. The value of the cross section decreased in the series of **68q** > **68a** > **68r**. The larger value for **68q** was rationalized by the substitution effect of the donation *tert*-butyl groups. The lowest value for **68r** can be explained by the reduced conjugation across the centre of the molecule due to steric interactions of the methyl groups in meta-position [240].

**Table 6:** Hexaarm systems with a benzene core and phenylethynyl arms.

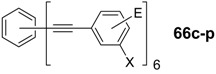					
E^a^	X	Transition Temperatures /°C^b^	E^a^	X	Transition Temperatures /°C^b^
n-pentyl	H	Cr 170 N_D_ 185 I	hexyloxy	H	Cr 144 N_D_ 216 I
n-hexyl	H	Cr 124 N_D_ 142 I	heptyloxy	H	Cr 109 N_D_ 193 I
n-heptyl	H	Cr 98 N_D_ 131 I	*meta* hexyloxy	H	Cr 87 I
n-octyl	H	Cr 80 N_D_ 96 I	*ortho* hexyloxy	H	Cr 63 I
n-nonyl	H	Cr 67 N_D_ 83 I	octyloxy	CH_3_	Cr 95 N_D_ 176 I
n-decyl	H	Cr 71 (N_D_ 54) I	3,7-dimethyl-octyloxy	H	Cr 80 N_D_ 124 I
n-dodecyl	H	no LC	3,7-dimethyl-octyloxy	CH_3_	Cr 71 N_D_ 147-160 I

^a^ Substituents E are always in *para*-positions if not otherwise stated. ^b^ Cr crystal; N_D_ discotic nematic; I isotropic.

## 5. Compounds with Heterocyclic Cores

### 5.1. Pyridine-based Stars (C-7-A-6, C-7-A-7):

A multitude of 2,4,6-triarylpyridines (C-7-A-3) with a large variety of additional substituents on central and the peripheral rings as well as conjugated π-systems incorporating pyridine rings has been prepared in the past. However, only a few star-shaped compounds with larger conjugated branches in the 2,4,6-positions appeared in the literature. Compared to benzene as a core, the pyridine offers a higher electron affinity [241], a subtle difference between substituents in the 2,6- and 4-positions, and the ability to be protonated or quaternized. Nevertheless, 2,4,6-trisubstituted pyridines can be regarded as pseudo-*C*_3_-smmetric.

2,4,6-Trimethylpyridine (*sym*-collidine) **77a** (R^4^ = H) is the most prominent starting material for stars with a pyridine core. The base-catalyzed condensation of *sym*-collidine with 4-, 3,4-di- and 3,4,5-tridodecyloxy-substituted *N*-phenylbenzaldimines **78a**-**c** (Siegrist reaction) gives 2,4,6-tristyrylpyridines **79a**-**c** in moderate to good yields and with an extraordinary high *E*-selectivity (Scheme XIV). Contrary to similar compounds with a benzene core, these stars do not form mesophases [242]. The analogous compound **79d** with a 3,5-didodecyloxy substitution was prepared by Attias [243]. Substituted 2,4,6-tristyrylpyridines, e.g., the *p*-chloro derivative, have been claimed as electroluminescent materials [244].

**Scheme 14:**
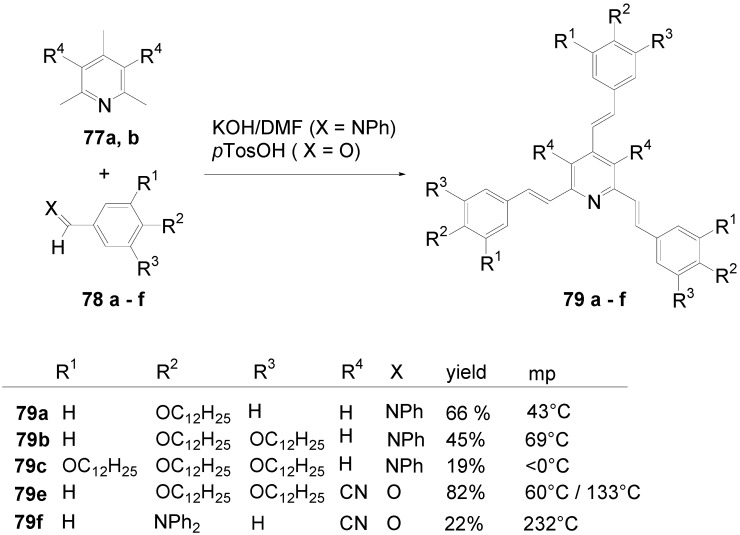
Siegrist reaction of 2,4,6-collidine **77** with alkoxybenzalanilines **78**.

The acid-catalyzed condensation of 3,4-didecyloxybenzaldehyde **78e** with 3,5-dicyanocollidine **77b** (R^4^ = CN) gave a yellow-orange tristyrylpyridine **79e** in 42% yield [245,246].

In dichloromethane solution, the absorption maximum is observed at *λ* = 400 nm (*ε* > 80,000 Lmol^-1^cm^-1^) and, separated by a large Stokes shift, a fluorescence maximum at *λ*^F^ = 550 nm (*Φ* = 0.40). A reversible reduction was observed by cyclic voltammetry at-1.19 V (*vs.* SCE) and an irreversible oxidation wave at 1.28 V. The electron affinity could be calculated to be about 3.6 eV.

Between octadecylsilane treated surfaces **79e** shows a fan-shapes texture, characteristic of a hexagonal columnar mesophase that exists between 81 °C and 133 °C (DSC). According to the X-ray diffraction of the mesophase, columns composed of discs with an antiparallel orientation form a hexagonal lattice. The anti-orientation of the discs results from dipolar interactions, these are also responsible for the stability of the columnar structure [245,246].

Related stars with diphenylamine end groups **79f** were prepared via piperidine-catalyzed Knoevenagel condensations in 22% to 27% yield [247]. The absorption spectra of these octupolar dyes show maxima between *λ* = 476 and 480 nm and an intense (*Φ* = 51-54%) orange fluorescence with maxima at *λ*^F^_max_ = 575-586 nm. Two-photon absorption cross-sections at 800 nm were found to be 187-204 GM. The TPEF is shifted about Δ*λ* = 6-18 nm to the red relative to the single-photon excited fluorescence. Comparing 2,4-di- and 2,4,6-tristyryl-dyes, the authors found a strong cooperative enhancement of the TPA cross-section among the three branches which may be attributed to an electronic coupling among the core and the three individual branches of the octupolar molecule.

Pyridine stars with larger π-conjugated branches **80** (C-7-A-7-E) have been prepared by Siegrist [248]. Threefold base catalyzed reaction of 2,4,6-tris(4-methylphenyl)pyridine with the Schiff bases of benzaldehydes and aniline **78g, h** (**g**: R^1-3^ = H, **h**: R^1^ = R^3^ = H, R^2^ = C_6_H_5_) gave trisstilbenylpyridine **80a** (E = phenyl) and tris-4´-phenylstilbenylpyridine **80b** (E = 4-biphenylyl) in 41% and 55% yield. The acetic anhydride promoted condensation gives similar yields [249].

The absorption of **80a** has a maximum at *λ* = 342 nm (DMF), a threefold phenyl-substitution in the *p*-positions of the stilbene arms (**80b**) resulted in a red-shift to *λ*_max_ = 360 nm.

**Scheme 15:**
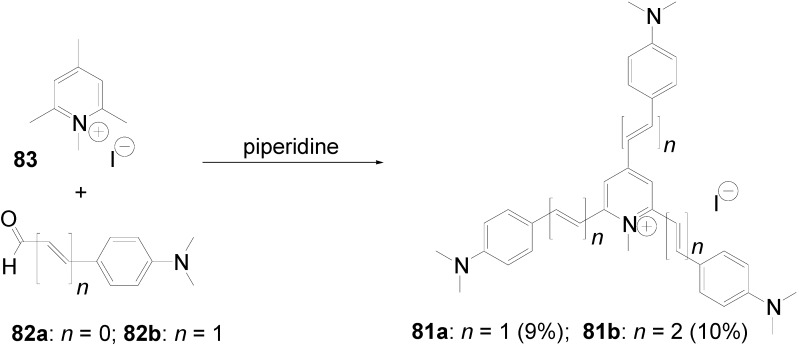
Synthesis of *N*-alkyl-2,4,6-tristyrylpyridinium salts **81**.

Tristyrylpyridinium dyes **81** (Scheme 15) were prepared by piperidine catalyzed condensation of the *N*,*N*-dialkyl-aminobenzaldehydes **82** with a 1,2,4,6-tetramethylpyridinium salt **83** in 10-25% yield [250,251].

Along with the substitution of the pyridinium ring with one, two or three dimethylaminostyryl groups, the absorption maximum is first shifted to the red (mono-α: *λ*_max_ = 478 nm; mono-γ: *λ*_max_ = 502 nm; di-α,α´: *λ*_max_ = 515nm, di-α,γ: *λ*_max_ = 518 nm) but the threefold styryl-substituted dye **81a** absorbs at higher energies (tri-α,γ,α´: *λ*_max_ = 510 nm). In the series with dimethylaminophenylbutadienyl groups, the sequence for *λ*_max_ is mono-α: *λ*_max_ = 508 nm; mono-γ: *λ*_max_ = 525 nm; di-α,α´: *λ*_max_ = 552 nm, di-α,γ: *λ*_max_ = 557 nm and the tri(phenylbutadienyl) dye **81b** shows *λ*_max_ = 559 nm [250]. These dyes are negative solvatochromic, the absorption maxima are shifted about 36 and 30 nm resp. to the blue comparing solutions in CHCl_3_ with those in methanol.

Substituted with six long octadecyl side chains, the absorption maximum of a *N-*methyl-2,4,6-tris(aminostyryl)pyridinium salt **81c** in CHCl_3_ appears at *λ*_max_ = 520 nm which is shifted to *λ*_max_ = 500 nm in LB films due to the formation of H-aggregates [251].

The redox potentials of these LB films are –0.73 V for the reduction and –0.384 V for the reoxidation (*vs.* SCE). LB films on ITO were used for photocurrent generation.

Aza-analogous 2,4,6-tristyrylpyridinium salts and 2,4,6-tris-(styrylstyryl)pyridinium salts proved to be efficient two-photon absorbing dyes with cross-sections up to 1600 GM at 800 nm [252]. Trisstyrylpyrylium analogues of **81** were prepared by condensation of trimethylpyrylium perchlorate with dimethylaminobenzaldehyde (97%) or dimethylaminocinnamic aldehyde (5%) [250]. Compared to the pyridinium salts, the absorption maxima are severely shifted to lower energies (*λ*_max_ = 685 nm and *λ*_max_ = 781 nm resp.).

A star-shaped three-dimensional cage compound (C-7-A-2) with two pyridine cores and three hexadecaoctayne handles has been observed by negative mode laser desorption TOF mass spectrum of a pyridocyclophane precursor [253]. Additionally, with low intensity, a peak due to a C_58_N_2_ anion was detected representing the first observation of a diazafullerene formed in a size-selective manner.

### 5.2. Stars with a Pyrimidine Core

*Triarylpyrimidines and Tri(alkynylaryl)pyrimidines (C-8-A-3 and C-8-A-9):*Like pyridine, the pyrimidine core displays a higher electron affinity and pseudo-*C*_3_-symmetry of 2,4,6-trisubstituted derivatives, but the pyrimidine is less basic compared to pyridine [254].

Two general methods for the synthesis of 2,4,6-triarylpyrimidines **84** are described in the literature [255]. The first method consists in the construction of the pyrimidine ring by condensation reactions [256,257,258], the second involves the functionalization of the pyrimidine ring. Trifluoromethanesulfonic anhydride appeared to be suitable for a co-condensation of benzonitriles with acetophenones yielding 2,4,6-triphenylpyrimidines **84** substituted with alkoxy side chains [259].

**Scheme 16:**
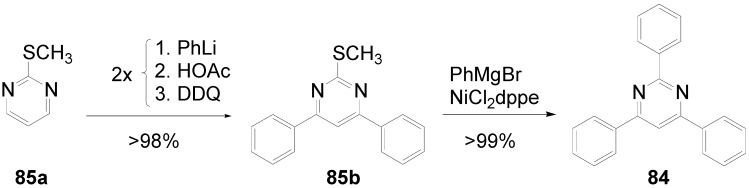
Successive threefold arylation of pyrimidine.

Aryl groups can be attached to the 2,4,6-positions of pyrimidine by successive arylation reactions (Scheme 16). Starting with 2-methylthiopyrimidine **85a** , an addition of an aryllithium reagent followed by oxidation with DDQ introduced the first aryl group in the 4-position, and the iterative procedure gives the 4,6-diarylpyrimidine **85b.** A NiCl_2_(dppe)-catalyzed substitution of the methylthio group with aryl-Grignard reagents led to the triphenylpyrimidines **84** with symmetrical or non-symmetrical substitution on the periphery [260].

**Scheme 17:**
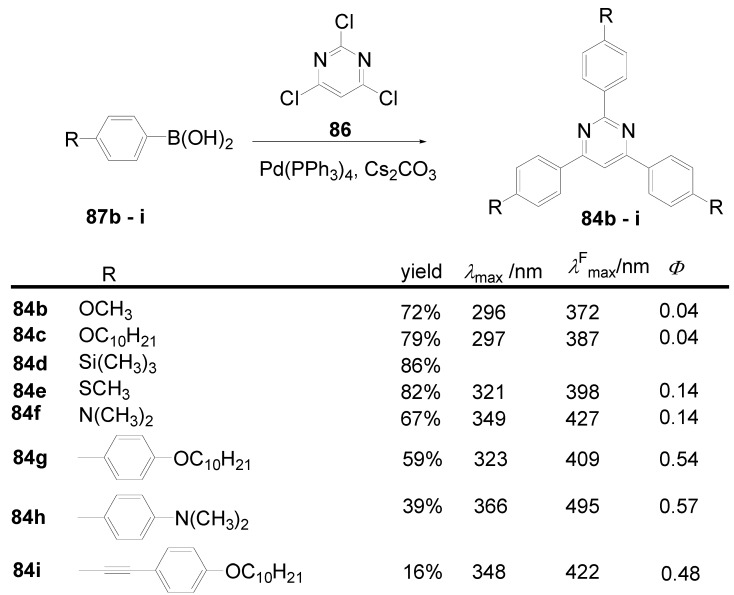
Synthesis of 2,4,6-triarylpyrimidines via threefold Suzuki cross-coupling.

The Suzuki cross-coupling reaction on chloropyrimidines [261] has become an important tool for the construction of 2,4,6-triarylpyrimidines **84** (Scheme 17). The π-electron deficient character of the pyrimidine ring is advantageous for the Suzuki reactions since it makes easier the oxidative addition of palladium to a carbon-chlorine bond without the use of specialized ligands [255].

Plé *et al*. synthesized star-shaped triarylpyrimidines **84b-i** via Suzuki coupling of 2,4,6-trichloropyrimidine **86** and an excess of boronic acids **87b-i**. With yields in the range of 72-86%, this method is suitable for boronic acids with moderate donor groups, strong donors like dimethylamino strongly reduce the electron deficient character of the pyrimidine and only a mono-coupling could be achieved, but with a modified procedure 2,4,6-tris(*p*-dimethylamino)pyrimidine **84f** could be obtained in 54% yield*.* This strategy is also useful for the construction of pyrimidine stars with biphenyl- (**84g, h**), tolanyl- (**84i**) and heterocyclic π-conjugated arms, but the yields decrease with increasing conjugation lengths.

The X-ray structure of **84b** shows that the molecule is slightly twisted in the solid state, the dihedral angle between the central pyrimidine and the 2-phenyl ring is only 3.6°, but the dihedral angles of the pyrimidine with the 4- and 6-phenyl ring are 13° and 14°. Nevertheless, these angles are sufficiently small to allow conjugation along the arms.

2,4,6-Triphenylpyrimidine **84a** is almost non-fluorescent [260]. Stars with moderate donor groups on the phenyl rings **84b, c, e** show absorption (*λ* = 296-321 nm) and emission (*λ*^F^ = 372-398 nm) in the UV, but with low quantum yields (**84b**: *Φ* = 0.04, **84**e: *Φ* = 0.14) [255]. The stronger dimethylamino donor group on the *p*-position of each phenyl ring **84f** provokes strong bathochromic shifts absorption (*λ* = 349 nm) and of the emission (*λ* = 427 nm) with a quantum yield of *Φ* = 0.14.

An unsymmetrical donor-substitution appears to be superior in terms of fluorescence efficiency. Only one dimethylamino group on the *p*-position of the 4-phenyl ring results in a highly emissive triphenylpyrimidine (in CHCl_3_: *λ*^F^_max_ = 444 nm, *Φ* ca. 0.6) that is strongly solvatofluorochromic with bathochromic shifts up to 184 nm [260]. The isomer with a 2-(*p*-dimethylaminophenyl) group is only weakly fluorescent. Compared to the tris-(*p*-methoxyphenyl)pyrimidine **84b** the unsymmetrical compound with a 4-dimethylaminophenyl substituent in the 4-position of the pyrimidine and two alkoxyphenyl rings shows a marked hypsochromic shift of the absorption (*λ*_max_ = 269 nm) and the emission (*λ*^F^_max_ = 306 nm) along with a strong increase of the quantum yield (*Φ* = 0.53).

In order to increase the electronic delocalization along each arm, stars with biphenyl and tolanyl groups were prepared by Pd-catalyzed coupling. Tris-4´-decyloxybiphenylyl- (**84g** 53%) and tris(4´-dimethylaminobiphenylyl)- (**84h** 39%) as well as tris-(4´-decyloxytolanyl)-pyrimidine (**84i** 16%) have been prepared [255]. Elongation of the π-system of the arms results in bathochromic shifts of the electronic spectra of Δ*λ* ≈ 24 nm for the **84g** and Δ*λ* ≈ 17 nm for the absorption of **84h**, but the emission maximum of the latter is shifted about Δ*λ* ≈ 67 nm to the red. The fluorescence quantum yields increased strongly to values of *Φ* = 0.54 and 0.57. A further increase of the conjugation length by introduction of a triple bond between the benzene rings (**84i**) results in further red shifts of the absorption (Δ*λ* = 25 nm) and the emission (Δ*λ*^F^ = 13 nm) and a small decrease of the fluorescence quantum yield (*Φ* = 0.48).

Absorption and emission properties of pyrimidine based stars with peripheral amino groups are sensitive towards changes of the environment. Compound **84h** (biphenyl π-bridge) with a donor-acceptor structure shows a strongly solvatochromic emission. Increasing the solvent polarity from heptane to DMF results in a bathochromic shift of 146 nm together with a reduction of the fluorescence quantum yield from 0.77 to 0.04. From solutions in methanol, no fluorescence could be detected. Contrary to the highly solvatochromic emission, the absorption maximum shifts only about 19 nm to the red. Addition of TFA to solutions of **84f** and **84h** results in a strong red shift of the emission of **84f** (from λ = 427 nm to λ = 539 nm) and a small shift of the emission of **84h** (Δ*λ*^F^ = 17 nm) but the absorption of **84h** is shifted to the blue (from *λ* = 366 nm to *λ* = 300 nm) These shifts are attributed to protonation of the peripheral amino groups of **84f** and **84h** [255].

In addition to their interesting optical properties, triarylpyrimidines are also electrochemically active compounds. Reversible reduction waves were obtained at –2.70-**–**2.85 V (*vs.* Ag/Ag^+^), the stars with amino groups showed an irreversible oxidation wave at ca. 0.4 V.

Alkoxy-substituted triphenylpyrimidine derivatives can exhibit hexagonal columnar mesophases. Comparing discs with identical numbers of side chains, those with an unsymmetrical substitution exhibit better mesomorphic properties. The improved LC behavior of the pyrimidine derivatives over the analogous discs with a benzene core is attributed to the greater polarization of N atoms in the ring [259].

**Scheme 18:**
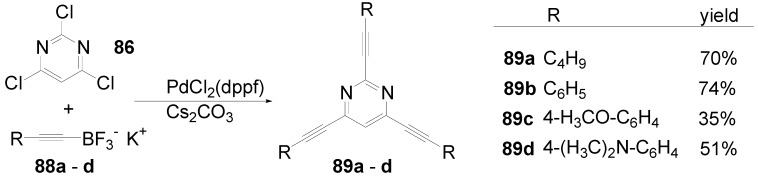
Synthesis of 2,4,6-tri(arylethynyl)pyrimidines via Suzuki coupling.

*Trialkynylpyrimidines (C-8-A-2 and C-8-A-8):* Threefold alkynylation of pyrimidine was not possible via Sonogashira or Negishi coupling, even when triiodopyrimidine was used as substrate. Molander [262] developed a Suzuki coupling of alkynyltrifluoroborates **88** with 2,4,6-trichloropyrimidine **86** and obtained 2,4,6-trihexynylpyrimidine **89a** in 70% yield (Scheme 18). Plé successfully applied this method for the synthesis of 2,4,6-tris-(phenylethynyl)pyrimidines, the unsubstituted star **89b** (74% yield), its trimethoxy **89c** (35%), and tris-(dimethylamino)-substituted derivatives **89d** (51%) [263].

DFT 6-31G* calculations of the geometry of tris-(phenylethynyl)pyrimidines **89** resulted in absolutely planar geometries with enforced conjugation whereas the triphenylpyrimidine derivatives **84** have a twisted geometry with dihedral angles in the range of 8-16°.

The absorption maxima of these compounds are in the UV (*λ*_max_ = 335-368 nm), only the dimethylamino derivative **89g** has an absorption maximum in the blue (*λ*_max_ = 433 nm). A comparison with banana-shaped 2,4- and 4,6-bis-(dimethylaminophenylethynyl)pyrimidines revealed bathochromic shifts of *λ*_max_. The maximum of the emission of these stars is strongly influenced by the donor substitution. A methoxy or dimethylamino group shifts *λ*^F^_max_ from 370 nm to 406 nm and 522 nm, respectively. But Stokes shift and fluorescence quantum yield increase in the order H< dimethylamino< methoxy. A 3,4,5-trimethoxy-substitution on each phenyl ring (**89e**) results in the largest Stokes shift (7681 cm^-1^) with λ^F^_max_ = 513 nm in CHCl_3_.

Compared with the 2,4,6-triphenylpyrimidines **84** [255,260] the elongation of the conjugated system provokes significant red shifts of the absorption and emission spectra (up to 95 nm for the dimethylamino derivatives) and increasing fluorescence quantum yields.

*Tristyrylpyrimidines (C-9-A-6):* 2,4,6-Tristryrylpyrimidine **90** has been prepared by base-catalyzed condensation of benzaldehyde with the *N*-oxide of 2,4,6-trimethylpyrimidine followed by reduction with PCl_3_ [264]. Better yields (44%) were obtained using ZnCl_2_ as catalyst for the condensation of benzaldehyde with 2,4,6-trimethylpyrimidine [265].

**Figure 28:**
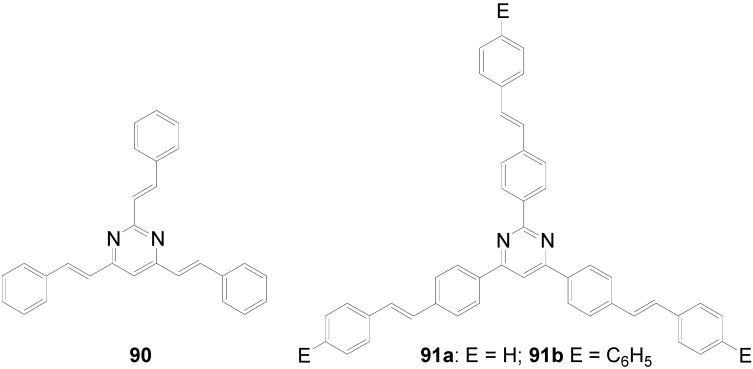
2,4,6-Trisstyryl- and 2,4,6-trisstilbenylpyrimidines.

*Trisstilbenylpyrimidine(C-8-A-7):* Threefold Siegrist reaction of 2,4,6-tris(4-methylphenyl)pyrimidine **84k** (R = CH_3_) with benzalanilines gave 2,4,6-trisstilbenylpyrimidine **91a** and tris-4´-phenylstilbenylpyridine **91b** in 33% yield [248].

The absorption of **91a** has a maximum at λ = 349 nm (DMF), extension of the π-system as in **91b** resulted in a red-shift, λ_max_ = 367 nm.

### 5.3. Pyrazine as Core (C-10-A-6)

Pyrazine as a core is an electron-deficient center for stars with a *C*_2h_-symmetry and the shape of St. Andrew´s cross. A multitude of tetraphenylpyrazines substituted with different functional groups has been prepared in the past, the first report about a tetraphenylpyrazine dates back to 1888 [266]. The typical procedure is the condensation of benzils or benzoins with ammonia from different sources. Some of these compounds have been suggested for application in high-temperature functional fluids [267], as photoconducting material for electrophotography [268], or as electron transporting layer in OLEDs [269].

Only a few pyrazines with four identical π-conjugated branches larger then a phenyl ring are known. The first syntheses of star-shaped-or cruciform-pyrazines with four styryl groups **92a** (R^1^ = N(CH_3_)_2_, R^2^ = H) was reported by Takahashi and Satake as part of their search for new photosensitizers [270]. Acid-catalyzed condensation of 4-dimethylaminobenzaldehyde (**95a** R^1^ = N(CH_3_)_2_) and tetramethylpyrazine **93** gave the pyrazine **92a** with four *p*-dimethylaminostyryl branches as orange-yellow prisms. Similarly, the higher homologue with 4-*p*-dimethylaminophenylbutadienyl branches **92b** and the *N*,*N*-dioxide **92c** of the tetrastyryl compound **92a** were prepared.

**Scheme 19:**
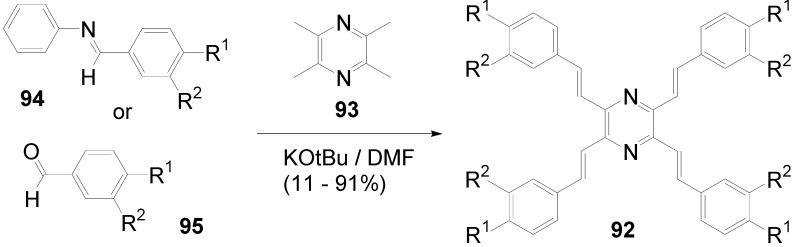
Condensation of tetramethylpyrazine **93** and benzalanilines **94** or benzaldehydes **95**.

4-Substituted tetrastyrylpyrazines **92** (R = H, Cl, CH_3_) have been used as emissive layer in OLEDs with the configuration ITO/TPD/**92**/Alq_3_/Mg. High luminescence (1516-4069 cd/m^2^) was achieved at low dc voltages [271].

Several 2,3,5,6-tetrastyrylpyrazines with alkoxy side chains in the 4-, the 3,4-, or the 3,4,5-positions of the peripheral benzene rings **92d**, **92e**, **92f** have been prepared via Siegrist reaction of tetramethylpyrazine **93** with benzalanilines **94** [272]. An eight-fold hexyloxy substitution of the tetrastryrylpyrazine **92d** is sufficient for the formation of a mesophase between 102 °C and 210 °C.

**Table 7:** Transition temperatures and enthalpies of liquid crystalline tetrastyrylpyrazine cruciforms (DSC, first heating scan).

Compound	Transition T ΔH (cal/g)	Transition T ΔH (cal/g)
**92d**: 3,4-dihexyloxy	Cryst → Col 102 °C (14.0)	Col → i 210 °C (0.4)
**92e**: 3,4-didecyloxy	Cryst → Col 101 °C (19.3)	Col → i 173 °C (1.5)
**92f**: 3,4-didodecyloxy	Cryst → Col 95 °C (18.8)	Col → i 172 °C (0.7)

Tetrastyrylpyrazines with an alkoxy side chain in the p-position of all peripheral benzene rings show a broad absorption spectrum with λmax = 390 nm (CH2Cl2) and a second maximum at λmax = 461 nm. A peripheral 3,4-dialkoxy substitution (e.g., 92d) shifts both maxima about 4 to 7 nm to the red. Further alkoxy groups in all 5-positions provoke an additional small hypsochromic shift (λmax = 389 nm, λ´max = 455 nm) [272]. The fluorescence of tetrastyrylpyrazines with a 4-alkoxy substitution is slightly solvatochromic, increasing solvent polarity shifts the fluorescence maximum from λFmax = 509 nm (cyclohexane) to λFmax = 527 nm (ethanol) [273].

Within this tetrastyrylpyrazine series, compounds substituted with dialkylamino groups in the 4-positions of the benzene ring appear to be most attractive. Several syntheses of these compounds have been reported [185,270,273,274]. Acid- or base-catalyzed condensation of tetramethylpyrazine **93** with dialkylamino benzaldehydes or their aniline derived Schiff bases gives these cruciforms **92a**, **92g**, in moderate to very good yield.

**Figure 29:**
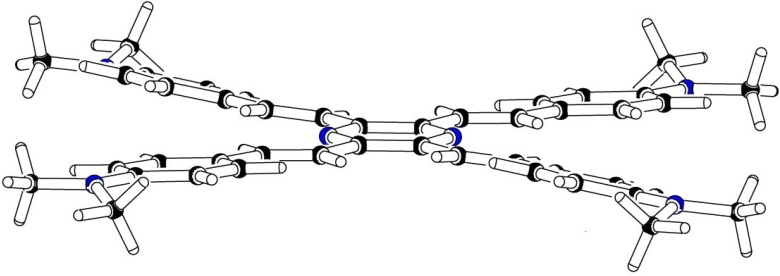
X-ray structure of 2,3,5,6-tetrakis(*p*-dimethylaminostyryl)pyrazine **92a**.

X-ray analysis of the dimethylamino derivative **92a** shows a nearly planar molecule [273], but the two linear distyrylpyrazine subunits are not equivalent (Figure 29). Whereas one shows small dihedral angles between the aromatic rings and the vinylene groups of –0.3° and –177.7°, the other is more distorted. Torsion angles of 8.6° and-174.4° have been measured.

The absorption spectrum of 2,3,5,6-tetrakis(4-diethylaminostyryl)pyrazine **92g** shows a structured main absorption band with *λ*_max_ = 452 nm and a structureless separate peak at *λ*_max_ = 498 nm. The fluorescence in toluene peaks at *λ*_max_^F^ = 563 nm with a fluorescence quantum yield of 0.67 [185].

Compared to the linear 2,5-bis-(4-diethylaminostyryl)pyrazine **96**, (*λ*_max_ = 462 nm, *λ*^F^_max_ = 518 nm, *Φ* = 0.77) the emission is shifted to the red side and occurs only from the lower lying of the two states observed in the absorption spectrum.

A pronounced sensitivity of the fluorescence of tetrakis(dialkylaminostyryl)pyrazines **92a,g** was reported [273,274], e. g. the emission maximum of **92a** appears in cyclohexane at *λ*^F^_max_ = 535 nm and in ethanol at *λ*^F^_­max_ = 588 nm.

The two-photon absorption properties of these stars with strong ICT transitions have been studied. Harper reported a two-photon absorption cross-section of δ = 1600 GM at *λ* = 800 nm [274]. According to the results from two-photon spectroscopy, Marder *et al*. [185,275] could show that the TPA properties of the linear 2,5-bis(diethylaminostyryl)-pyrazine **96** and the cruciform **92g** are quite similar, a TPA cross-section δ_max_ of 1250 GM has been measured for both compounds, the TPA absorption maximum of the linear compound λ^TPA^_max_ = 770 nm is shifted about 20 nm to the red for the cruciform (λ^TPA^_max_ = 790 nm). The similar TPA cross-sections of linear and cruciform dye result from a significant coupling of the branches through the common central ring.

### 5.4. 1,3,5-Triazine as Core

*Triaryltriazines (C-9-A-3):* Within the series of star-shaped oligomers with a heterocyclic core, the 1,3,5-triazine ring C-9 plays the most prominent role. In these stars, the π-conjugated branches are connected with *C*_3h_-symmetry to a highly electron-deficient core. Synthetic approaches include the formation of triazines via cyclotrimerization of nitriles, nucleophilic and palladium-catalyzed substitutions on trifluoro- or trichloro-1,3,5-triazines as well as transformations of functional groups in the periphery.

**Scheme 20:**
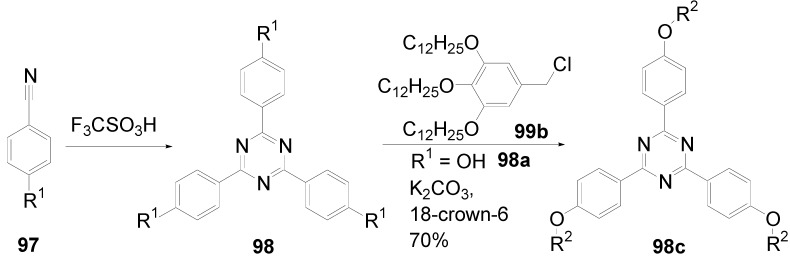
Cyclotrimerization of benzonitriles.

**Scheme 21:**
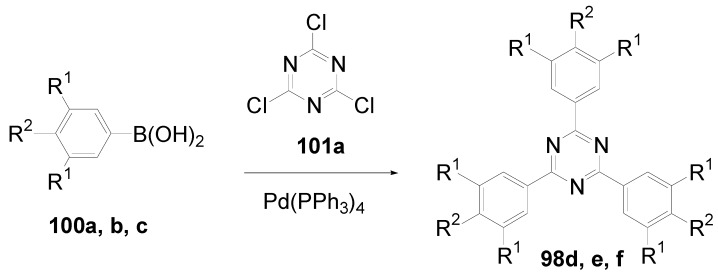
Synthesis of 2,4,6-triaryl-1,3,5-triazines **98d, e, f** via Suzuki coupling.

The trimerization of nitriles **97** (Scheme 20) catalyzed by acid [277] or base [278,279] is the classical synthetic strategy for the preparation of symmetrically substituted 2,4,6-triaryl-1,3,5-triazines **98**. Recently, coupling reactions of organometallic reagents with cyanuric chloride **101a** (Scheme 21), direct [280] and palladium-catalyzed [255,281,282], have become the preferred way.

Tris-(4-hydroxyphenyl)-1,3,5-triazine **98a** was prepared from *p*-hydroxybenzonitrile **97a** using trifluoromethane sulfonic acid in 93% yield. Alkylation of the hydroxy groups with 3,5-di- and 3,4,5-trialkoxybenzylchlorides **99a, b** gave star-shaped compounds **98b**, **c** [277]. Contrary to the pure tris-tridodecyloxybenzyl substituted compound **98c**, its orange-red CT complex with trinitrofluorenone forms a columnar mesophase between 50.6 °C and 101.5 °C (first heating scan) which remains stable at room temperature.

3,4,5-Trialkoxybromobenzenes were converted to the boronic acids **100a, b** and threefold Suzuki coupling reactions with cyanuric chloride **101a** in toluene gave the triphenyltriazine **98d** with nine decyloxy side chains in 52% yield and the isomer **98e** with (3*S*)-3,7-dimethyloctyl side chains in 33% yield [281]. Both compounds show enantiotropic LC behavior with mesophases from 36 to 145 °C (**98d**) and –15 °C to 56 °C (**98e**) on heating. According to the textures observed in POM and to X-ray diffraction, the former star exists in a hexagonal columnar mesophase whereas the LC phase of the latter has a rectangular columnar structure.

UV-vis absorption shows a broad absorption band at 318 nm. A strong circular dichroism of **98e** suggests that the discotic molecules are stacked along the columns, while tilted with respect to the column axis, to form a left-handed helix within the column. The non-centrosymmetric arrangement of these discs should result in nonzero bulk second order NLO properties.

The threefold Suzuki coupling of 4-dimethylaminobenzeneboronic acid **100c** with cyanuric acid chloride using Cs_2_CO_3_ as a base provided 2,4,6-tris-(4-dimethylaminophenyl)-1,3,5-triazine **98f** in 42% yield [255]. In CHCl_3_ the absorption maximum appears at 368 nm and the fluorescence maximum at 419 nm with a quantum yield of *Φ* = 0.15.

**Scheme 22:**
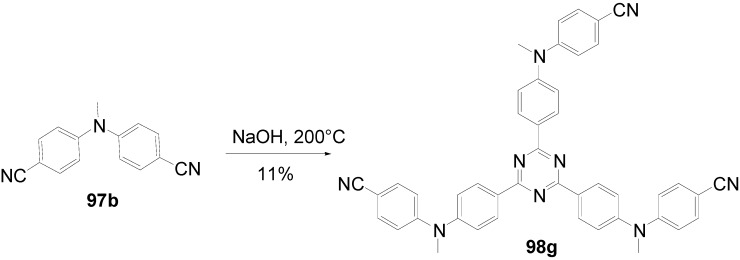
Base-catalyzed trimerization of dinitrile **97b.**

Fujita [278] used drastic conditions (NaOH, 200 °C, 36h, Scheme 22) for the oligomerization of *N*,*N*-di-(*p*-cyanophenyl)-*N*-methylamine **97b**, giving a mixture of branched oligomers including 11% of the star-shaped compound **98g**. The absorption spectrum is dominated by a long-wavelength ICT band with *λ*_max_ = 387 nm-about 100 nm at longer wavelengths compared with the analogous stars without peripheral amino groups. Though intramolecular charge transfer occurs, the compounds show a negligible solvatochromism. A pronounced acidochromism results from the successive addition of trifluoroacetic acid: in the first step, a new maximum at *λ*_max_ = 480 nm appears-attributed to protonation of the triazine core and therefore enhanced acceptor strength. At higher TFA concentrations a third compound with *λ*_max_ = 577 nm appears, changing the solution from colorless (neutral) and orange (10% TFA) to wine-red (60% TFA). The authors suggest a protonation on the nitriles. An intense blue fluorescence (*λ*^F^_max_ = 434 nm, *Φ* = 0.66) is emitted from neutral solutions, bathochromic shifts occur in protic solvents and acids efficiently quench the fluorescence.

The trifluoromethane sulfonic acid catalyzed cyclotrimerization of *p*-bromobenzonitrile **97c** to 2,4,6-tris-4-bromophenyl-1,3,5-triazine **98h** has become a key step in the preparation of stars with a 1,3,5-triazine core [275,282,283,284,285].

**Scheme 23:**
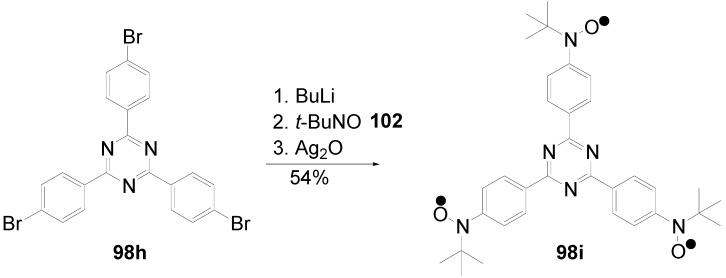
Synthesis of **98i**, a triazine-based triradical.

Lithiation of the tribromo compound **98h** and addition of 2-methyl-2-nitrosopropane **102** followed by Ag_2_O oxidation gave the triradical **98i** in 77% yield (Scheme 23). The triangular triradical shows dihedral angles between the phenyl rings and the central triazine units of 9.96, 10.29 and 5.37°, respectively. In solution, **98i** gives an EPR signal with a seven-line hyperfine structure at *g* = 2.0061 suggesting that the exchange interaction is larger than the hyperfine coupling [275]. Compared to the analogous triradicals with a benzene core, **98i** exhibited stronger intramolecular ferromagnetic interaction than the benzene analogue.

2,4,6-(Tris-diethylaminophenyl)-1,3,5-triazine **98k** was prepared by the reaction of 4-lithio-*N*,*N*-diethylaniline and **101a** in 20% yield [280]. The strong donor effect of the diethylamino group is visible in the UV-vis spectrum as *λ*_max_ = 375 nm (acetone) and gives a large second-order polarizability (hyper-Rayleigh-scattering) with *β* = 162 × 10^-50^ Cm^3^V^-2^, six times larger than that of *p*-nitroaniline (*β* = 27.4 × 10^-50^ Cm^3^V^-2^).

The first hyperpolarizabilities of symmetrically substituted 1,3,5-triazines are higher than those of corresponding octupolar benzenes [286]. The triazine ring seems to be a better acceptor than the benzene ring but if it acts as a donor as in symmetrically substituted triphenyltriazines the nonlinearity improves further. *Ab initio* calculations (HF/6-31G) of donor-substituted 2,4,6-triphenyl-,2,4,6-tristyryl-, and 2,4,6-tris(phenylbutadienyl)-1,3,5-triazines resulted in planar structures and significant *β* values [287].

**98h** is a suitable substrate for three-fold copper-catalyzed *N*-arylations, e.g., with 7-azaindole [288]. Ullmann reaction with di-2-pyridylamine gave a star **98l** with triarylamine branches in 60% yield. This compounds has a melting point at 267 °C, DSC shows a glass transition at 121 °C, and a second heating scan revealed a broad crystallization peak around 180 °C [289].

The UV absorption appears with a double maximum at *λ* = 322 and *λ* = 386 nm and the blue fluorescence, both, from solution or the solid state, has a maximum at *λ*^F^_max_ = 440 nm (*Φ* = 0.78).

In diodes with the configuration ITO/CuPc/**98l**/PBD/LiF/Al blue light was emitted with a turn-on voltage of 15 V. This turn-on voltage is higher than that of the analogous compound with a benzene core, probably due to poor electron/hole mobility.

2,4,6-Tris-4-pyridyl-1,3,5-triazine **103** has been prepared by base-catalyzed (KOH/18-crown-6, decaline, 200 °C) cyclotrimerization of 4-cyanopyridine [279]. It has been used as a tridentate ligand in supramolecular nanocages [290] and 2,4,6-tripyridyl-1,3,5-triazines have been found to be efficient materials for electron injection layers in transparent OLEDs [291].

*Triazines with Biaryl Arms (C-9-A-3):* **98h** is a key compound for the synthesis of triazines with biaryl branches [292,293,294]. 2,4,6-tris(4-(4-pyridyl)phenyl)-1,3,5-triazine **98n** was obtained in 66% yield via Stille coupling with 4-pyridylstannanes in the presence of LiCl [292].

**Scheme 24:**
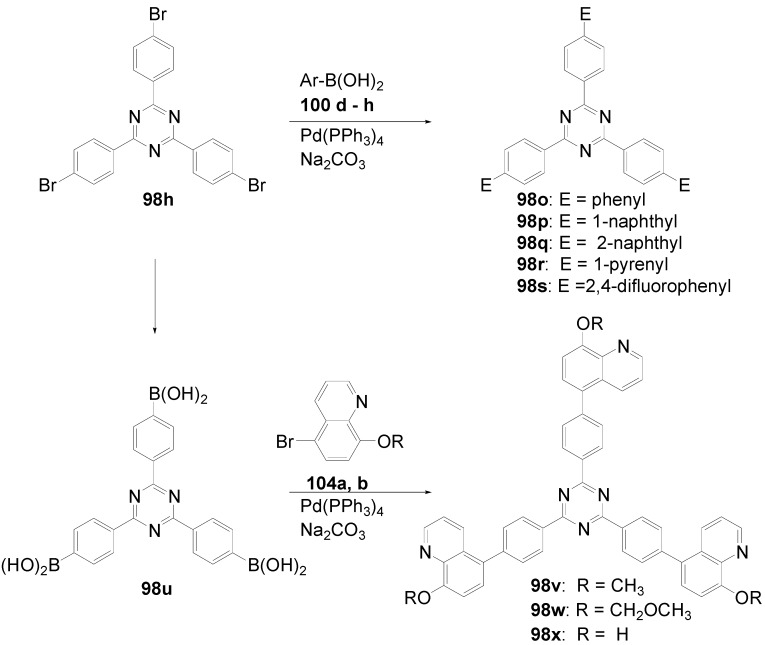
Synthesis of tris(biaryl)triazine stars via Suzuki coupling.

Threefold Suzuki coupling reactions of **98h** (Scheme 24) with benzeneboronic acids **100d-h** substituted in the 4-position with phenyl, 2,4-difluorophenyl, 1- and 2-naphthyl or pyrene gave the star-shaped compounds **98o**-**s** in good yields [293].

The half-wave reduction potentials of these compounds are close to –2.1 V (*vs.* Fc/Fc^+^) suggesting that the electron affinity of the triazine core is only slightly affected by the terminal groups. The 1-naphthyl derivative **98p** possesses a high glass transition temperature at 133 °C, time-of-flight measurements on vacuum-deposited films of this star showed a high electron drift mobility of 8 × 10^-4^cm^2^V^-1^s^-1^ at 25 °C, therefore, this compound is an attractive amorphous glassy electron transporting material.

Wang described a 2,4,6-tris-biphenyltriazine star **98t** with six *N*-indolyl groups on the 3,5-positions of the terminal rings [288]. The key step for the synthesis is a Suzuki coupling of the 3,5-bis(*N*-indolyl)benzeneboronic acid **100i** with **98h** (61%). DSC reveals that this star has a glass transition at 175 °C, the melting point is above 330 °C. In CH_2_Cl_2_, the long-wavelength absorption band appears at *λ* = 306 nm and the fluorescence with *λ*^F^_max_ = 481. The analogous compound with a benzene core has a similar absorption, but hypsochromically shifted emission (*λ*^F^_max_ = 427 nm) due to a reduced charge transfer.

The inverted functionalization, a central trisboronic acid **98u** and 5-bromoquinolines **104a, b** as coupling partners proved to be suitable for the synthesis of stars **98v, w** with a 8-alkoxyquinoline periphery (Scheme 24) [294]. The absorption maximum of **98v** with three 8-methoxyquinoline end groups appears at *λ* = 340 nm and the emission peaks at *λ*^F^ = 443 nm (CH_2_Cl_2_, *Φ* = 0.63), characterized by a strongly positive solvatochromism. The electrochemical oxidation of this compound occurs at 1.51 V (*vs.* AgCl/Ag) and the quasi-reversible reduction at –1.73 V. Star **98x** with unprotected 8-hydroxy groups forms complexes with triphenylboron. Complexation shifts absorption and emission spectra about Δ*λ* ≈ 80 nm to longer wavelengths.

Acid-catalyzed cyclotrimerization [289] of 4-bromo-4´-cyanobiphenyl **97d** gives 2,4,6-tris(4´-bromobiphenyl)-1,3,5-triazine **98y** (80%). Using a Cu-catalyzed Ullmann reaction with 2-(2´-pyridyl)benzimidazole **105**, triazine-based star **98z** with three chelating pyridylbenzimidazole end groups was obtained in 68% yield [295]. After complexation with ruthenium, **98z** was used in red light-emitting devices.

**Scheme 25:**
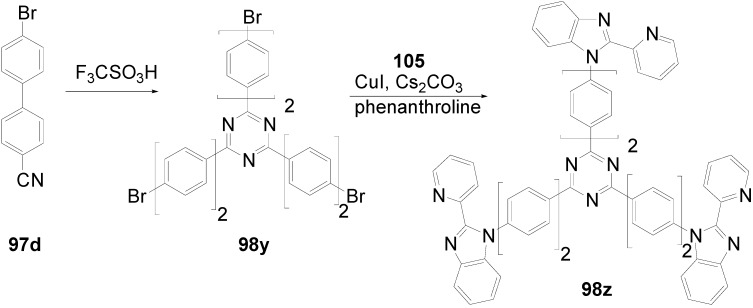
Triazin-star **98z** with chelating pyridyl-benzimidazole periphery.

Lithiated 2-bromo-7-diphenylamino-9,9-dialkylfluorenes **106a-c** reacted with cyanuric fluoride **107** to the tris-(diphenylaminofluorenyl)-1,3,5-triazines (Scheme 25) with six allyl (**98aa**), 3,7-dimethyloctyl (**98ab**) or decyl groups (**98ac**) in 51 to 60% yield [296]. The UV-vis absorption of these aminofluorene stars peaks around 413 nm and emission shows maxima between 500 and 518 nm with high quantum yields (*Φ* ≈ 0.47). The two-photon absorption cross-sections (excitation at *λ* = 800 nm) were found to be in the range of 278-395 GM, compounds with longer aliphatic chains gave higher cross-sections.

The photophysical properties of star **98ab** were thoroughly analyzed by Rogers [297]. A slight solvatochromism of the absorption spectrum (e.g., in hexane *λ*_max_ = 418 nm, in THF *λ*_max_ = 414 nm) and a strong solvatochromism of the fluorescence (hexane: *λ*^F^_max_ = 432 nm, *Φ* = 0.75; 2-propanol *λ*^F^_max_ = 516 nm, *Φ* = 0.25) was observed. In hexane, the fluorescence occurs predominantly from the S_1_ state, in more polar solvents, the emission occurs from both, the S_1_ and the ^1^ICT state. Ultrafast transient absorption spectra revealed a multiexponential decay. In the presence of methyl iodide (20% in methylcyclohexane) a phosphorescence with *λ*^Ph^_max_ = 546 nm was detected. **98ab** has a triplet excited-state absorption in the region of 800 nm that enhances the effective two-photon absorption cross-section.

**Scheme 26:**
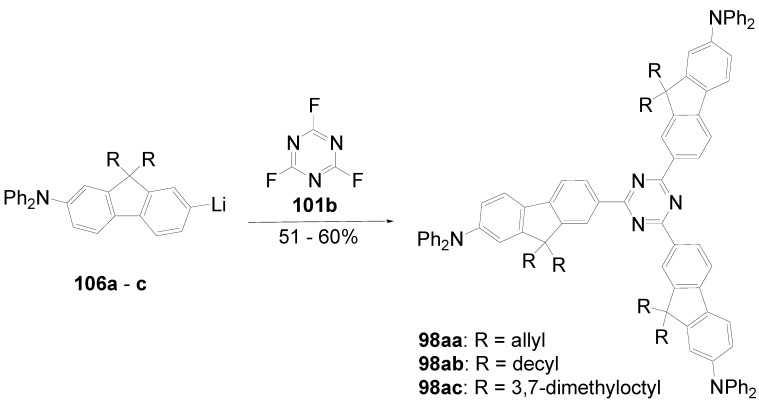
Triazine-stars with fluorene branches.

Tris-(spirobiphenyl)-1,3,5-triazine **98ad**, an electron transport host materials for green phosphorescent OLEDs, has been prepared by the acid-catalyzed cyclotrimerization of 2-cyano-9,9´-spirobifluorene 97e in 63% yield [298]. The time-of-flight electron mobility was higher than 10^-4^ cm^2^ V^-1^ s^-1^. A relatively low triplet energy of 2.54 eV and a HOMO energy level of 5.69 eV (PES) were found. An OLED with **98ad** as electron transporting material and (PPy)_2_Ir(acac)/1,3,5-tris-*N*-benzimidazolylbenzene gave only a low external efficiency of 5.1%.

Some triazine stars carrying heterocyclic biaryl branches exhibiting interesting optical and nonlinear optical properties have been prepared by triple nucleophilic aromatic substitution of α-lithiated bithiophenes and cyanuric chloride in up to 90% yield [299]. Also the Stille coupling [300] proved to be useful.

**Scheme 27:**
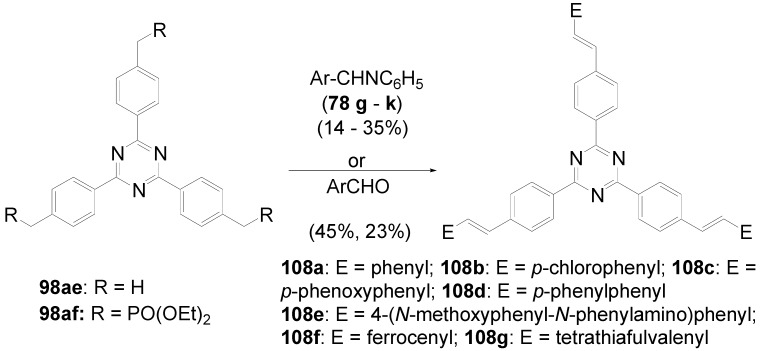
Three-fold Siegrist and Horner olefinations to trisstilbenyltriazines.

*Triazines with Three Stilbene Arms (C-9-A-7):* Threefold condensation of 2,4,6-tris(4-methylphenyl)-1,3,5-triazine **98ae** with benzalaniline **78g** has been developed by Siegrist [248] for the synthesis of trisstilbenyl-1,3,5-triazine **108a** and derivatives **108b**-**e** with substituents in the 4´-positions of all stilbene arms in 14-35% yield (Scheme 27) The absorption spectrum of **108a** has a maximum at *λ* = 360 nm (DMF). Whereas a chlorosubstitution in the 4´-positions of all stilbene units (**108b**) provokes only a small hyperchromic effect, donor-substitution (**108c**: 4´-phenoxy, λ_max_ = 380 nm) or extension of the conjugated system (**108d**: 4´-phenyl, *λ*_max_ = 375 nm) led to significant red shifts of the absorption spectrum.

Similarly, a three-fold Siegrist reaction was used by Tian [301] for the synthesis of a trisstilbenyltriazine star **108e** with three peripheral *N*-4-methoxyphenyl-*N*-phenylamino groups (50% yield). An absorption peak at *λ* = 312 nm corresponds to a localized excitation, the band centered at *λ* = 430 nm to a π-π* transition of the whole molecule. Compared to stars with only one or two stilbene arms absorption and emission suffer minor shifts to lower energies. The absorption of star **108e** is slightly solvatochromic, a red-shift of 7 nm in DMSO was recorded. Upon irradiation at *λ* = 415 nm, an intense fluorescence was emitted, with *λ*^F^_max_ at 550 nm in CHCl_3_ (*Φ* = 0.47) and-hypsochromically shifted-with *λ*^F^_max_ = 530 nm in DMSO [302]. The state responsible for the photoluminescence is the ICT state. The transient absorption shows a biexponential decay with much higher decay rates in DMSO. The long decay time is attributed to the lifetime of the ICT state, and the shortening of the decay time was explained by a TICT model. From Z-scan measurements the TPA cross-section *σ*_2_ at *λ* = 800 nm was calculated to be *σ*_2_ = 410 GM and therefore five times higher than the triazine with only one stilbene unit. The two-photon excited emission is located at *λ*_’max_ = 577 nm.

An independent approach for the synthesis of triazine stars with three arylethenylphenyl branches has been reported by Martín [303]. The triflic anhydride catalyzed cyclotrimerization of *p*-bromomethylbenzonitrile **97f** followed by Michaelis-Arbuzov reaction gave a triphenyltriazine **98af** with three methylenephosphonate groups as useful building block. Threefold Horner reaction with formylferrocene or formyltetrathiafulvalene resulted in redoxactive stars **108f, g** in 45% and 23% yield.

Ferrocene-star **108f** absorbs with *λ*_max_ = 484 nm (CHCl_3_) and the TTF-star **108g** with *λ*_max_ = 471 nm (CHCl_3_). The intramolecular charge transfer in **108f** was confirmed by solvatochromic studies, a positive solvatochromic shift of 25 nm was recorded from hexane to ethanol. Both stars show an amphoteric redox behavior, with the oxidation features of the donor moieties and the reduction of the triazine acceptor. A significant electronic communication between the triazine and the TTF was observed.

**Scheme 28:**
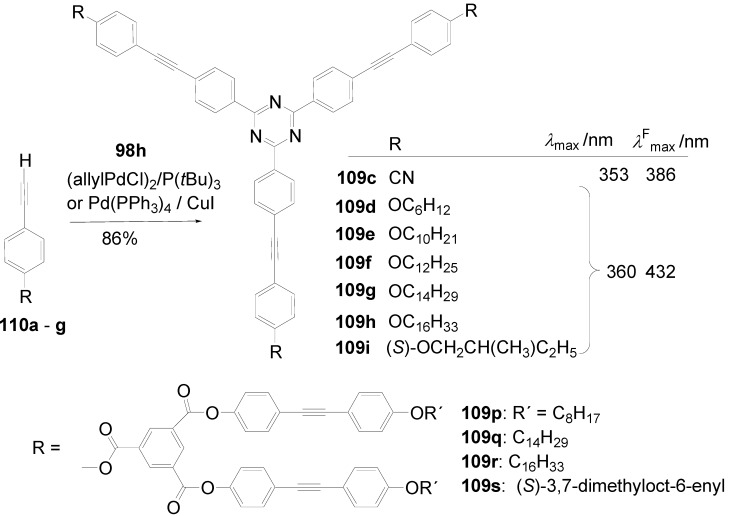
Synthesis of tris-tolanyl-1,3,5-triazines via Sonogashira cross-coupling reactions.

*Tris(ethynylaryl)triazines (C-9-A-9):* 1,3,5-Triazines with three tolane branches **109** were successfully prepared by threefold Sonogashira-Hagihara cross-coupling reactions on tris-(*p*-bromophenyl)-1,3,5-triazine **98h**. The stepwise approach (coupling with TMS-acetylene to **109a** (E = TMS), deprotection to tris(ethynylphenyl)-1,3,5-triazine **109b** (E = H) and second coupling of the deprotected star with iodoarenes [282] as well as the direct coupling with phenylacetylenes **110a-g** [283,284,285] have been used (Scheme 28).

An X-ray structure analysis of 2,4,6-tris-(4´-cyanotolanyl)-1,3,5-triazine **109c** reveals a non-planar structure with a sphere-surface like shape. The average deviation of the atoms from the mean plane of the molecule is 0.789 Ǻ [284]. The UV-Vis spectrum of 2,4,6-tris-(4´-cyanotolanyl)-1,3,5-triazine **109c** peaks at 353 nm and a sharp emission peak is observed at *λ*^F^**** = 386 nm.

Tris-tolanyl-1,3,5-triazines with three peripheral alkoxy side chains (**109d-i**) have been prepared by Lee and Yamamoto [285]. Chains lengths of 6, 10, 12, 14, and 16 carbons as well as (*S*)-2-methylbutyl were investigated. Whereas the compounds with short linear or chiral side chains **109d**, **i** are not liquid crystalline materials, the stars with the longer chains exhibited columnar hexagonal disordered mesophases (Col_hd_). Stars with decyl (**109e**) or dodecyl chains (**109f**) showed a transition into a smectic phase at 57 °C and 116 °C, followed by a transition into a Col_hd_ phase at 67 °C and 124 °C and a transition into the isotropic melt at 141 °C and 129 °C. Upon cooling, all transitions were shifted about 5-10 °C to lower temperatures. The homologs with longer chains **109g, i** showed transitions to their Col_hd_ phases at 66° (**109g**) and 105 °C (**109h**) and into the isotropic phases at 105 °C and 116 °C.

The UV-vis absorption of tris-(4´-alkoxytolanyl)-triazine stars **109d-i** peaks at *λ* = 360 nm and the fluorescence maximum appears at *λ*^F^ = 432 nm (CHCl_3_; *Φ* = 0.85). In cast films, the absorption and emission spectra are shifted to longer wavelengths (typically *λ*_max_ = 385 nm, *λ*^F^_max_ = 491 nm). Due to the high quantum yields and the stability, these compounds have a potential applicability as laser dyes.

Analogous tris-tolanyl-1,3,5-triazines **109k-o** with six peripheral alkoxy side chains of 9-14 carbon length on the 3,4-positions of the outer benzene rings were investigated as LC materials. Only compounds with decyl **109l** or undecyl chains **109m** showed mesogenic properties. Transition into the columnar hexagonal disordered mesophase occurred at 85.6 °C (**109l**) and 92.0 °C (**109m**) and to the isotropic liquid at 99.4 °C and 102.7 °C, respectively. Compared to the related compounds with only one alkoxy group in the 4-position of the peripheral rings **109e, f**, the electronic transitions are shifted to lower energies. Dissolved in CHCl_3_, their UV-vis absorption peaks at *λ*_max_ = 365 nm and their fluorescence at *λ*^F^_max_ = 476 nm. Cast films showed similar absorption maxima but shifts of the emission about 30 nm to the red. These shifts are associated with the formation of excimer-like adducts.

A threefold Pd-catalyzed coupling of tris-(ethynylphenyl)-1,3,5-triazine **109b** with an *p*-iodophenyl benzoate carrying tolanyloxy esters in the 3- and 5-positions gave star-burst compounds with a tris-tolanyl-1,3,5-triazine core and branched tolanylester groups **109p-s** [282]. Liquid-crystalline phases with columnar hexagonal disordered structure (POM) were obtained between 132 °C (cryst. to mesophase) and 229 °C (mesophase to isotropic) depending on the nature of the peripheral alkoxy side chains.

*Tristyryltriazines (C-9-A-6):* The first report of a 2,4,6-tristyryl-1,3,5-triazine **111a** (R^1-3^ = H) dates back to 1953 [304]. Changing the original conditions for the threefold condensation of 2,4,6-trimethyl-*s*-triazine **112** with benzaldehyde from KOH/methanol to concentrated sulfuric acid as dehydrating agent by Elias [305] increased the yield from 31% to 99% of analytically pure material. Nevertheless, the base-catalyzed aldol condensation is the generally applied strategy for the synthesis of tristyryl-substituted

1,3,5-triazines **111**. Higher homologues are accessible either via Horner olefination of a tristyryltriazine carrying methylenephosphonates on the benzene rings (**111b** R^1^ = R^3^ = H, R^2^ = CH_2_PO(OEt)_2_) or, generally more efficient, via direct aldol condensation of **112** and stilbenoid aldehydes **107** [306].

The acceptor effect of the triazine causes an intramolecular charge transfer which results in a bathochromic shift of the lowest-energy transition. The parent tristyryltriazine **111a** has an absorption at *λ* = 327 nm, the methoxy derivative **111c** at *λ* = 356 nm and the dimethylamino derivative **111d** at *λ* = 425 nm (CH_2_Cl_2_).

**Scheme 29:**
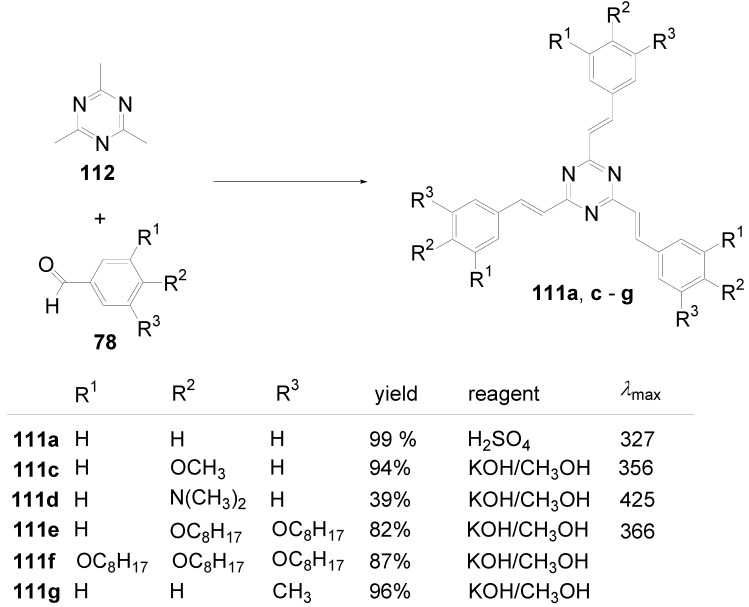
Threefold aldol-condensations of 2,4,6-trimethyl-1,3,5-triazine.

2,4,6-Tristyryl-1,3,5-triazines **111** with 3, 6, or 9 peripheral alkoxy side chains (linear and branched) are disc-like molecules which can exhibit a regular aggregation by π-stacking and by the interaction of the side chains. Whereas three side chains, irrespective of their length, are not sufficient for the formation of thermotropic liquid crystals, six octyloxy side chains (**111e**) result in the formation of a narrow mesophase (75°-82 °C) [307]. The compounds with nine linear hexyloxy side chains (**111h**) shows transition temperatures T_m_ = 68.4 °C and T_c_ = 112 °C. Elongation of the side chains gives a minimum of the transition temperatures for 9 decyloxy groups (**111i**) with T_m_ = -23 °C and T_c_ = 90 °C. Further elongation results in an increase of T_m_ = 50.3 °C for hexadecyloxy (**111k**), but T_c_ is lowered to 77.9 °C. All mesophases were recognized as hexagonal columnar phases Col_hd_. The star with nine hexyloxy side chains **111h** exists in the solid state in a helical columnar arrangement, which is transformed to disordered hexagonal columns in the mesophase.

Irradiation (*λ* = 366 nm) in the mesophase of tristyryltriazines with nine alkoxy groups **111f, h-k** provokes partial cyclodimerization reactions which cause lower clearing points. Since the diffusion processes of the dimers are very ineffective, the borderline between the irradiated and the original LC phase is preserved, even in the molten state. In the solid state, the compound proved to be photostable, dimerization occurs only if the mobility of the molecules is enhanced, e.g., in the mesophase. Thus, the compounds seem to be suitable for an optical data storage in LC materials.

**Scheme 30:**
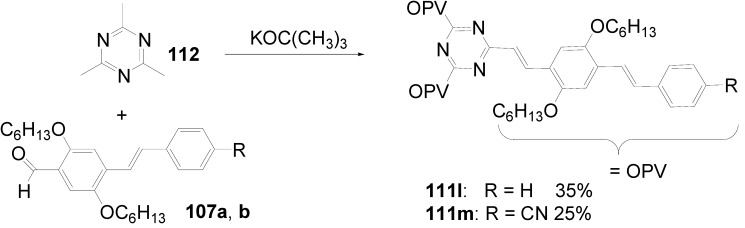
Synthesis of triazine stars with alkoxy groups in the inner sphere.

Meier *et al*. [112] prepared tris(styrylstyryl)-1,3,5-triazines **111l**, **m** with a unique substitution pattern (Scheme 30): the alkoxy side chains are attached to the 2,5-positions of the inner rings of the conjugated arms and not in the periphery of the molecules. These compounds exhibit nematic mesophases **111l**: T_g_ = 95 °C N_D_ and T_c_ = 107 °C and **111m** between 210 °C and 236 °C. The peripheral cyano groups on **111m** lead to an acceptor-donor-acceptor character resulting in a strong increase of the phase transition temperatures. The undercooling and the ΔH high values of 35 kJmol^-1^ (**111l**) and 49.8 kJmol^-1^ for the isotropization indicate N_col_ phases.

The LC phases are light-sensitive, irradiation with UV (*λ* = 366 nm) or daylight leads to a breakdown of the mesophases due to intermolecular C-C-bond formation generating cross-linked oligomers and polymers. This can be useful for imaging techniques with liquid crystals. In the crystalline state irradiation provokes a chemoselective [2π + 2π] cycloaddition between the inner, more polar double bonds with a head-to-tail regioselectivity and stereoselective *syn* arrangement and a preservation of the *trans* configuration from the olefinic double bonds in the cyclobutanes.

**Scheme 31:**
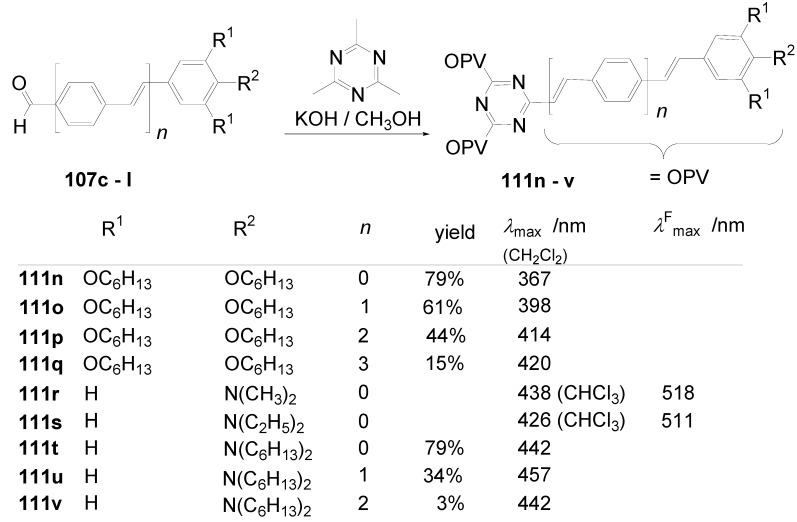
Homologous series of donor-substituted tris(oligostyryl)triazines.

The extension of the π-conjugated system in a homologous series of tris(oligostyryl)-1,3,5-triazines (Scheme 31) **111n-q** with a peripheral 3,4,5-trialkoxy substitution leads to a systematic bathochromic and hyperchromic effect (*n* = 1: *λ* = 367; *n* = 2: *λ* = 398; *n* = 3: *λ* = 414; *n* = 4: *λ* = 420 nm; *n* = styryl units per arm) [306]. The convergence limit, calculated on the basis of exponential functions [28] is predicted as 427 ± 3 nm. The analogous stars **111t-v** with a stronger *p*-dihexylamino donor and one to three stryryl units per arm (*n* = 1-3) behave different [308].

The unsubstituted 2,4,6-tristyryl-1,3,5-triazine **111a** has an absorption maximum in the UV (*λ* = 327 nm), the absorption spectra of compounds with amino end groups **111r-v** are strongly shifted to the red due to an intramolecular charge transfer from the amino groups to the acceptor core. This ICT depends strongly on the distance of lateral donors and the central triazine ring (*n* = 1: *λ*_max_ = 442 nm: *n* = 2: *λ*_max_ = 457 nm; *n* = 3: *λ*_max_ = 442 nm).

The transition energy Δ*E*(S_0_→S_1_) of push-pull oligomers is lowered by the extension of the conjugation and by the ICT. Both effects are superimposed and can lead to four different functions *λ*_max_(*n*) where *n* represents the number of repeat units in a conjugated oligomer series [14]

a) Δ*E* (*n*+1) < Δ*E* (*n*) monotonous bathochromic shift
b) Δ*E* (*n*+1) > Δ*E* (*n*) monotonous hypsochromic shift
c) Δ*E* (*n*+1) ≈ Δ*E* (*n*) borderline case between a) and b)
a) Δ*E* goes through a minimum for a certain *n*

The fifth case, in which Δ*E* passes a maximum for a special *n* is still unknown.

The series **111t-v** belongs to category d) [308]. The decrease of the ICT with increasing conjugation length provokes a hypsochromic effect which cannot be compensated for *n* = 3 by the bathochromic effect caused by the extension of the conjugated system. This result is in contrast to the analogous stars with terminal alkoxy groups **111n-q**, which belong to category a) [307]. Alkoxy groups are weaker electron donors than dialkylamino groups; therefore the extension of the chromophores can overcompensate the decrease of the ICT.

The absorption of **111t-v** depends unusually strong on the concentration. This deviation from Lambert-Beer law is attributed to a pronounced aggregation even at concentrations as low as 5 × 10^-5^ M. The J-aggregates formed absorb with higher *λ*_max_ and lower *ε*.

An important point concerns the influence of protic media. An addition of trifluoroacetic acid to solutions of alkoxy-substituted stars **111n-q** causes bathochromic shifts of about 82-93 nm of the absorption maximum due to protonation on the central triazine ring and enhanced ICT [306].

Protonation of amino groups should lead to a disappearance of their donor character, whereas protonation of the 1,3,5-trazine ring enhances the acceptor capability. Thus, protonation can strengthen or weaken the push-pull effect-depending on the preferred site. The protonation behavior of tris(dialkylaminostyryl)-triazines **111t-v** has been investigated by NMR spectroscopy: A preservation of *C*_3_ symmetry indicates a fast exchange of protons.

Star **111t** is first protonated on the 1,3,5-triazine ring resulting in a strong bathochromic shift of the absorption maximum (CH_2_Cl_2_: *λ*_max_ = 564 nm, **111t** /TFA = 1 / 2.8: *λ*_max_ = 564 nm). A large excess of trifluoroacetic acid turns the violet solution colorless with a *λ*_max_ = 373 nm. The higher homologue **111u** behaves totally different: The first protonation occurs at the amino groups and causes a hypsochromic shift (*λ*_max_ = 394 nm), and a high excess of TFA gives rise to a strong band with *λ*_max_ = 451 nm. **111v** behaves similar, a hypsochromic shift (*λ*_max_ = 412 nm) is followed by a bathochromic shift (*λ*_max_ = 485 nm) upon further protonation. Indications for aggregation of protonated **111t** have been found.

Theoretical studies (*ab initio* coupled Hartree-Fock) on 1,3,5-triazine and 2,4,6-trivinyl-1,3,5-triazine **113** resulted in second-order polarizabilities of |*β*| = 64.7 × 10^-32^ esu for *s*-triazine and |*β*| = 2110.1 × 10^-32^ esu for **113**. Similarly, the extension of the conjugated system results in a strong enhancement of the third-order polarizability from <*γ*> = 2304.5 × 10^-39^ esu for *s*-triazine to <*γ*> = 29,381.7 × 10^-39^ esu for **113** [309] A*b initio* calculations (HF/6-31G) [287] of a series of donor-substituted 2,4,6-triphenyl- (**98**), 2,4,6-tristyryl- (**111a**) and 2,4,6-tris(phenylbutadienyl)-1,3,5-triazines (**114**) revealed that the first hyperpolarizabilities increases with the conjugation length, probably because the electronic charge becomes more delocalised and the HOMO-LUMO energy gap and the bond length alternation decrease with variation of the chromophore structure. Also, the susceptibility of *β* to the donor strength (e.g., methyl, methoxy, dimethylamino) is found to be larger for the more elongated substrates.

The second harmonic generation of 2,4,6-tristyryl-1,3,5-triazine was studied by Fang [310]. **111a** in acetonitrile absorbs with *λ*_max_ = 322 nm, no absorption above 370 nm was observed. Upon irradiation with a laser beam (*λ* = 1064 nm) a SHG signal was detected with an efficiency 1.8 times higher than that of urea.

According to a X-ray analysis, the molecule adopts the shape of a slightly curved planar triangular kite with a molecular three-fold rotation symmetry. The rings are essentially planar, but all vinylene linkages are disordered. Nearly all bond lengths are between that of typical C-C or C-N single and C = C or C = N double bonds. A non-centrosymmetric packing of four molecules in the unit cell is believed to be the main origin of optical nonlinearity.

According to PM3 calculations, tris-(*p*-diethylaminostyry)-1,3,5-triazine **111s** takes a planar *C*_3h_ conformation [311]. The absorption maximum in CHCl_3_ appears at *λ* = 438 nm, the emission maximum at *λ*^F^ = 518 nm. Comparing this star with compounds having only one or two arms, absorption and emission maxima shift about 18 nm to the red with increasing number of arms. Upon irradiation with *λ* = 1064 nm, no SHG effects were detectable. Two-photon absorption and two-photon excited fluorescence were studied at the excitation wavelength of 800 nm. TPEF is similar to SPEF and the two-photon absorption cross-section at 800 nm amounts to 671 GM. The structurally related 1,3,5-tricyano-2,4,6-tris-(4-aminostyryl)benzenes [312] show two-photon absorption maxima in the range of *λ*^TPA^_max_ = 840-990 nm with TPA cross-sections around 1400 GM at *λ*^TPA^_max._

The absorption maximum of 2,4,6-tris-(4-dimethylaminostyryl)-1,3,5-triazine **111r** in CHCl_3_ appears at *λ*_max_ = 426 nm and the fluorescence at *λ*^F^_max_ = 511 nm (*Φ* = 0.066) [313]. In THF, a negative solvatochromism shifts the absorption to *λ*_max_ = 418 nm, but the fluorescence is bathochromically shifted to *λ*^F^_max_ = 516 nm (*Φ* = 0.033) [314]. Two-photon absorption cross-sections of *σ* = 534 GM in CHCl_3_ [313] and of *σ* = 2405 GM in THF at *λ*^exc^ = 800 nm [314] were reported. The analogous star with piperidine end groups **111w** shows a *σ* = 2523 GM in THF. Comparing triazines with one, two or three aminostyryl branches, the increase of the extinction coefficients and the TPA cross-sections is about 1:2.6: 4 and 1:3.5: 7 indicating a large cooperative enhancement relative to the number of branches 1:2: 3.

*Trialkynyltriazines (C-9-A-2 and C-9-A-8):* the synthesis of trialkynyl-1,3,5-triazines 115 (C-9-A-2) and 116 (C-9-A-8) appears to be a special challenge. These stars are generally prepared from lithium acetylides 117 and require cyanuric fluoride 101b as coupling partner. Nevertheless, only moderate yields [315] were obtained. The palladium-catalyzed coupling of alkynylstannanes 118, introduced by Faust [316], allows the use of cyanuric chloride 101a and gives similar yields.

**Scheme 32:**
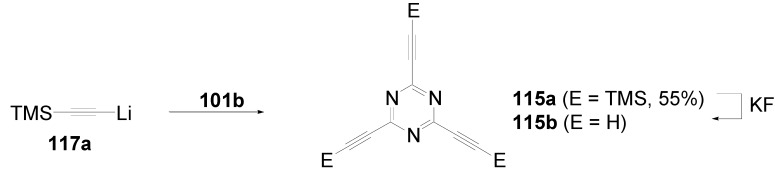
Synthesis of triethynyl-1,3,5-triazine **115b** via nucleophilic substitution.

By reaction of cyanuric fluoride **101b** with lithium trimethylsilylacetylide **117a**, a highly soluble triyne **115a** was isolated in 55% yield. X-ray structure determination of this compound shows parallel sheets of individual planar molecules with small distorsions of the triazine ring due to packing effects [315]. Desilylation results in 2,4,6-triethynyl-1,3,5-triazine **115b** [317]. Sublimed **115b** crystallizes in a layered structure. The triethynyltriazine molecules are connected by self-complimentary hydrogen bonding. All triazine-*N* atoms participate in short and linear CH-*N* contacts with a neighboring alkyne forming a unique two-dimensional hexagonal structure. The interplanar separation of 3.23 Ǻ is shorter than that of graphite (3.4 Ǻ) and therefore, there would be significant π-stacking interaction between the layers of **115b**.

**Scheme 33:**
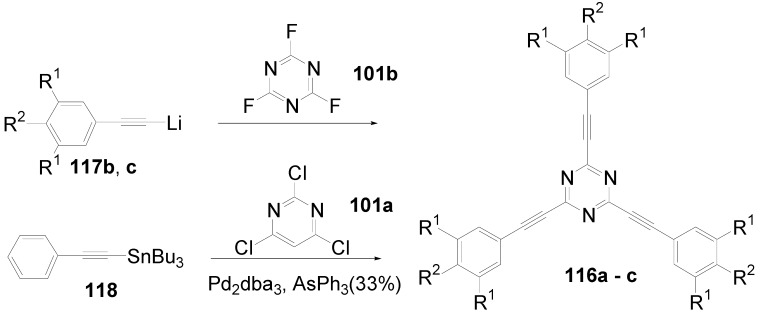
Synthesis of 2,4,6-tris(phenylethynyl)-1,3,5-triazines.

A tris(phenylethynyl)1,3,5-triazine with three dodecyloxy groups on the 3,4,5-positions of each benzene ring **116a** has been prepared by Meijer [318] in 23% yield from the lithio salt of the phenylacetylene **117b** and **101b** (Scheme 33). This star shows a LC phase between 22.9 °C and 60.3 °C (DSC and POM). The X-ray diffraction pattern was unlike those seen for other discotic molecules, but an exact determination was not possible. Bathochromic shifts of the excitation (*λ*_max_ = 358 nm in hexane, *λ*_max_ = 370 nm in CHCl_3_) and fluorescence spectra (*λ*^F^_max_ = 399 nm in hexane, *λ*^F^_max_ = 514 nm in CHCl_3_) with increasing solvent polarity indicate a strong charge transfer in the excited state.

Similarly, tris-(4-diethylaminophenylethynyl)-1,3,5-triazine **116b** (R^1^ = H, R^2^ = N(C_2_H_5_)_2_) was successfully prepared by Wolff [280] from lithium *N*,*N*-diethylaminophenyl acetylide **117c** and **101b** in 37% yield. Compared to the tris(diethylaminophenyl)-1,3,5-triazine **98k** , the absorption maximum is shifted about 50 nm to longer wavelengths (*λ*_max_ = 425 nm in acetone)

The elongation of the π-system of **98k** by an acetylene unit to **116b** also increased the large second-order polarizability to *β* = 404 × 10^-50^ Cm^3^V^-2^ (hyper Rayleigh scattering), 2.5 times higher than the *β* value found for the smaller analogue **98k**.

*2,4,6-Trisstilbazolium-1,3,5-triazines (C9-A-7´):* A unique series of π-conjugated stars **119a**-**c** has been reported by Cherioux. The triazine core is connected to three cationic stilbazolium arms with electron releasing groups in the periphery. The nucleophilic substitution on cyanuric chloride with donor-substituted stilbazoles **120a**-**c** (2h, 80 °C, ethyl acetate) gave the tricationic stars in excellent yields (>90%) [319].

**Scheme 34:**
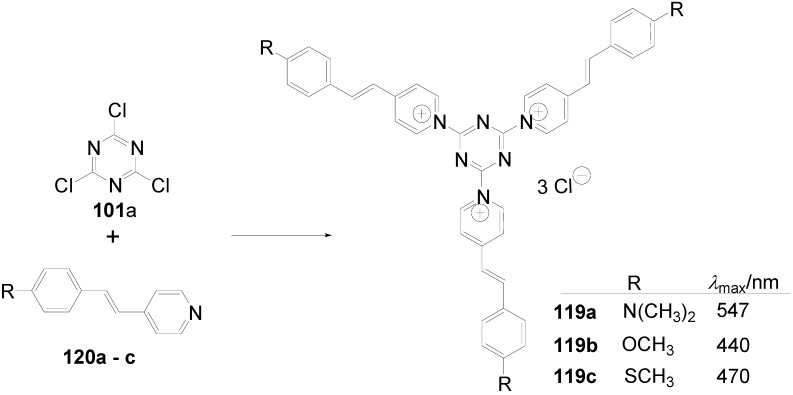
Synthesis of trisstilbazolium stars.

The charge on the nitrogen atoms is extremely delocalized. The only band in the UV-vis spectra of these tris-stilbazolium ions peaks at *λ*_max_ = 440 nm (**119b**), 470 nm (**119c**), or 547 nm (**119a**) with extinction coefficients in the range of 2.8 × 10^8^ L mol^-1^ cm^-1^. Like the UV-vis absorption, the first reduction potential is strongly controlled by the peripheral groups: from-0.485 V (*vs.* SCE) for **119c** to-0.71 V for **119a** [319].

NLO measurements have been performed via harmonic light scattering (HLS). The nonlinearities obtained are slightly higher than those of the classical *p*-nitroaniline with √<*β*^2^> = 5 × 10^-30^ esu. The highest value was obtained for the star with methylthio end groups **119c** √<*β*^2^> = 45.6 × 10^-30^ esu which can be readily accounted for by a more favourable charge transfer by the stronger donor end group [320]. In the case of the dimethyamino star **119a** the harmonic wavelength signal was buried within a broad two-photon emission peak around 650 nm (irradiation wavelength 1,34µm). In this case, the HLS signal could not be discrimitated from the two-photon fluorescence background. Contrary to the dimethyamino dye **119a**, stilbazolium stars with less electron-releasing donor groups **119b**, **c** did not present any two-photon fluorescence.

*Melamines (C-9-N-A-3):* Stars with a 2,4,6-triamino-1,3,5-triazine core **121** are generally prepared by nucleophilic substitution of the chlorine atoms of cyanuric chloride **101a** with anilines **122**.

**Scheme 35:**
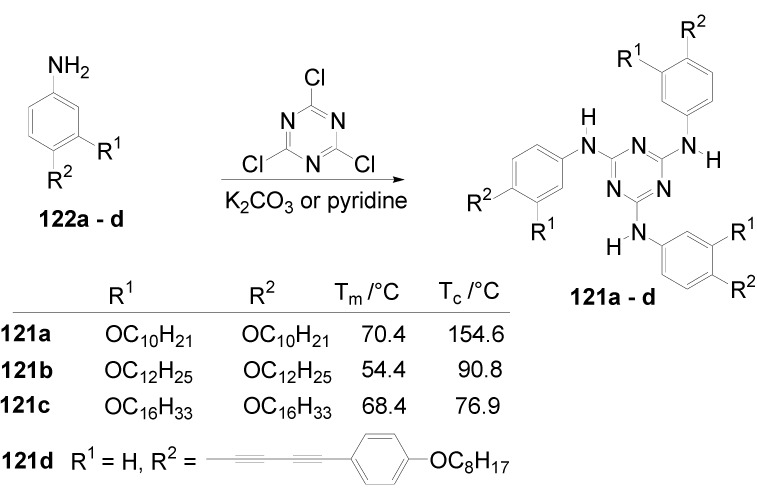
Synthesis of alkoxyphenyl- and alkoxytolanylmelamines.

These melamines, if substituted with three dialkoxyphenyl groups [321], exhibit enantiomorphic mesophases irrespective of the length of the peripheral side chains. Fan-like structures were observed by POM for **121a** and **121b** whereas the compound with hexadecyl side chains **121c** exhibited a broken, mottled structure. A reluctance to recrystallize was observed for all compounds. The clearing temperatures decrease as a function of the increasing length of the side chains. X-ray scattering reveals disordered hexagonal columnar structures for **121, b** substituted compounds but an ordered rectangular columnar phase of the hexadecyl substituted **121c.**

**Table 8:** Phase transition temperatures of tris-3,4-dialkoxyarylmelamines **121a-c** and their 1/1-mixtures with non-mesomorphic compounds. **123a**: 3,5-didexyloxy benzoic acid, **123b**: 3,5- didodexyloxy benzoic acid, **123c**: 3,5-dihexadexyloxy benzoic acid; **123d**: 3,4-didodexyloxy benzoic acid, **124**: 2,4,7-trinitrofluorenone, **125**: (2,4,7-trinitrofluorenyliden)malodinitrile.

Star	2. compound	T_m_ /°C	T_c_ /°C	Structure
**121a**		70.4	154.6	Col_hd_
**121b**		54.4	90.8	D_hd_
**121c**		68.4	76.9	D_ro_
**121a**	**123a**	32.4	64.8	Col_hd_
**121a**	**123b**	34.5	75.1	Col_hd_
**121a**	**123c**	18.7	73.6	Col_hd_
**121a**	**123d**	62.2	88.7	Col_rd_
**121a**	**124**	68.1	110.4	SmA
**121b**	**124**	68.6	115.9	SmA
**121b**	**125**	93.7	147.2	Col_r_

These compounds form monolayers on the air/water interface. The surface pressure-area isotherms show collapse ranges between 1.15 and 1.25 nm^2^ per molecule, the area does not depend on the length of the peripheral side chains. The central melamine ring lies flat on the water surface and the peripheral side chains are oriented perpendicular to the water surface

A mixture of tris(3,4-didecyloxyphenyl-1-amino)-1,3,5-triazine **121a** and 3,5-dihexadecyloxybenzoic acid **123c** showed transition temperatures at 18.7 (cryst. to mesophase) and 73.6 °C (mesophase to isotropic). X-ray diffraction reveals disordered hexagonal columnar structures [322]. This combination of two principles of structure formation, form anisotropy and intermolecular hydrogen bonding allows a control of the formation of columnar liquid crystalline structures.

Sixfold alkoxy-substituted 2,4,6-triarylamino-1,3,5-triazines **121a-c** are “open-sided” core systems that have the capacity to form columnar mesophases of the single components and also allow the docking of a second component to a molecular recognition site located in the inner core region [323]. Mesomorphous aggregates from **121a-c** as electron donors and non-mesogenic acceptors 2,4,7-trinitrofluoren-9-one **124** form enantiotropic mesophases between 68 °C and 115 °C with smectic A structure. Both transitions are shifted to higher temperatures when (2,4,7-trinitrofluorenyliden)malodinitrile **125** was used as acceptor. Additionally, the structure of the mesophase changed to rectangular columnar.

Even nonmesomorphic 2,4,6-triarylamino-1,3,5-triazine **121e** (R^1^ = H, R^2^ = OC_12_H_25_) and TNF gave columnar as well as smectic liquid crystalline structures [324].

Polymerizable liquid crystal molecules are very useful in the preparation of anisotropic materials. The ordered array of mesogenic monomers in the LC state can be fixed by polymerization which results in stable polymeric materials with a two or three dimensional order. Diphenyldiacetylene **125** with an octyloxy chain and an amino group in the peripheral positions was condensed with **101a** in the presence of potassium carbonate to give the star **121d** composed of a melamine core with three rigid diphenyldiacetylene branches and octyloxy side chains [325]. **121d** forms a liquid crystalline phase with a crystal to crystal transition at 189 °C followed by a transition into a hexagonal columnar mesophase at 197 °C. Further heating resulted in thermal polymerization at 280 °C. When the LC phase was quenched to room temperature, the texture was maintained. UV irradiation in the LC state resulted in a polymer with an ordered structure.

Similar compounds without alkoxy side chains or the diphenyldiacetylene connected via ether linkages to the triazine did not show mesophases. For the formation of mesophases, the outer benzene rings appeared to be essential. Related compounds **121e**-**g** with different alkyl chains replacing the peripheral alkoxyphenyl groups, obtained by base-induced condensation of **101a** with the respective anilines, did not show a thermotropic LC-behavior. Mixed with **124** in a 1:1 molar ratio, the second DSC heating and cooling curves gave only a mesophase-isotropic transition at 121 °C (**121e**: R^2^ = decadi-1,3-ynyl), 130 °C (**121f**: R^2^ = dodeca-1,3-diynyl), and 137 °C (**121g**: R^2^ = tetradeca-1,3-diynyl) [326]. POM shows the typical textures of columnar mesophases in the LC state that was maintained even at room temperature. X-ray scattering reveals a long-range ordering of columns with an intercolumnar spacing of 35.3 Ǻ (**121f**). UV irradiation in the LC state (125 °C) of **121f** resulted in oligomerization through the diacetylene units. The oligomers still show LC behavior similar to the monomers but with a much higher viscosity of the LC state.

### 5.5. Borazine as a Core

Borazine, a carbon-free aromatic heterocycle has been used by Yamaguchi [327] as a core connecting six aryl groups. The synthesis of these molecules **126** with a threefold symmetry axis starts with a cyclocondensation of butyl amine or *p*-substituted anilines and boron trichloride to *B*,*B*´,*B*´´-trichloro-*N*,*N*´,*N*´´-tributyltriazine **127a** and the *N*,*N*´,*N*´´-triaryl derivative **127b-d** followed by the threefold substitution of chlorine using 9-lithioanthracene **128a, b**. The yields of this one-pot procedure are generally high, up to 70%. Dihedral angles between the borazine plane and the aryl substituents are about 72°-76° giving the molecules a gear-shaped motif.

Relative to anthracene, the fluorescence spectrum of **126b** is shifted about 17 nm to the red and the fluorescence intensity (*Φ* = 0.62) increases by a factor of two. This increase in quantum yield was attributed to the sterically congested bundle preventing conformational disorder leading to nonradiative decay. The interaction of the aromatic groups is also responsible for a shift of the oxidation potential to less positive potential (E_pa_ = 0.94 V for **126a** (trianthryl-tributyl) and E_pa_ = 0.85 V for **126b** (trianthryl-tri-*p*-hexylphenyl).

**Scheme 36:**
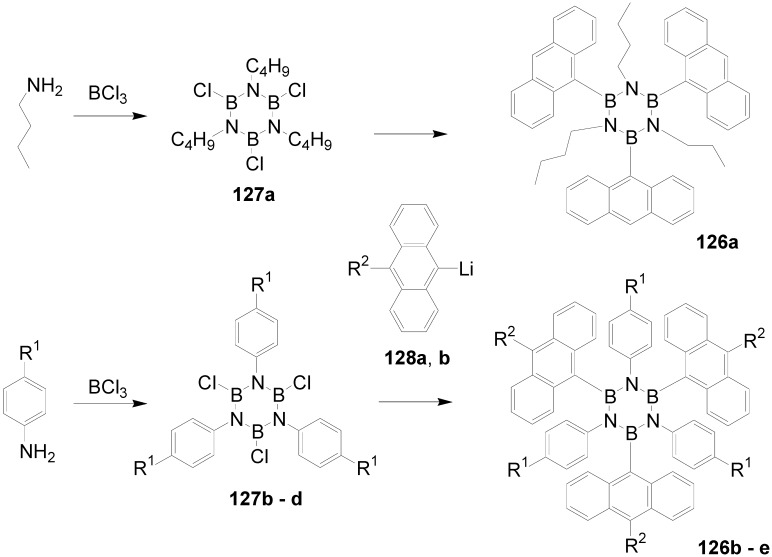
Synthesis of hexasubstituted borazines **126a-e**.

Several tri-9-anthryl-tri-*p*-hexylphenyl-borazines (**126f**: R^2^ = *p*-diphenylaminophenyl, **126g**: R^2^ = diisopropylsilyl, **126h**: R^2^ = and dimesitylboryl) were prepared via lithiation of **125e** (R^2^ = Br). Notably, the boryl-substituted derivative **126h** shows three reversible reduction waves between-2.29 and-2.52 V (*versus* ferrocene) and a reversible oxidation wave at + 0.72 V. Since the first reduction potential is comparable to that of Alq_3_ (-2.36 V), this material indicates a potential as electron transporting material in OLEDs. On the other hand, diphenylaminophenyl-substituted **126f** shows three oxidation waves (E_pa_ = 0.60, 0.80, 1.03 V). These data are promising for the application of trianthrylborazines as active materials in electronic devices.

## 6. Condensed Ring Systems as Cores

Condensed rings systems are common anisotropic cores in the area of liquid crystals inducing columnar mesophases [328,329]. Their sound investigation started at the end of the last century when columnar liquid crystals and their high charge carrier mobilities compared to disordered conjugated polymers were discovered [328]. With respect to star-shaped molecules with conjugated arms we focus here on one of the smallest systems-the triphenylenes (C-12) their heterocyclic counterparts (C-13), as well as triazatruxenes (C-14) and tristriazolotriazines (C-15). For these materials the variation of the supramolecular and optoelectronic properties by attachment of conjugated arms is highly attractive and consequently will be subject of the next sections.

### 6.1. Triphenylene Star Compounds (C-12)

#### 6.1.1. Structures and Synthesis

**Figure 30:**
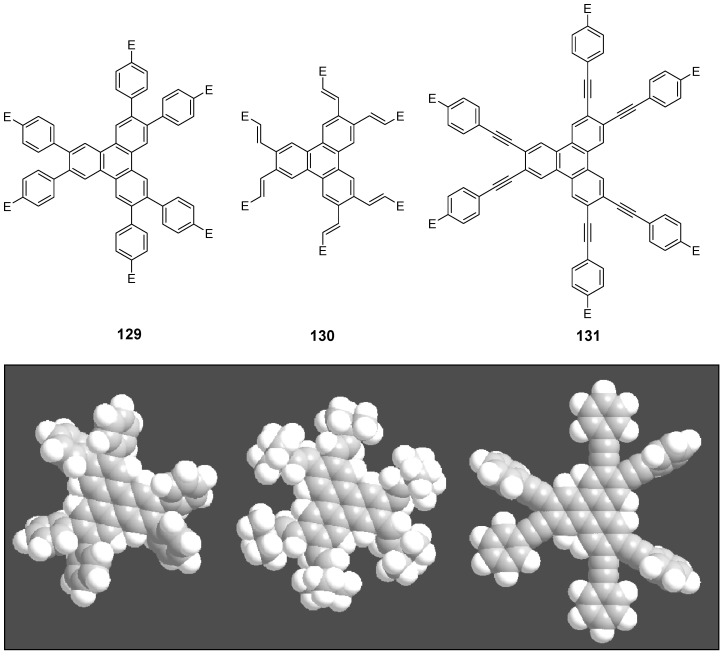
Parent structures of triphenylene stars (C-12-A-3 (**129**), C12-A1 (**130**), C-12-A-8 (**131**)) and their CPK models.

Figure 30 summarizes the reported parent star-shaped triphenylene structures with conjugated arm scaffolds (C-12-A-3 (**129**), C12-A1 (**130**), C-12-A-8 (**131**)) and illustrates their space filling models. Steric demands force the arm scaffolds of molecules **129-131** out of plane with respect to the triphenylene core, analogous to structures found for the three-, four- or six- arm stars with a benzene core (see chapter 4). However, even the hydrogens in the bay position may result in a twist of the triphenylene disk itself by several degrees, which was revealed from single crystal structure analysis [330]. Crystal structures are not known for compounds **129** and **131**. The structure analysis of a single crystal of **130a** reveals a statistical rotation of the double bonds out of plane of the core [209]. However, the packing of the molecule in the crystal demands also an almost coplanar arrangement of two *tert*-butylethenyl units. The spatial arrangement of the arms dominates the supramolecular properties of this molecular family as will be discussed in the subsequent section.

**Scheme 37:**
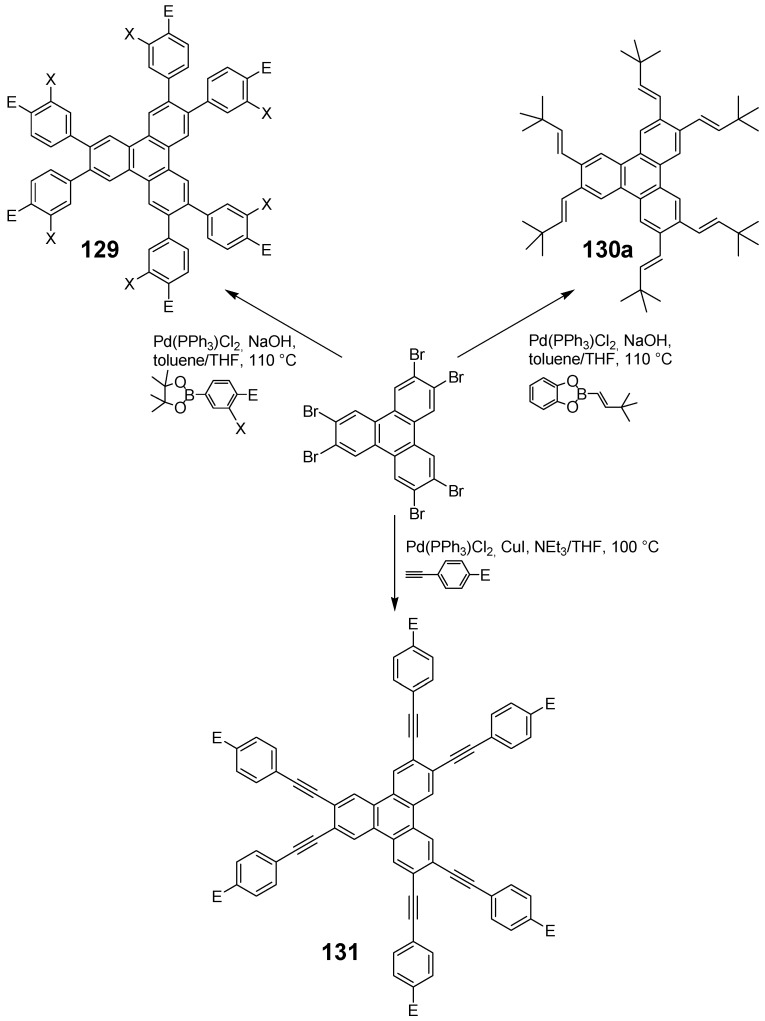
Synthesis of triphenylene stars. Suzuki (compounds **129**, **130**) and Hagihara-Sonogashira (compound **131**) reactions had to be optimized for efficient sixfold couplings.

The synthesis of compounds **129**-**131** is illustrated in Scheme 37. Although many synthetic strategies allow the preparation of symmetrically substituted triphenylenes [331], all members of this molecular family were obtained in a semi divergent/convergent approach [209,211,234,332]. In all cases 2,3,6,7,10,11-hexabromotriphenylene was converted in a convenient transition metal catalyzed cross-coupling reaction, in which six arms were attached. A yield of 93%which is reported for the synthesis of **130a** implies that each single coupling step require a conversion of 98.8%[209].

#### 6.1.2. Triphenylene Derivatives and Materials Science

*Oligophenylene Substituted Triphenylenes (C-12-A-3):* Oligophenylene substituted triphenylenes **129** are the most frequently studied systems, due to their high stability and shape persistence. The latter is a feature of interest in the design of coordination networks, which has been attempted for **129a** (E = SCH_3_) with BiBr_3_ [171] and AgOTf. [333] Only the latter salt resulted in the formation of a 3D network.

**Figure 31:**
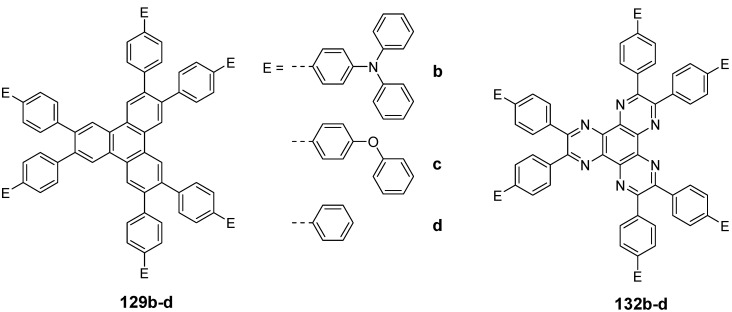
Extented triphenylene stars showing efficient energy transfer in mixtures.

Optoelectronic properties has been studied for triphenylenes **129b**-**d** and hexaazatriphenylenes **132b**-**d** [334]. Absorption and fluorescence maxima at long wave length of **129b** are bathochromically shifted (356 nm, 445 nm) compared with triphenylene derivatives. The absorption maximum of the donor-acceptor compound **132b** at 421 nm overlaps considerably with the fluorescence spectrum of **129b**, which results in an efficient energy transfer. The blue emission of **129b** is quantitatively quenched in an equimolar mixture and only the green emission of **132b** with a maximum at 543 nm is observed.

Evidently the most comprehensive work has been done in the area of columnar liquid crystals as semiconducting materials for optoelectronic devices. Table 9 collects all the different reported hexaphenyltriphenylene derivatives and their mesomorphic behavior. Interestingly only the dodeca alkoxy substituted derivatives **129e**-**h** revealed columnar mesophases [332,335,336]. When these mesogens were converted to extended condensed triphenylenes by an oxidative cyclisation (Scholl reaction) [332,335], the resulting planar compounds showed a much broader LC temperature range than the parent non planar structures. The other compounds **129i**-**n** with only six flexible peripheral chains did not show any mesomorphism [332,337]. However most of these compounds exhibited a remarkable behavior when mixed 1:1 with the triphenylenes with six flexible chains [336,337,338,339,340]. For example, compound **129m** formed with hexahexyloxytriphenylene **133a** (Cr 70 °C Col_h_ 100 °C I) in a 1:1 mixture a hexagonal columnar mesophase from 66 °C to 155 °C. This new mesophase was not miscible neither with an excess of triphenylene **133a** nor with **129m**. The enormously stable 1:1 aggregates could not be explained by simple quadrupole interaction, but were rationalized by complementary polytopic interactions (CPI), which is the sum of the atom centered van der Waals and multipole interactions between the two components [338,341]. The hexaphenyltriphenylenes form a cavity with the twisted phenyl groups and can be regarded as supramolecular hosts for the smaller planar triphenylenes. This interaction is even enforced when the electron poor hexaazatriphenylene host **132a** is applied (see last entry Table 9). A library of different triphenylenes **133** with alkoxy, oligoethylenoxy and chains with ester connecting groups has been tested. The temperature range of liquid crystal phases has either been extended for triphenylenes **133** or mesophases have been induced for non-mesomorphic derivatives. Not any new, stabilized liquid crystal phase revealed, when the aggregate formation was prevented by steric demanding groups such as in hexaphenyltriphenylenes **129e-h**, **129l** or in mixtures with the 2,3,6,7,10,11-hexahexyloxy-1-nitrotriphenylene [337,338]. The columnar mesophases created by CPI exhibited a remarkable high order [336], an enhanced charge carrier mobility [342,343,344,345,346] and a rather low temperature dependence of the latter [347,348]. The charge carrier mobilities increased from 7.1 × 10^-4^ cm^2^V^-1^s^-1^ (353 K) for **133a** by almost two orders of magnitude to 2.3 × 10^-2^ cm^2^V^-1^s^-1^ (393 K) for the CPI mixture **133a** with **129m** [342]. The weak temperature dependence was rationalized by the Holstein small polaron model in the non adiabatic limit [347,348]. Note that the best charge carrier mobility in a liquid crystal was found for a perylene derivative and amounts to 1.3 cm^2^V^-1^s^-1^ [328,349]. Charge carrier mobilities of up to 15.9 cm^2^V^-1^s^-1^ have been predicted based on Marcus theory (hopping transport) for perfect structures of small graphenes [350].

**Table 9:** Mesomorphic properties of triphenylenes **129**.

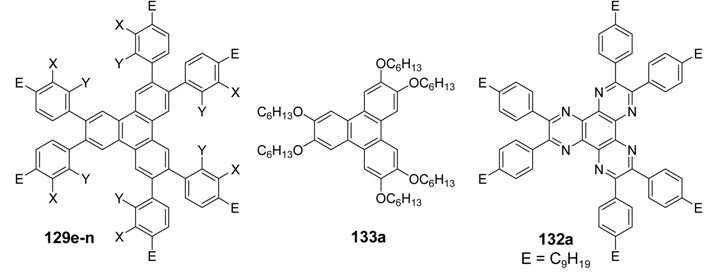					
Compound	E	X	Y	phase transitions [°C]	reference
**129e**	OC_6_H_13_	OC_6_H_13_	H	Cr 111 Col 126 I Cr 65 Col_h_ 135 I	[335,336]
**129f**	OC_8_H_17_	OC_8_H_17_	H	Cr 85 Col 104 I	[335]
**129g**	OC_10_H_21_	OC_10_H_21_	H	Cr 74 Col 103 I	[335]
**129h**	OC_12_H_25_	OC_12_H_25_	H	Cr 47 Col 101 I	[335]
**129i**	OC_6_H_13_	H	H	Cr 153 I	[332]
**129j**	OC_11_H_23_	H	H	Cr 66 I	[332]
**129k**	H	OC_6_H_13_	H	Cr 11 I	[337]
**129l**	H	H	OC_6_H_13_	Cr 81 I	[337]
**129m**	C_9_H_19_	H	H	Cr 59 I	[332]
**129n**	C_12_H_25_	H	H	Cr 37 I	[332]
**129m + 133a** 1 :1				Cr 66 Col_h_ 155 I	[336]
**132a**	C_9_H_19_			Cr 81 I	[336,338]
**132a + 133a** 1:1				Col_h_ 240 I	[336,338]

*Oligoethenylene and Oligo(phenylenevinylene) Substituted Triphenylenes (C-12-A-1 and C-12-A-6):* there are no reports on oligoethenylene derivatives, but only on the hexa(*tert*-butylethenyl)triphenylene **130a** of Meijere *et al.* [209]. This type of compound was suggested to be useful as ligand or supramolecular host. Oligo(phenylenevinylene) derivatives of triphenylenens were only considered theoretically [351]. For the octupolar donor or acceptor end-capped 2,6,10-trisoligo(phenylenevinylene)triphenylenes a high two-photon absorption was predicted.

**Figure 32:**
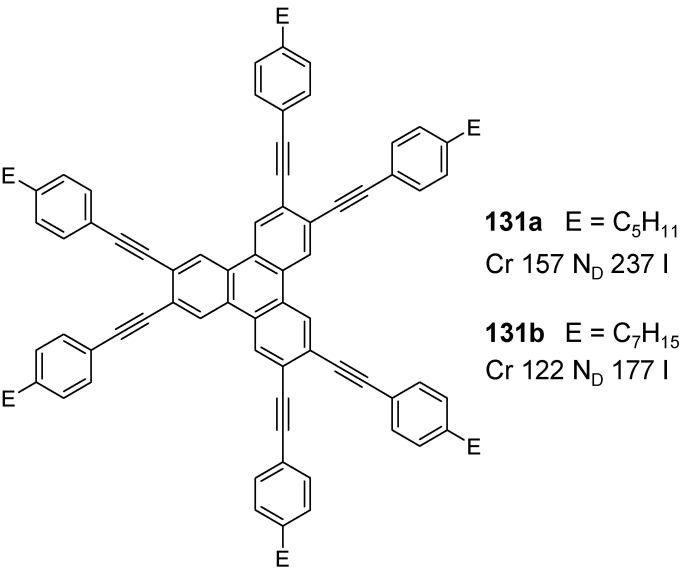
Praefkes multiyne nematogens.

*Oligo(phenylene ethynylene)triphenylene (C-12-A-8):* Like triphenylenes with oligo(phenylenevinylene) arms the derivatives with oligo(phenyleneethynylene) arms were rarely studied. Huang *et al*. synthesized triphenylenes with 2-(4-ethynylphenyl)ethynyl arms [234]. Upon complexation with the transition metal entities the absorption and fluorescence spectra were bathochromocally shifted by a simultaneous decrease of the fluorescence quantum yield. Praefke *et al*. studied the 4-alkylphenylethynyl substituted triphenylenes **131a**,**b** (Figure 32), which they called the multiyne mesogens [211]. Without any exception they self-assembled in nematic mesophases, the mesophase with the lowest order, which can be rationalized considering the non planar structure in the periphery of the disk (cp. Figure 30) and the small fraction of flexible aliphatic chains. The mixture with dipole bearing groups and even chlorocyclohexane can induce a columnar mesophase which is stable up to 250 °C [352]. The authors suggested that the latter is induced owing to the filling of free space between the arms of the multiynes in the periphery.

### 6.2. Hexaazatriphenylenec (C-13-A-3 and C-13-A-6)

Dipyrazinoquinoxaline, the hexaaza analog of triphenylene, amalgamates the structural features of the hydrocarbon and the electron affinity of the pyrazine ring. The general synthetic approach is based on the construction of the three pyrazine units, reports about functionalization of existing triazatriphenylenes are only sparse [353,354]. Praefcke [355] developed the most important route to hexaazatriphenylenes:the threefold condensation of 1,2-diketones **134** with hexaaminobenzene **135**. Compounds with six peripheral (*p*-substituted) phenyl rings **132** were obtained in 14-68% yield. Upon irradiation into the absorption (*λ*_max_ = 396 nm) of the *p*-methoxy derivative**132f**, an intense emission with *λ*^F^_max_ = 461 nm was observed.

**Scheme 38:**
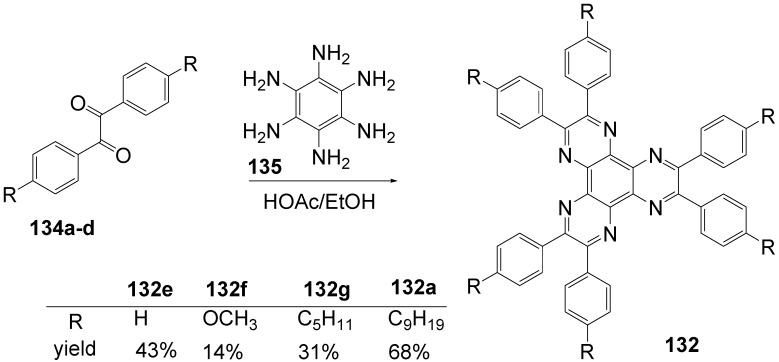
Synthesis of hexaazatriphenylenes **132** via threefold condensation of benzils with hexaaminobenzene.

Fages [356] could show that the condensation of hexaaminobenzene with three benzil units, connected via two pentaethyleneglycol tethers, allows the formation of *C*_2_–symmetrical hexaphenylhexaazatriphenylenes with macrocylic bridges **132h**. Hexaazatriphenylenes **132** are planar trifunctional chelating ligands for transition metals [357]. Substituted with six phenyl or six *p*-*t*-butylphenyl groups (**132e**, **132i**:R = C(CH_3_)_3_), hexaazatriphenylenes have been used by Lehn [358,359] in combination with bis-bipyridyl and Cu^+^ for the formation of supramolecular architectures of nanometric size.

Hexaphenyl substituted hexaazatriphenylenes with peripheral flexible side chains can form liquid-crystalline mesophases, as pure compounds or in mixtures with hexakis(hexyloxy)triphenylene. Bushby [336] reported that a 1:1 mixture of the triphenylene with hexakis(*p*-nonylphenyl)hexaazatriphenylene **132a** shows a mesophase between 130 °C and 240 °C with a hexagonal columnar structure. The LC behavior of triphenylene/hexaazatriphenylene mixtures is discussed in detail in chapter 6.1. **132a** shows semi reversible reduction above 1.80V and oxidation below –0.6 V (in benzonitrile, *vs.* Ag/AgCl) [360].

Hexaazatriphenylenes with six biphenyl arms and peripheral donor groups (**132c**:R = C_6_H_6_OC_6_H_5_; **132b** R = C_6_H_6_N(C_6_H_5_)_2_; **132j**:R = C_6_H_6_N(C_6_H_5_)-2-C_10_H_7_) were obtained from condensation of **135** and the substituted benzils **134f**-**g** [361,362,363]. These compounds, though missing any flexible side chain, form liquid-cystalline phases at high temperatures [361]. Hexagonal columnar phases between 343 °C and 385 °C for the diphenylamino derivative **132b**, 278-398 °C for **132c** and 357 °C-464 °C for the *N*-phenyl-*N*-2-naphthylamino derivative **132j** were found. The self-assembling of these molecules is also responsible for gelation of 10^-2^ M solutions of these stars in aniline or nitrobenzene.

**132b** forms an amorphous type solid preserving the one-dimensional ordering in a cooling process from the liquid crystalline state [361]. In spin-coated films of mixtures of this star and the analogous triphenylene, these compounds form an efficient energy transfer system due to a strong spectral overlap of the triphenylene emission (*λ*^F^_max_ ≈ 450 nm) and the long-wavelength absorption (*λ*´_max_ ≈ 420 nm) of the hexaaza derivative. With a decreasing ratio (100:1-0:100) of triphenylene donor / hexaazatriphenylene acceptor, the emission of spin-coated films gradually shifts from *λ*^F^_max_ = 500 nm to 543 nm.

The UV-vis absorption maximum of hexaphenyl substituted hexaazatriphenylenes **132** is shifted to longer wavelengths with the extension of the π-system (**132e**:*λ*_max_ = 366 nm, **132d**:*λ*_max_ = 392 nm) [363]. In combination with strong donors this lowered transition energy Δ*E* is overcompensated by a reduced ICT and a hypsochromic shift of the absorption band occurs (**132k**:*λ*_max_ = 453 nm, **132b**:*λ*_max_ = 417 nm). Whereas the UV-vis absorption is nearly unbiased by solvent polarity, the fluorescence of diphenylamino substituted **132b** is strongly solvatochromic. **132b** shows a remarkable solvatochromic shift of 39.344 cm^-1^ comparing solutions in toluene and dichloromethane, nearly twice as high as that of the lower homologue **132k**. The fluorescence quantum yields of **132** benefit from peripheral donor substitution but become sensitive towards solvent polarity. The fluorescence is characterized by a single exponential decay and solvent polarity reduces the compounds with an ICT.

**Table 10:** Optical data of substituted hexaphenylhexaazatriphenylenes **132**.

	R =	*λ*_max_ /nm (toluene)	*ε* / l/mol°cm	*λ*^F^_max_ / nm(toluene)	*Φ*	λ^F^_max_ / nm (CH_2_Cl_2_)	*Φ*
**132e**	H	366	21.000	419	0.01	424	0.03
**132k**	-N(C_6_H_5_)_2_	453	98.000	502	0.95	563	0.75
**132d**	-C_6_H_5_	392	68.000	438	0.43	449	0.50
**132b**	-C_6_H_4_- N(C_6_H_5_)_2_	417	129.000	501	0.96	624	0.27

**Figure 33:**
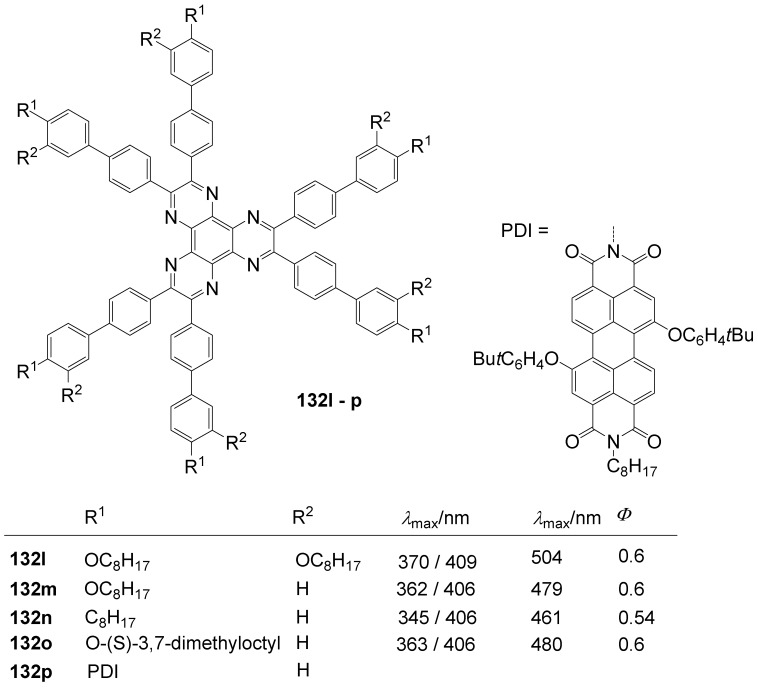
Hexakis(biphenylyl)-hexaazatriphenylenes **132l**-**p**.

Hexakis-(biphenylyl)-hexaazatriphenylenes with six or twelve flexible chains **132l**-**o** as well as stars with six perylenediimide end groups **132p** have been prepared and investigated as light-harvesting systems. Two strategies have been used:either the via sixfold Suzuki reactions of hexakis(*p*-bromophenyl)hexaazatriphenylene **132q** (R = Br) with *p*-substituted benzene boronic acids [353] or the threefold condensation of substituted bis(biphenylyl)diketones with hexaaminobenzene [364].

Compounds with six alkyl or alkoxy groups or twelve alkoxy groups provide two absorption bands around *λ* = 401 and 360 nm in chloroform. In cyclohexane solution and in the film state, self-assembling to H-aggregates occurs. The fluorescence maximum is sensitive towards peripheral donor substitution:millimolar solutions of the octyl derivative **132n** in CHCl_3_ emit with *λ*^F^_max_ = 461 nm, a *p*-alkoxy substitution (**132m**) shifts the emission about 18 nm to the red and with a sixfold 3,4-dioctyloxy substitution (**132l**), the emission peaks at 504 nm. In hexane, the fluorescence of the alkoxy derivatives is hypsochromically shifted, upon dilution to 0.1 and 0.01 mM, further shifts to the blue indicate deaggregation.

The hexaazatriphenylene **132p** with six biphenyl branches and perylenediimide end groups forms stable dimer aggregates that allow an efficient energy transfer from the azatriphenylene core to the peripheral perylene diimide units. The synthesis starts with a Suzuki coupling of a Boc-protected *p*-aminobenzeneboronic acid and **132r** followed by a condensation with a perylenedicarbox anhydride. The UV-vis spectrum of **132p** is dominated by the absorption of the perylendiimide unit (*λ* = 511 nm, 544 nm), the hexaazatriphenylene core gives a band at *λ* = 390 nm. Aggregation results in concentration and temperature depending absorption spectra. Irradiation into the absorption bands of the core or of the end groups results in an emission of the PDI acceptor moiety. Similarly, spin-coated films of mixtures of **132m** and **132p** show the PDI emission [353].

Hexastyryl substituted hexaazatriphenylenes **136a**, **b** were obtained by the condensation of unsaturated diketones **137a, b** and **135** [365]. With an increasing donor strength, the absorption maximum shifts to longer wavelengths (**132a**:*λ*_max_ = 460 nm; **132b**:*λ*_max_ = 526 nm (DMSO)) the hyperpolarizability increases from *β* = 18 × 10^-30^ esu to *β* = 50 × 10^-30^ esu. Upon threefold complexation with Cu(I)/phenanthroline, further red shifts of the absorption and increased nonlinearities of *β* = 31 × 10^-30^ esu and 197 × 10^-30^ esu were found resulting from a strengthened acceptor effect of the core.

**Scheme 39:**
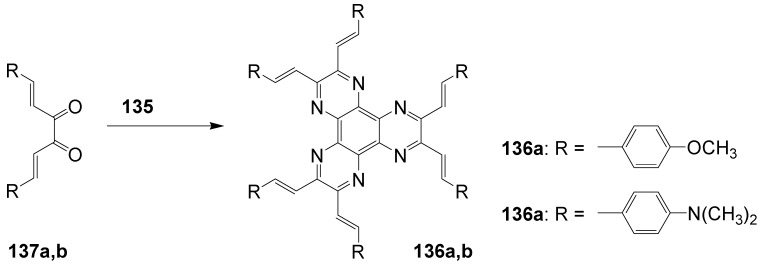
Hexakis(styryl)hexaazatriphenylenes.

Starburst compounds with a tribenzohexaazatriphenylene core **138** were investigated by Gao *et al*. [366]. The key step of the synthesis is a threefold condensation of 3,4-disubstituted *o*-phenylenediamines **139** with cyclohexanehexaone **140** (70-85%). The electronic spectra of the hexaphenyl substituted tribenzohexaazatriphenylene **138a** show *λ*_max_ = 413 nm and *λ*^F^_max_ = 432 nm (*Φ* = 0.23), extension of the conjugation (**138d**)as well as peripheral donor groups (**138b,c**) provoke significant bathochromic shifts of the absorption and, much more pronounced, of the emission. E.g. a sixfold triphenylamino substituted core **138c** absorbs at *λ* = 530 nm and fluoresces with *λ*^F^_max_ = 700 nm. The potential of the first reduction wave is nearly unbiased by the peripheral donor substituent, for **138a**:E_1/2_ = -0.81 and for **138c**:E_1/2_ = –0.77 (*vs.* Ag/AgCl).

**Scheme 40:**
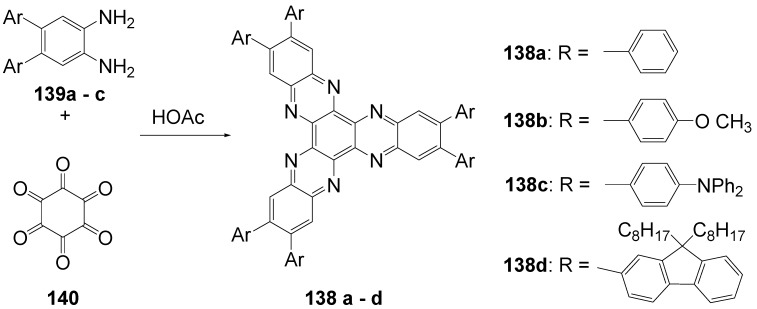
Synthesis of tribenzo-hexaazatriphenylenes.

### 6.3. Triazatruxenes (C-14-A-3 and C-14-A-8)

Carbazolyl groups have long been recognized in the construction of highly photoconductive amorphous materials. They undergo reversible oxidation processes and are able to transport positive charges via radical cation species. The triazatruxene (C-14) can be treated as an overlapping framework of three carbazole units and act as an electron donating unit for π-conjugated branches like oligofluorenes, improving hole injection and transport.

Hexabromotriazatruxene **141** [367] is the central starting material for triazatruxene based stars **142**, the attachment of conjugated arms is possible via Stille [368], Sonogashira [369] or Suzuki [370] coupling reactions.

**Scheme 41:**
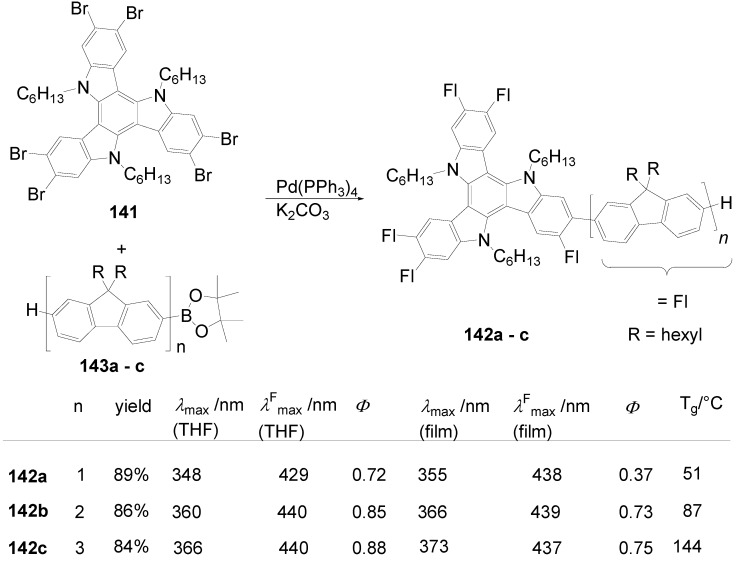
Synthesis of triazatruxenes with oligofluorene branches.

Six-armed stars with a triazatruxene core **142a**-**c** have successfully been prepared by microwave-assisted multiple Suzuki coupling reactions [371]. **141** was coupled with oligofluorenyl boronic acids **143a-c** to give the first, second and third generation in good yields (Scheme 41). With an increasing generation, the absorption maximum is gradually shifted to lower energies. In the fluorescence, a small red shift was recorded comparing the 1^st^ and the 2^nd^ generation, the latter represents the convergence limit. The stars form amorphous solids with glass transitions at 51 °C for the first and 144 °C for the third generation [372,373,374].

Blue light-emission from diodes with the configuration ITO/PEDOT:PSS/**142**/Ba/Al started at turn-on voltages of 4.0, 3.5, and 3.3 V with external quantum efficiencies of 0.24%, 1.35%, and 2.16% [368,373,374]. OLEDs with blends of this star in polyfluorene matrices showed an improved electroluminescence stability and external quantum yields close to 3% [375].

**Scheme 42:**
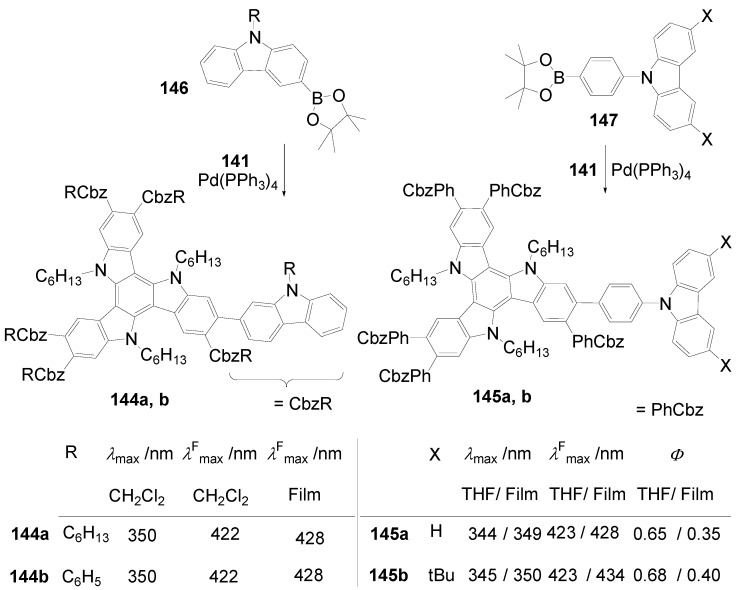
Hexacarbazolyl-triazatruxenes.

Similarly, six phenyl groups with a 9-carbazolyl substituent **145** have been attached to the triazatruxene [377] (Scheme 42).

Related stars with six 9-substituted carbazole moieties **144** (R = phenyl or hexyl) have been prepared from hexabromotriazatruxene **141** in about 50% yield [376]. The optical properties of these stars are nearly independent from the substituent on the 9-position of the carbazoles, the long-wavelength absorption maximum appears at *λ*_max_ = 350 nm and blue light (*λ*^F^_max_ = 422 nm) is emitted from solution in dichloromethane and from the solid material (*λ*^F^_max_ = 428 nm).

**Scheme 43:**
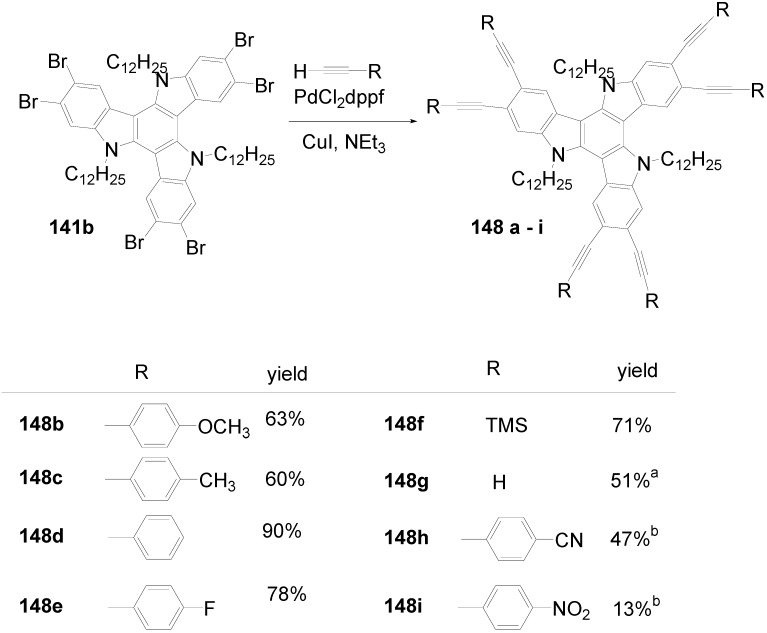
Hexaalkinyl-triazatruxenes. (a) Deprotection of the TMS-protected hexayne (b) Pd(PPh_3_)_4_ as catalyst.

**141** is also a suitable substrate for multiple Sonogashira couplings (Scheme 43). Stars with six octylethynyl **148a** as well as phenylethynyl groups with a variety of *p*-substituents **148b**-**e** could be obtained in 60-73% yield [369,378].

For a sixfold substitution with peripheral acceptor groups **148h**, **i** (R = CN, NO_2_), a Sonogashira reaction with TMS-acetylene followed by deprotection of **148f** and coupling of **148g** with iodobenzonitrile or iodonitrobenzene proved to be advantageous. In the electronic spectra, the absorption maximum is shifted from about *λ*_max_ = 320 nm of the hexakis-ethynyl star **148i** and *λ*_max_ = 350 nm for electroneutral **148c** or donor-substituted **148b** phenylethynyl derivatives to 420 nm for the compound with a *p*-nitro substitution**148g**. A significant aggregation in polar solvents was reported.

### 6.4. Tristriazolotriazines (C-15-A-3 and C-15-A-9)

The threefold annulation of 1,2,4-triazoles to a 1,3,5-triazine can result in two *C*_3_-symmetric tristriazolotriazines (TTT) **149**, **159**. Until recently, only two compounds containing the TTT core were known. Whereas the structure of the first reported TTT **149a** (R = NH_2_) remains ambiguous [379,380], Huisgens triphenyl-TTT has structure **150** (R = C_6_H_5_) [381]. Tristriazolotriazines **151** with 1,2,3-triazole rings are still unknown.

**Figure 34:**
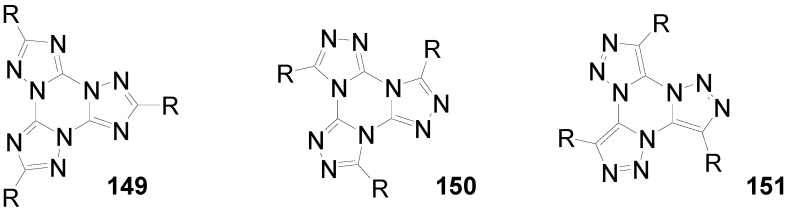
*C*_3_ **-**symmetrical tristriazolotriazines.

A triphenyl-TTT **149b** (R = C_6_H_5_) was obtained in 46% yield by thermal cyclocondensation of **152a** (R^1^ = R^2^ = H) at 230-270 °C. This procedure is also applicable to 3,5-dichloro-1,2,4-triazole [382] as well as to 3-chloro-4-hexyloxyphenyltriazole **152b** (39%) [383].

**Scheme 44:**
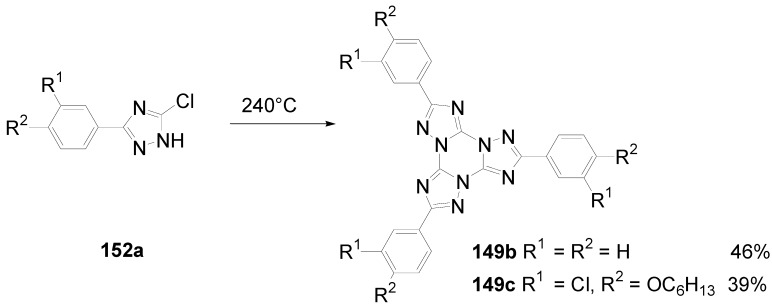
Cyclocondensation of 5-substituted 3-chloro-1,2,4-triazoles.

Tristriazolotriazines with peripheral alkoxy side chains have been prepared via the Huisgen route in reasonable yields [383,384]. A threefold nucleophilic substitution of the chlorine atoms in cyanuric chloride **101a** with **153** followed by ring opening of the tetrazoles with elimination of nitrogen and subsequent ring closure gives tristriazolotriazines of structure **150**.

**Scheme 45:**
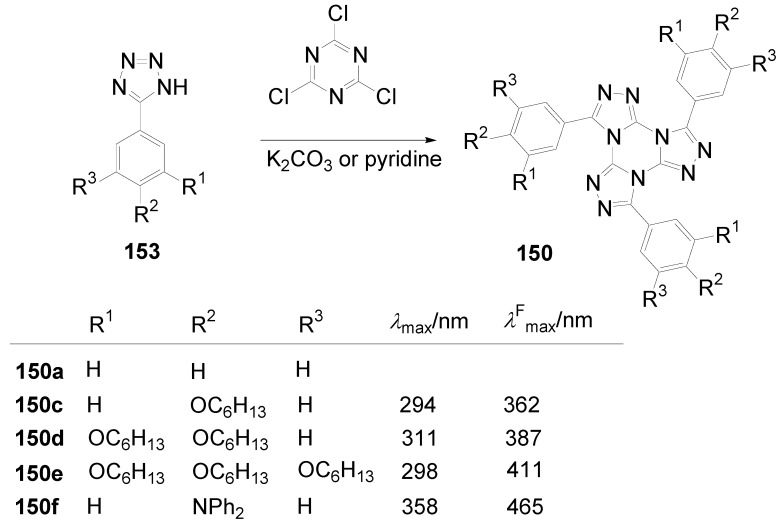
Synthesis of tristriazolotriazines **150** from tetrazoles **153**.

The X-ray structure [384] of the tris-*p*-methoxyphenyl derivative **150b** (R^1^ = R^3^ = H, R^2^ = OCH_3_) shows that the peripheral rings are in a non-planar conformation with respect to the triazine core. The mean plane angles for the twist of the three rings from the central ring are 19°, 29°, and 62°. Molecular packing is mainly due to van der Waals and dipole interactions.

Molecular π-stacking is sterically inhibited in the solid phase which improves the potential for these materials to be applied as good solid state emitters, since the formation of excimers via π-stacking is known to suppress the emission in solids. Alkoxy-substituted TTTs **150b**-**e** absorb in solution and in the solid state with *λ*_max_ in the range of 286-316 nm. In solution, an intense fluorescence (*Φ* = 0.25-0.47) with *λ*^F^_max_ between 360 and 387 nm is emitted. With an increasing number of alkoxy donors, absorption and emission maximum are shifted to lower energies and the fluorescence quantum yields increase. Whereas the absorption spectra are nearly unbiased by the solvent, the emission of donor-substituted triphenyl-TTTs is positive solvatochromic. Comparing solutions in cyclohexane and dichloromethane, shifts from *λ* = 381 nm to *λ* = 410 nm for **150e** and from *λ* = 412 to *λ* = 465 nm for the 4-diphenylamino derivative **150f** were observed [383].

Electrochemically, trimethoxy-TTT exhibited only an irreversible oxidation wave peaking at 1.6 V *vs.* SCE, suggesting that the tristriazolotriazine core potentially has electron-transporting characteristics [384]. The high band gap of these blue-emitting materials is advantageous for their application as hosts in electroluminescent devices.

The TTTs with a single alkoxy side chain in the 4-position of the benzene rings are non-mesomorphous, but they form stable molecular glasses with T_g_ = 55.4° (**150c**:R^2^ = O-hexyl), 46.7 °C (**150g**:R^2^ = O-octyl) and 70.3 °C (**1510**:R^2^ = O-dodecyl). This is a further evidence of non-aggregation in the solid state. But TTT **150i** with two dodecyloxy side chains forms a liquid crystalline phase between 92.2 °C and 207.6 °C with hexagonal columnar structure (POM and X-ray).

Since the cell parameter a was calculated to be 30.4 Ǻ, smaller than the van der Waals diameter of the molecule in the most extended conformation, either interdigitation or folding of the side chains occurs.

According to TGA, decomposition of TTTs starts only above 400 °C [384], but Tartakovsky reported a thermal rearrangement (350 °C, 30 min) of the triphenyl-TTT **150a** to an unsymmetrical isomer **154** [382].

**Scheme 46:**
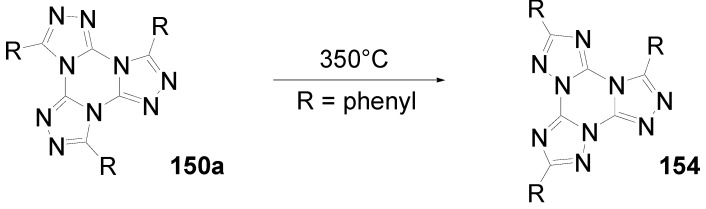
Isomerization of tristriazolotriazine **150a**.

Similarly, tristriazolotriazines with longer π-conjugated arms have been prepared. Stilbene [385] and tolane [383,386], substituted with alkoxy groups on the peripheral rings have been attached to the TTT core. Compared to the *p*-hexyloxyphenyl derivative **150c** (*λ*_max_ = 289, *λ*^F^_max_ = 347 in cyclohexane), the absorption and emission spectra of the TTT with tolane branches are shifted to lower energies (*λ*_max_ = 334, *λ*^F^_max_ = 372 in cyclohexane). Whereas the absorption of the tolane derivative is negative hypsochromic (Δ*λ* = 10 nm from cyclohexane to ethanol), the emission is characterized by a significant positive solvatochromism (Δ*λ* = 56 nm).

## 7. Summary and Conclusion

The present review reports on star-shaped conjugated compounds, monomers and oligomers, which consist of a cross-conjugated core and three, four or six linear conjugated arms.

After the introductory chapter 1, a general discussion of the molecular architecture and the conjugation effect of these compounds is presented in chapter 2. The uniform, monodisperse structures are essentially planar, have a relatively rigid shape-persistence and a well-defined size − even when numerous conformers exist.

The core of the here selected compounds is represented by single atoms (B, C^+^, N: chapter 3), benzene rings (chapter 4) or azine rings (pyridines, pyrimidines, pyrazines, 1,3,5-triazines: chapter 5). Additionally some polycyclic cores (triphenylenes, hexaazatriphenylenes, triazatruxenes, tristriazolotriazines:chapter 6) are discussed.

The arms of the selected compounds consist of olefinic double or acetylenic triple bonds and/or benzene rings.

According to the literature, various further building blocks for the core and the arms have been used. Prominent examples are truxene cores [387] and oligothienylene arms [388], for which comprehensive reports already exist. Fluorenediyl and porphyrindiyl segments represent further repeat units for the conjugated arms. The latter building blocks can also serve as cores. However, these systems are beyond the scope of this article, which is defined in chapter 1.

The preparation of the monomeric or oligomeric [n]stars (n = 3, 4, 6) with emphasis on the final reaction steps is discussed in the Scheme 1, Scheme 2, Scheme 3, Scheme 4, Scheme 5, Scheme 6, Scheme 7, Scheme 8, Scheme 9, Scheme 10, Scheme 11, Scheme 12, Scheme 13, Scheme 14, Scheme 15, Scheme 16, Scheme 17, Scheme 18, Scheme 19, Scheme 20, Scheme 21, Scheme 22, Scheme 23, Scheme 24, Scheme 25, Scheme 26, Scheme 27, Scheme 28, Scheme 29, Scheme 30, Scheme 31, Scheme 32, Scheme 33, Scheme 34, Scheme 35, Scheme 36, Scheme 37, Scheme 38, Scheme 39, Scheme 40, Scheme 41, Scheme 42, Scheme 43, Scheme 44, Scheme 45 and Scheme 46. Despite of the large number of here described compounds, there is a big challenge for novel syntheses. The majority of conceivable combinations of core structures and repeat units for the arms is still unknown.

Among the spectroscopic properties of the star-shaped systems, the UV/Vis/NIR absorption and emission play an important role, because the long-wavelength transitions reflect the conjugation effect.

In comparison to linear conjugated chains, the [n]stars have a higher solubility and better film-forming properties. Their applications in materials science are wide-spread. The high π electron density and the π stacking tendency conveys the systems interesting optical, electrical and optoelectronic properties. Solar cells, field-effect transistors, light-emitting diodes, devices, which make use of non-linear optics including two-photon absorption, are typical application areas. Quite new is the use of supramolecular [n]star structures that are able to intercalate and release dye molecules [389].

Self-organization and (rotated) π stacking in crystals and liquid crystals are certainly outstanding properties of [n]stars in future multidisciplinary research and development projects. This review shall be a contribution to stimulate such projects.
